# Assessing Optimal Target Populations for Influenza Vaccination Programmes: An Evidence Synthesis and Modelling Study

**DOI:** 10.1371/journal.pmed.1001527

**Published:** 2013-10-08

**Authors:** Marc Baguelin, Stefan Flasche, Anton Camacho, Nikolaos Demiris, Elizabeth Miller, W. John Edmunds

**Affiliations:** 1 Immunisation, Hepatitis and Blood Safety Department, Public Health England, London, United Kingdom; 2 Centre for the Mathematical Modelling of Infectious Diseases, Department of Infectious Disease Epidemiology, London School of Hygiene & Tropical Medicine, London, United Kingdom; 3 Department of Mathematics and Statistics, University of Strathclyde, Glasgow, United Kingdom; 4 Department of Statistics, Athens University of Economics and Business, Athens, Greece; University of Hong Kong, Hong Kong

## Abstract

**Background:**

Influenza vaccine policies that maximise health benefit through efficient use of limited resources are needed. Generally, influenza vaccination programmes have targeted individuals 65 y and over and those at risk, according to World Health Organization recommendations. We developed methods to synthesise the multiplicity of surveillance datasets in order to evaluate how changing target populations in the seasonal vaccination programme would affect infection rate and mortality.

**Methods and Findings:**

Using a contemporary evidence-synthesis approach, we use virological, clinical, epidemiological, and behavioural data to develop an age- and risk-stratified transmission model that reproduces the strain-specific behaviour of influenza over 14 seasons in England and Wales, having accounted for the vaccination uptake over this period. We estimate the reduction in infections and deaths achieved by the historical programme compared with no vaccination, and the reduction had different policies been in place over the period. We find that the current programme has averted 0.39 (95% credible interval 0.34–0.45) infections per dose of vaccine and 1.74 (1.16–3.02) deaths per 1,000 doses. Targeting transmitters by extending the current programme to 5–16-y-old children would increase the efficiency of the total programme, resulting in an overall reduction of 0.70 (0.52–0.81) infections per dose and 1.95 (1.28–3.39) deaths per 1,000 doses. In comparison, choosing the next group most at risk (50–64-y-olds) would prevent only 0.43 (0.35–0.52) infections per dose and 1.77 (1.15–3.14) deaths per 1,000 doses.

**Conclusions:**

This study proposes a framework to integrate influenza surveillance data into transmission models. Application to data from England and Wales confirms the role of children as key infection spreaders. The most efficient use of vaccine to reduce overall influenza morbidity and mortality is thus to target children in addition to older adults.

*Please see later in the article for the Editors' Summary*

## Abstract 2

Marc Baguelin and colleagues use virological, clinical, epidemiological, and behavioral data to estimate how policies for influenza vaccination programs may be optimized in England and Wales.

*Please see later in the article for the Editors' Summary*

## Editors' Summary

**Background:**

Every winter, millions of people catch influenza, a viral infection of the airways. Most infected individuals recover quickly, but seasonal influenza outbreaks (epidemics) kill about half a million people annually. In countries with advanced health systems, these deaths occur mainly among elderly people and among individuals with long-term illnesses such as asthma and heart disease that increase the risk of complications occurring after influenza virus infection. Epidemics of influenza occur because small but frequent changes in the influenza virus mean that an immune response produced one year through infection provides only partial protection against influenza the following year. Annual immunization with a vaccine that contains killed influenza viruses of the major circulating strains can greatly reduce a person's risk of catching influenza by preparing the immune system to respond quickly when challenged by a live influenza virus. Consequently, many countries run seasonal influenza vaccination programs that, in line with World Health Organization recommendations, target individuals 65 years old and older and people in high-risk groups.

**Why Was This Study Done?:**

Is this approach the best use of available resources? Might, for example, vaccination of children—the main transmitters of influenza—provide more benefit to the whole population than vaccination of elderly people? Vaccination of children would not directly prevent as many influenza-related deaths as vaccination of elderly people, but it might indirectly prevent deaths in elderly adults by inducing herd immunity—vaccination of a large part of a population can protect unvaccinated members of the population by reducing the chances of an infection spreading. Policy makers need to know whether a change to an influenza vaccination program is likely to provide additional population benefits before altering the program. In this evidence synthesis and modeling study, the researchers combine (synthesize) longitudinal influenza surveillance datasets (data collected over time) from England and Wales, develop a mathematical model for influenza transmission based on these data using a Bayesian statistical approach, and use the model to evaluate the impact on influenza infections and deaths of changes to the seasonal influenza vaccination program in England and Wales.

**What Did the Researchers Do and Find?:**

The researchers developed an influenza transmission model using clinical data on influenza-like illness consultations collected in a primary care surveillance scheme for each week of 14 influenza seasons in England and Wales, virological information on respiratory viruses detected in a subset of patients presenting with clinically suspected influenza, and data on vaccination coverage in the whole population (epidemiological data). They also incorporated data on social contacts (behavioral data) and on immunity to influenza viruses in the population (seroepidemiological data) into their model. To estimate the impact of potential changes to the current vaccination strategy in England and Wales, the researchers used their model, which replicated the patterns of disease observed in the surveillance data, to run simulated epidemics for each influenza season and for three strains of influenza virus under various vaccination scenarios. Compared to no vaccination, the current program (vaccination of people 65 years old and older and people in high-risk groups) averted 0.39 infections per dose of vaccine and 1.74 deaths per 1,000 doses. Notably, the model predicted that extension of the program to target 5–16-year-old children would increase the efficiency of the program and would avert 0.70 infections per dose and 1.95 deaths per 1,000 doses.

**What Do These Findings Mean?:**

The finding that the transmission model developed by the researchers closely fit the available surveillance data suggests that the model should be able to predict what would have happened in England and Wales over the study period if an alternative vaccination regimen had been in place. The accuracy of such predictions may be limited, however, because the vaccination model is based on a series of simplifying assumptions. Importantly, given that influenza vaccination for children is being rolled out in England and Wales from September 2013, the model confirms that children are key spreaders of influenza and suggests that a vaccination program targeting children will reduce influenza infections and potentially influenza deaths in the whole population. More generally, the findings of this study support wider adoption of national vaccination strategies designed to block influenza transmission and to target those individuals most at risk from the complications of influenza infection.

**Additional Information:**

Please access these websites via the online version of this summary at http://dx.doi.org/10.1371.journal.pmed.1001527.

- The UK National Health Service Choices website provides information for patients about seasonal influenza and about vaccination; Public Health England (formerly the Health Protection Agency) provides information on influenza surveillance in the UK, including information about the primary care surveillance database used in this study
- The World Health Organization provides information on seasonal influenza (in several languages)
- The European Influenzanet is a system to monitor the activity of influenza-like illness with the aid of volunteers via the Internet
- The US Centers for Disease Control and Prevention also provides information for patients and health professionals on all aspects of seasonal influenza, including information about vaccination and about the US influenza surveillance system; its website contains a short video about personal experiences of influenza
- Flu.gov, a US government website, provides access to information on seasonal influenza and vaccination
- MedlinePlus has links to further information about influenza and about immunization (in English and Spanish)

## Introduction

Seasonal influenza is a serious public health problem globally. In countries with an advanced health system, most of the deaths occur among elderly adults and those with co-morbid conditions that place them at increased risk [1],[2]. Immunisation strategies can target individuals at high risk of complications and/or key spreaders in order to interrupt or reduce transmission. In most countries, influenza vaccination programmes have traditionally targeted individuals 65 y and older and those in high-risk groups, in line with World Health Organization recommendations [3]. Following the experience with pandemic H1N1/2009 influenza, priority groups for vaccination are now being reconsidered [4].

Children have been identified as the main spreaders of influenza infection [5],[6] and thus a potential target for vaccination, not only for their own protection but also for the indirect protection of others [7]–[9]. The annual mass vaccination of children presents major operational and resource challenges, and thus it is important for policy makers to be confident about the additional population benefits likely to accrue from inducing herd protection. The severity of each seasonal influenza epidemic is the result of a complex interplay between background population immunity, which is partly a function of exposure to previously circulating cross-reactive viruses; the nature and extent of contact between age groups; the pathogenicity of the circulating viruses; and the impact of vaccination, which in turn is dependent on coverage and the match between wild and vaccine strains. While vaccination programmes are designed with a long-term perspective, some of these parameters vary significantly from one season to another, necessitating longitudinal datasets to ensure that vaccine policy decisions are robust to year-to-year fluctuations. In addition, most of the available surveillance data are designed for healthcare monitoring and are difficult to integrate directly in dynamic transmission models (which require information on infection, rather than use of services). In the absence of quantitative estimates derived from epidemiological data, models used to test the impact of alternative vaccination programmes have thus made substantial assumptions about background immunity, structure of contacts, and transmissibility of the virus [7]–[10]. Furthermore, given the importance of high-risk groups in contributing to the overall burden of disease, modelling of the impact of vaccine-induced changes in transmission on burden of disease needs to take account of this additional population heterogeneity. Recently, models using Bayesian evidence synthesis have been developed to estimate the severity of influenza [11] and influenza infection attack rates [12],[13]. We combine similar Bayesian techniques with transmission models in a novel approach that provides evidence to inform vaccine policy decisions.

We thus apply a modern statistical approach to help disentangle the underlying biological, epidemiological, and behavioural factors that determine the annual patterns observed in surveillance data. We use the experience in England and Wales, where vaccination was targeted at high-risk groups until 2000, then extended to all individuals ≥65 y, as an exemplar. Six different sources of data are used. We use demographic data to define the structure of the population in terms of age and risk groups. The structure of contacts between age groups is inferred from a contact survey. The outcomes of the model are fitted to time series of healthcare consultations complemented by virological surveillance and informed by vaccine uptake and match data. Finally, links between infections and consultations are given priors using serology data. By synthesising the evidence from these multiple data sources across 14 influenza seasons in England and Wales, we quantify the amount of transmission due to each age group and assess the impact of past and proposed changes to immunisation policy on cases and deaths.

The hypotheses we examine are whether, given the vaccination coverage currently achieved in high-risk groups and elderly adults in industrialized countries, there has been any material impact on transmission and whether a more efficient deployment of vaccine in terms of overall population morbidity and mortality would be to target children as the key infection spreaders. The incremental benefit of vaccinating low-risk children and/or adults as an addition to the existing risk/age-based policy is examined for different coverage levels.

## Methods

### Demography

For each of the seasons of the study, the number *W_i_* of individuals in the population with age *i* is taken from the Office for National Statistics (http://www.ons.gov.uk). Seven age groups were considered (1, 1–4, 5–14, 15–24, 25–44, 45–64, 65+ y). Each of these age groups is divided into individuals at low or high risk of complications associated with influenza, later simply referred to as low or high risk. Individuals are considered at high risk if they have one of the following conditions: chronic respiratory, heart, or renal disease; diabetes; or immunosuppression due to disease or treatment. The proportion of people in a risk group in a particular age group is assumed to be constant over the period of the study. We derived the proportion of individuals in a risk group for each age group by analysing data from the Royal College of General Practitioners (RCGP) Weekly Returns Service over a period of 5 y (2003–2008). The numbers of people in the different age groups and the percent classified as high risk are given in Table 1. On the last line of the table, the actual numbers resulting from the RCGP analysis can be seen.

**Table 1:** Sizes of the modelled population compartments by age and risk group averaged over the period of the study.

Modelled Population	Age Group						
	1 y	1–4 y	5–14 y	15–24 y	25–44 y	45–64 y	65+ y
Average age group size (2003–2008)	623,779	2,525,322	6,608,728	6,603,485	15,214,371	12,578,021	8,423,248
Percent at high risk	2.1%	5.5%	9.8%	8.7%	9.2%	18.3%	45.0%
Proportion at high risk, from RCGP survey^a^	723/33,780	15,837/287,075	77,197/783,844	63,206/724,292	165,827/1,803,008	301,562/1,647,783	456,654/1,013,979

a Number of individuals at high risk of complications associated with influenza/total number of individuals surveyed.

### Contact Survey

In 2006, a pan-European survey was conducted to measure and compare the structure of contacts in eight different European countries (Belgium, Finland, Germany, United Kingdom, Italy, Luxembourg, the Netherlands, and Poland). Attention was paid to recruiting participants representative in terms of geography, age, and sex [14]. Recruitment methods differed between countries, but were based in the United Kingdom on face-to-face interviews. Participants were asked to complete diaries recording with whom they had contacts during a day. Diaries also recorded different information relative to those contacts. Among them, the age of the contact was recorded, the nature of the contact (conversational or physical contact) and the nature of the day (weekday, weekend, or holiday). Details about the project can be found in Mossong et al. [15]. We use data from the United Kingdom arm of the study: the final data consisted of 1,012 participants aged 0 to 79 y who recorded 11,876 contacts in total.

### Consultations in General Practices

Since January 1967, the Weekly Returns Service of the RCGP has monitored the activity of acute respiratory infections in general practices. As part of this scheme, the weekly number of persons consulting for influenza-like-illness (ILI) is recorded. For each week of the studied period (from the influenza season 1995/1996 until 2008/2009), we obtained the size of the monitored population included in the RCGP Weekly Return Service and the number of individuals in that monitored population consulting for ILI stratified in five age groups (0–4, 5–14, 15–44, 45–64, 65+ y). For a given season, we note by 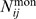 the size of the monitored population and by *m_ij_* the number of people recorded as consulting for ILI in age group *i* at week *j* in that population.

In 2008, roughly 1.7% of the total population in England and Wales was included in the RCGP network of practices. Although this number might seem small in comparison with other existing surveillance networks, this sample is considered to be reasonably representative of the whole population in terms of demography and geography [16].

### Respiratory Virus RCGP Surveillance

In order to complement the syndromic surveillance, a new surveillance activity of virological confirmation of cases was set up starting from the 1995/1996 season. During weeks of potential influenza activity, samples are taken from people presenting with ILI and sent for testing to the Respiratory Virus Unit of the Health Protection Agency. In the period considered (i.e., from the start of the virological surveillance activity until the season 2008/2009), the dataset includes 12,575 virological samples with recorded age group tested, which corresponds to an average of 900 samples per season. In total, however, during the last season, because of the novel H1N1 pandemic, 3,395 samples were tested. If this season is excluded, the dataset has an average of 700 samples per season.

The predominant strain in the period investigated was A/H3N2. In every season except 2000/2001, A/H3N2 was identified, while in several seasons either A/H1N1 or B was absent. In any given week, the number of confirmed samples by age group was relatively small. In addition, no samples were taken in the age group 1 y; most of the samples (47.7%) were taken in the 15–44-y age group, while the younger age groups—though higher transmitters—are less represented (9.6% and 13.7% for the age groups 1–4 y and 5–14 y, respectively). Elderly adults (65+ y) are under-represented (7.8%), which is a problem as the incidence appears to be smaller in this age group. Positivity rates are difficult to accurately quantify in this age group.

Among these tested samples, 17.3% were positive for influenza (H3N2: 53.8%, H1N1: 11.9%, H1N2: 0.9%, H1N1pdm: 10%, B: 18.9%). In the 2002/2003 season, only seven samples were taken, among which five were positive. However, three out of the five samples were taken in one age group (1–4 y) at a time when the level of ILI was extremely low (middle of May 2003).

In the rest of the manuscript, we note by 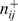 the number of positive samples from a given subtype (A/H1N1, A/H3N2, or B) taken among the *n_ij_* samples tested at week *j* in the age group *i*.

### Vaccination Uptake and Match

Coverage by age and risk group by week was taken from Joseph et al. [17] for the seasons before 2003/2004. Figures for the 65+-y age group from 2004/2005 onwards were taken from the Health Protection Agency/Department of Health (HPA/DH) annual reports on the influenza programme [18].

In order to be able to derive estimates of the vaccine efficacy for each strain and season, data from the Health Protection Agency were used to establish the match between the circulating and vaccine strains (Table 2).

**Table 2:** Match of the vaccine strains to the circulating seasonal strains during the period 1995/1996 to 2008/2009.

Strain	Season													
	1995/1996	1996/1997	1997/1998	1998/1999	1999/2000	2000/2001	2001/2002	2002/2003	2003/2004	2004/2005	2005/2006	2006/2007	2007/2008	2008/2009
A/H3N2	U	M	U	M	M	U	M	M	U	U	M	M	M	M
A/H1N1	M	U	M	U	U	M	M	M	M	M	M	M	M	M
B	U	U	U	U	U	U	U	U	U	U	U	U	U	U

M, matched vaccine and circulating seasonal strains; U, unmatched vaccine and circulating seasonal strains.

### Serology for the Season 2003/2004 in A/H3N2

During the winter of 2003/2004, the United Kingdom experienced an unusual level of influenza activity following the emergence of A/Fujian/411/02-like antigenic variant strains. In order to investigate the severity of these new drifted strains, an age-stratified serological survey was conducted by the Health Protection Agency using sera sampled before and after the season [19]. Resulting data thus contain information on haemaglutination inhibition titres and RCGP age group for 875 sera collected pre- and post-season. The haemaglutination inhibition assays were performed using the A/Wyoming/3/03 strain, antigenically equivalent to the A/Fujian/411/02 strain that was circulating in the United Kingdom during the 2003/2004 season.

### Methods Overview

Based on these data sources, an inference and modelling framework embedding social, immunisation, epidemiological, and surveillance components was developed. A Bayesian approach to statistical inference was adopted, as it provides the most suitable framework for synthesising diverse sources of evidence in a coherent manner [20]. Specifically, we utilised adaptive Markov chain Monte Carlo (MCMC) techniques [21], which represent a generic tool for inference from complex stochastic systems. An attractive aspect of the Bayesian approach when coupled to the MCMC methodology is its particular suitability for the natural propagation of uncertainty. This is especially crucial in the present analysis since the quantities of interest, like the final numbers of individuals infected under different scenarios, are non-linear functionals of the basic model parameters. Additionally, MCMC is highly modular, thus accommodating the inclusion of a novel algorithm for exploring the space of contact matrices (i.e., rates of epidemiologically relevant contacts).

**Figure 1:**
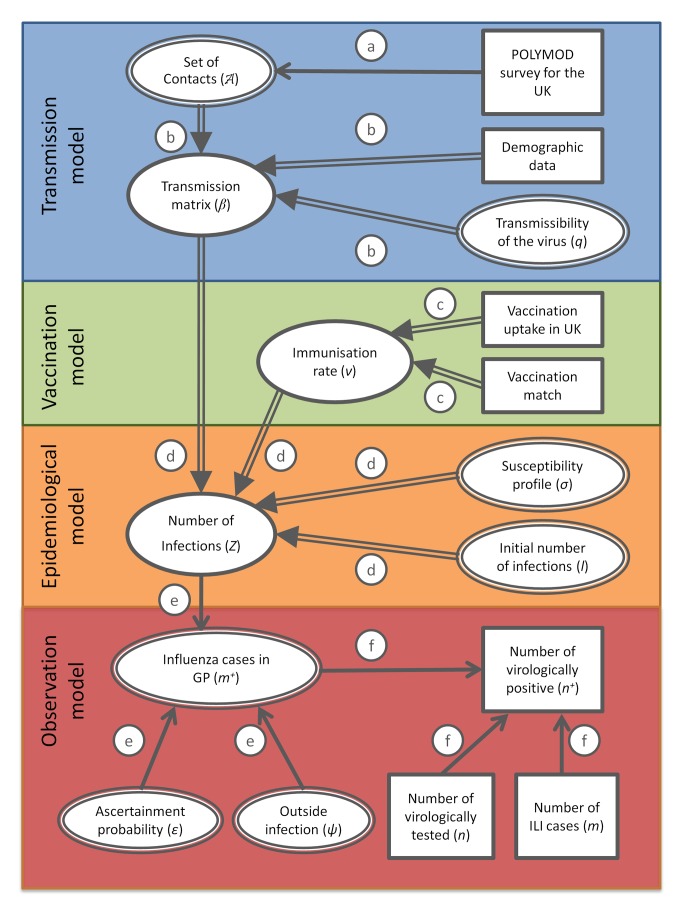
Directed acyclic graph showing the link between the different modelling components, data, and parameters. Double and simple arrows indicate, respectively, deterministic and stochastic relationships, ellipses indicate variables, and rectangles indicate data. The circled letters indicate which relationship connects the variables or data involved. These relationships are as follows: process a, drawing with replacement; process b, calculation of the transmission matrix by rescaling the mixing matrix obtained by reweighting for age and weekday and weekend days; process c, derivation of the immunisation rate from uptake data and match of vaccine; process d, integration of the SEIR model of transmission; process e, ascertainment of cases through ILI recording at GPs; process f, virological testing scheme following a hypergeometric distribution.

We use a directed acyclic graph [22] to represent the model structure and the way the data streams are integrated in the inference scheme (Figure 1). In what follows we refer to the notation adopted in Figure 1 in order to identify the different processes operating at the different levels (transmission, vaccination, epidemiology, and observational processes) of the model.

### Transmission Model

A series of studies have linked routes of transmission for infectious diseases with the structure of contacts in the community [15],[23]. The assumption is that when in contact with a susceptible individual, an infectious individual will have a probability *q* of transmitting the disease. This transmission probability *q* will depend on the type of contact involved (e.g., in the case of a respiratory virus, a physical contact might be more effective in transmitting than a conversational contact of the same duration) and the type of pathogen.

Sociological surveys can thus replace expert opinion [24] to characterise transmission matrices. However, though well established for sexually transmitted diseases, where the implication and interpretation of “contacts” is more obvious, the notion of “contact” is problematic for other diseases such as respiratory infections. Additionally, because of sample size or biases, the results may need some reworking in order to become directly interpretable. For example, we demonstrated previously that the transmission matrix of the A/H1N1/2009 virus directly inferred from the mean contact matrix derived from the POLYMOD study could not explain the change of dynamics during the summer holidays [25]. However, using matrices produced from resampling (with replacement) the original data did allow the dynamics to be accurately captured.

We thus assume here that there exists among the possible sets a set of resampled participants (with some participants sampled several times) that represents an appropriate structure of contacts in the population in terms of disease transmission. The mixing in our model is thus described by a resampled subset of participants from the original UK POLYMOD dataset. From this list of participants, we uniquely derive a mixing matrix using the methodology developed below (similar to the one described in Wallinga et al. [23] and Hens et al. [14]).

For a set of entries resampled from the original POLYMOD dataset, let *T_j_* be the number of participants in class *j*, *A_k_* and 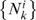 be, respectively, the age and number of contacts made in age group *i* for and by participant *k*. We can then associate to each participant *k* a weight *w_k_* depending on participant age and the day recorded in the diary:
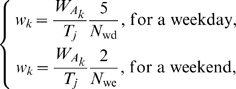
(1)with *N*_wd_ and *N*_we_ the total number of contacts recorded, respectively, during week days and weekends, and 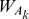 the number of individuals of age *A_k_*.

The re-normalised average number of contacts per day *d_ij_* made by participants from age group *j* with persons in age group *i* standardised for age and weekdays is
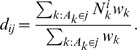
(2)As contact making is symmetric, the number of contacts in the population resulting from people from age group *i* meeting with people from age group *j* is the same as the number of contacts made by people from age group *j* meeting with people from age group *i*. If we call *c_ij_* the probability that two randomly selected individuals in group *i* and *j* get in contact, we get by symmetry *c_ij_ = c_ji_*. By using the direct formula *c_ij_ = d_ij_*/T_i_, symmetry will not usually be achieved because of reporting or participation biases. To achieve symmetry of the contact matrix {*c_ij_*}, we thus set
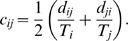
(3)To obtain the transmission matrix, we finally multiply this mixing matrix by *q*, describing the transmissibility of the virus, which is the probability that a contact between an infectious person and a susceptible person leads to transmission. The transmission matrix is thus broken down into its biological and social components.

In this study, we consider an average contact matrix over the epidemic season, thus not considering holiday periods or weekends. When the epidemic is simultaneous with long periods of holidays (as during the A/H1N1/2009 pandemic in the United Kingdom), it is necessary to make the distinction between term and holiday contact matrices [25].

At each step in the MCMC chain the matrix is updated by resampling from the POLYMOD data with replacement (Figure 1, process a) and is thus inferred within the MCMC algorithm via a novel random-walk type of proposal on the matrix space where the number of contacts between distinct age groups is re-normalised, reducing potential reporting or participation biases via symmetry. The contact matrix is combined with the demographic data and scaled by the transmissibility of the virus to give the transmission matrix of the virus (Figure 1, process b).

### Vaccination Model

The different epidemiological compartments of the model are split into two types of compartments based on vaccine history (indexed by *N* for naive or *V* for vaccinated; see Figure S1 in Text S1). As the vaccine is not 100% effective, a proportion α*_i_* of the vaccinees become protected (we assume full protection), while the rest 1−α*_i_* remain fully susceptible. The vaccine efficacy α*_i_* depends on age group and the degree of match between the strain in the vaccine and the circulating strain in that year.

As in [25], we assume a 2-wk delay between infection and development of protective antibodies. This is based on an analysis of the dynamics of seroconversion following cases of A/H1N1pdm infection confirmed by PCR [26]. We do not model the dynamics of antibody production as in [12] as these measurements are done for cases following infection rather than vaccination, and vaccine trials usually measure level of antibodies after a longer period. The period of 2 wk to confer protection appears to be a reasonable assumption for our purpose.

Combining the vaccination uptake and the vaccination match results in a time varying immunisation rate *v* *_ik_* in age group *i* and risk group *k* (process c). The rate of immunisation *v* *_ik_* is assumed to be constant over a monthly period. The total number of persons immunised in each age group by month is taken from Joseph et al. [17] and HPA/DH reports. For the purpose of the transmission fitting, we assume that the change of status (whether as *S^V^* or *R^V^*) occurs two weeks after vaccination.

A recent Cochrane review [27] suggested that vaccine efficacy was 73% in years in which the vaccine was well matched, and 44% in years when there was a poor match between the vaccine and circulating strains. In addition, a recent analysis by Fleming et al. [28] on seasonal influenza vaccine efficacy suggested that efficacy was lower in elderly (46%) compared to younger adults (70%). Since all of the studies included in the Cochrane review were performed on healthy young adults, we assume that efficacy was 70% and 46% in those under 65 y and 65 y or older, respectively, in a well-matched year, which was reduced to 42% and 28% in poorly matching years. We assume that children were immunised with a live attenuated influenza vaccine and that this type of vaccine produced protection similar to that of the current trivalent inactivated vaccine in adults.

When the age grouping of the coverage data differed from that used in our model, we reweighted the coverage values proportionally to the population sizes. Figures for the 65+-y age group from 2004/2005 onwards are taken from the HPA/DH annual report on the influenza programme [29]. For the coverage in high-risk individuals under 65 y for the season 2007/2008, we used the HPA/DH report reweighted depending on the risk group. For the seasons 2004/2005, 2005/2006, and 2006/2007, the coverage in each age group is taken as the figure from 2007/2008 rescaled by the ratio of coverage in the total non-risk under-65-y population in each year and in 2007/2008. S1 in Text S1 gives the final coverage by age/risk group and season assumed in the analysis.

Only infants over 6 mo received vaccination. Infants under 6 mo were assumed to be fully susceptible. Possible protection by remaining maternal antibodies was not considered.

### Epidemiological Model

The transmission matrix, the immunisation rate, the susceptibility profile, and the initial number of infections in the different age groups feed into the epidemiological model (Figure 1, process d). The susceptibility profiles and initial number of infections are derived from the inference procedure. The epidemiological model uses an age- and risk-specific SEIR (susceptible–exposed–infected–recovered) epidemic model [25] with gamma-distributed latent and infectious periods. We assumed that the background immunity in the population (inferred by the susceptibility profiles) results in a reduced probability of infection rather than full protection.

The epidemiological model describing the transmission dynamics of the virus is similar to that used during the 2009 pandemic [25]. The model has a modified SEIR structure. We assume random mixing (within an age group) between the clinical groups. The size of these groups is given for each season by demographic figures from the Office for National Statistics (average figures over the period of the study are presented in Table 1). To allow the latent and infectious periods to be gamma distributed (rather than exponential), we assume that each of the E and I compartments are defined by two classes (hence SEEIIR structure), with the same rate of loss of latency (γ_1_) and infectiousness (γ_2_) in both groups. Hence, the average latent period is 2/γ_1_, and the average infectious period is 2/γ_2_. Following Ferguson et al. [30], we chose γ_1_ = 2.5 and γ_2_ = 1.1, corresponding to a latent period of 0.8 d and an infectious period of 1.8 d.

We assume that at the outset of the influenza epidemic a small fraction of individuals in each age class is infectious, and the remainder are susceptible. This fraction is obtained by scaling the initial infectious population by a factor *l*. Preseasonal susceptibility is not necessarily assumed to be absolute, and may vary by age. An age-dependent susceptibility profile {σ*_i_*} is assumed, the parameters of which are estimated from the model-fitting process for all strains and years, except for H3N2 in 2003/2004, for which a pre-epidemic serological profile was available [19] (more detail about the derivation of susceptibility priors for this year is given in Section 2.2.3 of Text S1). As susceptibility has to be inferred, we chose to limit the model to three age bands to avoid overfitting. We considered an average susceptibility for children (0–14 y old), younger adults (15–64 y old), and elderly adults (65+ y old).

Uncertainty in estimates of these quantities reflects the joint uncertainty in the parameters from which they are derived. Therefore, any correlation structures that may be present are appropriately propagated. We start our model at week 35 of the epidemiological season (rather than week 40, which is usually considered as the starting date of the flu season) to match the start of the epidemic with the reopening of schools, the period at which we considered that ILI starts to increase.

The equations of the SEIR epidemiological model are given by Equation 1 in Text S1. The models age-group-specific force of infection is given by
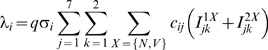
(4)where *q* is the transmissibility parameter, *c_ij_* is the rate at which individuals in age group *i* make contact with those in age group *j*, and σ*_i_* is the susceptibility of age group *i*.

The incidence Z_ik_(*n*) of new infections in age group *i* and risk group *k* at week *n* is
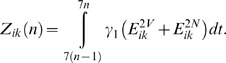
(5)Note that the general practitioner (GP) consultation and swabbing data are only available in five age groups. The seven age groups in the epidemic model are thus collapsed into these five age groups for the purpose of the observation model, by combining the 1 and 1–4 y age groups and the 15–24 and 25–44 y age groups. (For simplicity of notation, we do not explicitly write the step that consists of collapsing the seven age groups into five. This leads us to a slight abuse of notation. The *i* index of *Z*_ik_(*n*) in the epidemic model is varying between 1 and 7 and is thus different from the *i* index of *Z*_ik_(*n*) in the observation model, which is varying between 1 and 5. Passing from one to the other is simply done by adding the sizes of the populations that are being grouped.)

### Observation Model

The last step of the model is to link syndromic surveillance data with the number of infections due to circulating influenza viruses in the population. Although GP consultations for ILI are often used as a proxy to monitor transmission of influenza in a population, individuals consulting for ILI and individuals infected with one of the circulating strains from the influenza family are typically two different sets. A large proportion of individuals recorded as having ILI by their GP consult for symptoms resulting from infection by pathogens other than influenza (e.g., respiratory syncytial virus). Also, during an influenza epidemic, only a fraction of cases present symptoms [31], among whom only a fraction will consult in general practice. Of them, another fraction will be recorded as ILI (others being recorded as having other respiratory symptoms), and if PCR-tested, not all of them will end up positive because of the sensitivity of the test. We derive hereafter a statistical model to rigorously link syndromic surveillance and influenza infections in the population.

The reappearance of influenza in temperate countries from one year to another is determined by the introduction by people travelling of some of the new variants from strains circulating globally [32]. Patterns of circulation between the two global hemispheres and persistence in some regions appear to be governing the global circulation of influenza. In a country such as the United Kingdom, this translates into exposure throughout the year to importation from travellers. The H1N1/2009 pandemic, with its intense testing and tracking of travellers acquiring influenza abroad, shed light on the pattern of introduction of the virus. In-country epidemics of influenza are characterised by a balance of constant re-introduction from outside and local in-country transmission until a widespread epidemic emerges (for a comparative description of the initial phase of the epidemic during the 2009 spring in 12 European countries, see [33]).

We thus considered that in addition to the main epidemic modelled by the (deterministic) equations described in the previous section, each individual in the monitored population is associated with a weekly risk of being infected outside of the national (deterministic) epidemic that we are modelling. This risk can be associated with travelling abroad (e.g., in the southern hemisphere during the influenza season in the northern summer) or with a local outbreak independent of the national one. This risk is modelled by a probability ψ that we assume, for simplicity, is independent of time and age.

If, for ease of exposition, we describe by θ the parameters of the epidemic model, the incidence among the monitored population of age group *i* at week *j* of new cases arising from the main deterministic epidemic (assumed to be homogeneous across the country) is
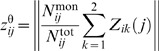
(6)with θ the epidemiological parameters (*q*, σ*_i_*, *l*, and *A*), 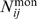 and 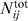 the size of, respectively, the monitored and the total population in age group *i* and week *j*, and 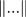 the function rounding to the nearest integer.

At the same time, the incidence of infection acquired from outside of the main epidemic follows a binomial law of probability ψ:
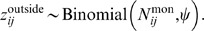
(7)The ascertainment of a case by the surveillance system is dependent on two steps. First, the infected case needs to go to the GP, and the GP needs to record the case as ILI. Second, a swab sample has to be taken and confirmed as containing influenza viruses. Because of these two steps, the number of ascertained cases is usually much smaller than the number of ascertainable cases: not all persons presenting with ILI are tested for influenza. The number of ascertained cases depends on the testing scheme, while the number of ascertainable cases depends on the number of individuals with true influenza infections in the population that present to their GP. We thus are interested in the number of ascertainable cases and assume that each individual in age group *i* infected by the strain of influenza studied has a probability *i* of being ascertainable, i.e., going to the GP, being recorded as ILI, and having a detectable viral load (the number of ascertainable cases is thus the number of positives that we would get if all the patients with ILI were virologically tested for influenza in a given week).

The number of ascertainable cases that get infected outside of the main epidemic can then be modelled as binomial of probability 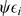 as the number of cases with infection from outside is a binomial of probability ψ, and the number of ascertained cases, conditional on the number of cases, is also binomial with probability 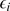:
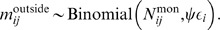
(8)As ψε*_i_* is small and 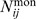 is big, 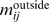 can less cumbersomely be defined by a Poisson distribution of rate 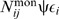:
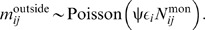
(9)This approximation is accurate for all the values considered in this paper.

**Figure 2:**
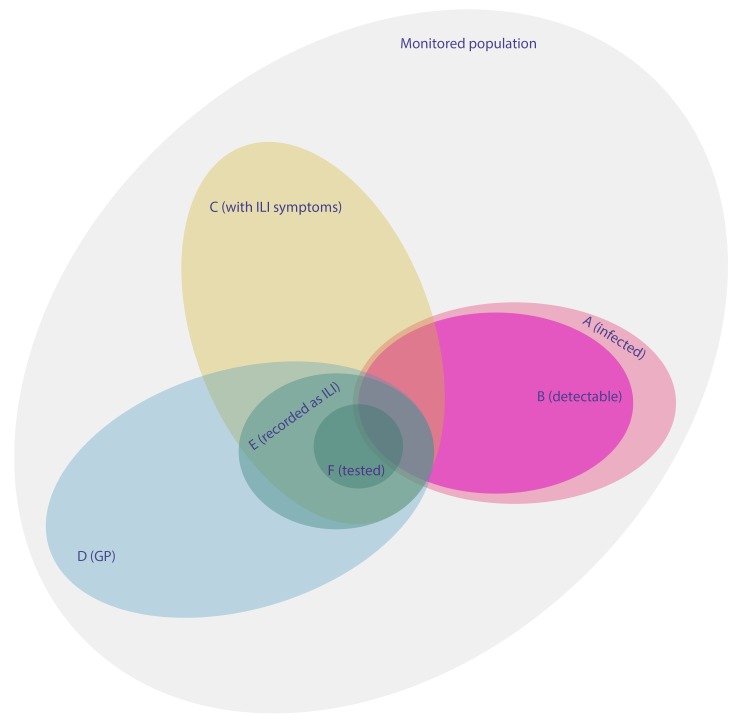
Venn diagram giving a schematic representation of the different surveillance schemes and clinical statuses. The relative proportions of the different sets vary from one week to another and are different for each age group.

Among the *m_ij_* individuals reported with ILI at week *j* among age group *i*, we are interested in the 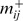 who have a detectable viral infection for a certain subtype of influenza. We assume that they are a binomial sample from the total number of infected (equal to 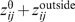) with ascertainment probability 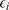, assumed to be constant over time (Figure 1, process e). The virological testing scheme can be seen as randomly drawing *n_ij_* samples without replacement from the population of the *m_ij_* individuals consulting for ILI, in which 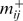 have a virologically detectable infection. The number of positive samples 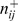 that are expected can thus be represented using a hypergeometric distribution (Figure 1, process f). The consultation and positivity model is then described by the following system of equations:
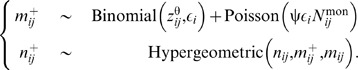
(10)A representation of the surveillance system in terms of sets is given in Figure 2. Following this set representation, we can express 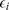 as a product of elementary epidemiological quantities (derivation in Text S1):
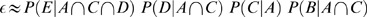
(11)where *P*(*E*|*A* ∩ *C* ∩ *D*) is the GP recognition ability, *P*(*D*|*A* ∩ *C*) the propensity to consult among symptomatic influenza cases, *P*(*C*|*A*) the proportion with symptoms among the infected, and *P*(*B*|*A* ∩ *C*) the sensitivity of the test against clinical influenza cases.

Using this, we can derive an order of magnitude for 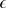. In Table 3, we summarise the range of values that could take the elementary epidemiological parameters forming 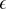 and obtain values between 0.006 and 0.05.

**Table 3:** Order of magnitude of the ascertainment probability ε derived from the elementary probabilities of measurable underlying phenomena.

Quantity	Notation	Range	Source
Sensitivity of virological test	*P*(*B*|*A* ∩ *C*)	0.6–0.9	Assumption
Proportion of clinical cases among infections	*P*(*C*|*A*)	0.3–0.51	[31]
Propensity to consult among clinical cases	*P*(*D*|*A* ∩ *C*)	0.05–0.12	[39]
Sensitivity of the GP diagnostic	*P*(*E*|*A* ∩ *C* ∩ *D*)	0.65–0.9	Assumption
Ascertainment probability	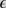	0.006–0.05	Derived

### Reproduction Numbers and Mixing between Groups

The transmission potential of a pathogen is traditionally summarised by way of its basic reproduction number *R*_0_, the average number of secondary cases following the introduction of an infectious individual in a totally susceptible large population [34]. In a heterogeneous population, *R*_0_ will be a function of each of the reproduction numbers within population sub-groups and of the degree of assortativity of these sub-populations. As a result, similar *R*_0_ values can arise from very different epidemiological situations (see Section 2.1.4 of Text S1). We thus measured, in addition to the basic reproduction number for the overall population, the specific reproductive numbers for children under 15 y and adults (*R*_C_ and *R*_A_, respectively) and the degree of assortativity *d_R_* of the two subpopulations.

### Generating Alternative Vaccination Scenarios

To estimate the impact of potential changes to the current vaccination strategy, we ran for each of the 14 seasons and each of the three strains, 1,000 simulated epidemics with parameters sampled from the posteriors obtained using MCMC techniques. In addition to the actual strategy (used in the fitting process), we analysed an additional ten strategies. The ten strategies are three “basic” strategies and incremental extensions of them (at 15%, 30%, 50%, and 70% coverage):

- **No vaccination:** Nobody receives any vaccine doses.
- **Pre-2000:** The pre-2000 scheme is kept throughout the 14 y, i.e., the risk groups are targeted as they were during these years; the change to targeting the low-risk 65+-y group that occurred in 2000 is not implemented. For the seasons 2000/2001 to 2008/2009, the average coverage from the pre-2000 seasons is kept for this group (29.34% coverage in the low-risk 65+-y group).
- **Post-2000:** The post-2000 scheme is applied throughout the 14 y, i.e., the risk groups are targeted as they were during these years; the low-risk 65+-y group is targeted in all seasons. For the seasons 1995/1996 to 1999/2000, the coverage of 1999/2000 is applied for this group (70% coverage in the low-risk 65+-y group).
- **S1:** Low-risk 0.5–4-y-olds are vaccinated at 15%, 30%, 50%, and 70% coverage, incremental on the post-2000 scenario.
- **S2:** Low-risk 50–64-y-olds are vaccinated at 15%, 30%, 50%, and 70% coverage, incremental on the post-2000 scenario.
- **S3:** Low-risk 5–16-y-olds are vaccinated at 15%, 30%, 50%, and 70% coverage, incremental on the post-2000 scenario.
- **S4:** S1+S2, i.e., combination of scenarios 1 and 2.
- **S5:** S1+S3, i.e., combination of scenarios 1 and 3.
- **S6:** S1+S2+S3, i.e., combination of scenarios 1, 2 and 3.
- **S7:** Universal, i.e., everybody among the low-risk population is vaccinated at 15%, 30%, 50%, and 70% coverage, incremental on the post-2000 scenario.

To quantify the efficiency of the assessed programmes, we measured the reduction in infections and deaths induced by one dose of vaccine for each of the different strategies. For this, we used the estimated total reduction in number of infections and deaths during the period (removing the season 2002/2003, where virological samples are too scarce) and divided it by the numbers of doses of vaccines given by the programme over the same period. For the extension strategies, we took the median over the four considered coverages (15%, 30%, 50%, and 70%).

### Estimation of the Number of Deaths due to Influenza

Our model provides estimates of the number of influenza infections (*N* ^infec^) under different vaccination scenarios for the period 1995–2009. In order to compare these scenarios on the basis of the predicted number of influenza deaths (*N* ^death^), we need the case fatality ratio (CFR = *N* ^death^/*N* ^infec^) of influenza. Ideally, because our model provides estimates of the *N* ^infec^ by strain (*s*), age (*a*), and risk (*r*) group we would like to use CFRs of influenza as a function of these three variables: CFR*_s,a,r_*. However, such estimates are currently lacking in the literature, mainly because quantifying the magnitude of both infection and mortality attributable to influenza is not straightforward [1].

In theory, it would be possible to extend our current framework to include some influenza mortality data in order to estimate *N* ^death^ and then deduce CFRs. Unfortunately, in contrast to the weekly age- and strain-specific data available for influenza morbidity, the best information on influenza mortality in England consists only in annual, age-specific estimates for the restricted period 1999–2009 [1]. In this context (i.e., only one data point per year for all three strains and only two-thirds of the period considered), it would be illusory to try to estimate annual CFR*_s,a,r_* with our dynamical model approach. Instead, we propose to combine available estimates of annual *N* ^death^ by age group in England—obtained from a recently published regression analysis by Hardelid et al. [1] (see Table S2 in Text S1)—with our estimates of the annual *N* ^infec^ in order to construct and fit generalised linear models whose coefficients equal the desired CFRs.

More precisely, because there was evidence of overdispersion in the mortality time series of Hardelid et al. [1], we start with the following age-specific negative binomial regression model with identity link and no intercept:
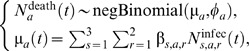
(12)where the negative binomial distribution is parametrized by its mean (μ*_a_*) and dispersion (ϕ*_a_*), the index *a* denotes the age group considered (we fit each age-specific model independently), and *t* runs over the restricted period 1999–2009. Given that 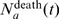 (Hardelid et al. data) and 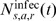 (our model outputs) are “known” (with uncertainty in both sources), the objective is to estimate the coefficients β*_s,a,r_* that, given the identity link and the absence of intercept, correspond to the desired CFR*_s,a,r_* values.

Note that the age groups in the study of Hardelid et al. [1] (15, 15–44, 45–74, 75+ y) do not exactly match those of our model (1, 1–4, 5–14, 15–24, 25–44, 45–64, 65+ y). We tackle this issue by (1) aggregating the three youngest age groups in our model and (2) refitting the Hardelid et al. regression model for the age groups 45–64 and 65+ y (H. Green, Health Protection Agency, personal communication).

In principle, because the severity of a strain is expected to change from one year to another, CFRs should also depend on the season *t*, thus greatly increasing the number of parameters. Here, we instead assume that the severity effect can be captured by the overdispersion parameter φ*_a_*. Such a simplification is required for two reasons: first, we have a single (uncertain) estimate of 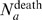 per year, and, second, these estimates are not available before the 1999/2000 season [1]. Put another way, we assume that the CFR*_s,a,r_* values are constant from 1999 to 2009 and can be applied to the 1995–1999 period.

In addition, one can see that each age-specific model (Equation 12) suffers from overparametrization as it intends to estimate six parameters (three strains times two risk groups) from only nine data points (of the ten seasons provided by the study of Hardelid et al. [1], only nine are used since our estimates of *N* ^infec^ for the 2002/2003 season are not reliable because of a lack of virological data during that particular season). To tackle this issue, we assumed that the risk ratio CFR*_s,a_*_,2_/CFR*_s,a_*_,1_ depends only on the age group *a* and is equal to R*_a_*: the age-specific risk ratio of the CFRs for acute respiratory infection among patients not in a high-risk group versus patients in a high-risk group. The R*_a_* values were obtained by analysing extracts from the Hospital Episode Statistics database (Health Social Care Information Centre) for England, for the period April 2000–March 2009. Patients were included when they had an acute respiratory ICD-10 diagnostic code (J0*, J1*, J2*, J3*, J40*, J41*, J42*, J43*, J44*, J47*) in any diagnostic fields and were divided into risk group/non-risk group based on a risk-group-related ICD-10 code. (We used the following ICD-10 codes for risk groups [where a two-digit code is given, it includes all ICD-10 codes beginning with that code]: D73, J4, J6, J7, J8, Q30, Q31, Q32, Q33, Q34, Q35, Q36, Q37, I05, I06, I07, I08, I09, I11, I12, I13, I20, I21, I22, I25, I27, I28, I3, I40, I41, I42, I43, I44, I45, I47, I48, I49, I5, I6, Q2, N0, N11, N12, N14, N15, N16, N18, N19, N25, Q60, Q61, K7, P788, Q44, E10, E11, E12, E13, E14, E24, G1, G2, G3, G4, G5, G6, G7, G8, G9, N083, O24 P700, P701, P702, C, D37, D38, D39, D4, B20, B21, B22, B23, B24, Z94, Z85, D73, Z9621, G960, D561, D578, D571, D570, K900, D70, D71, D72, D76, D80, D81, D82, D83, D84.) Mortality was identified by discharge method 4, and only mortality within 30 d of admission date was considered attributable to the admission (results of the analysis are given in Table S3 in Text S1).

Following this further simplification we obtain a new death model, with only three coefficients to estimate:
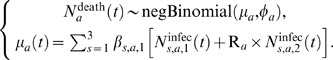
(13)Finally, because the values of 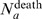, 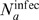, and R*_a_* come with confidence intervals (CIs), we need to account for this uncertainty when computing CFRs. For each age group *a*, we proceed as follows. (1) We generate *K* = 1,000 sample datasets 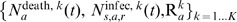, where 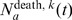 and 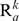 are sampled from a normal distribution with the same mean and 95% CI as in Tables S2 and S3 of Text S1, whereas 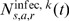 values are obtained by running our model parameters sampled from the joint posterior distribution of our MCMC analysis. (2) We fit the death model for each replicate *k* by maximum likelihood using the function glm.nb of R 2.15.1 [35].

We obtain the distribution of the *K* maximum likelihood estimates 
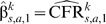
 of Figure S2 in Text S1. These distributions are used to account for uncertainty when computing the distribution of the cumulated number of influenza deaths 
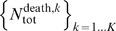
 over the 1995–2009 period predicted under each vaccination scenario:
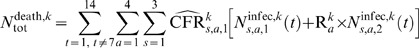
(14)where *t* = 7 corresponds to the 2002/2003 season and is therefore excluded. The 95% credible intervals of the cumulated number of influenza deaths 
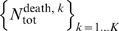
are calculated for each scenario using the 2.5% and 97.5% quantiles of the posterior distributions of the quantities of interest.

### Statistical Inference

The multiple sources of observation give rise to an intractable likelihood, which we explore via data augmentation [36] techniques. In order to manage to integrate numerically the likelihood, we rewrite the likelihood by marginalising some of the augmented variables. Then, we derive a recursive definition of each of the elementary components of the likelihood in order to minimise the number of computational steps involved in the estimation of the likelihood of the model. Finally, we modify the summation surface of the likelihood to derive an accurate approximation of this likelihood by truncating some of the terms with very low probability (see Text S1 for more details of the statistical inference). This allows us to accelerate the computation of the likelihood in order to run 11-million-length chains for each strain and season (1 million for the burn-in). Samples of size 1,000 are obtained by thinning the chain by 10,000.

Serology data were available for the H3N2-dominated 2003/2004 season and facilitated inference for the transmissibility and ascertainment probabilities during this season. The transmissibility between an infectious individual and a susceptible contact is assumed to be constant irrespective of age.

For the remaining 41 subtype-seasons, the estimates of the transmissibility and ascertainment probabilities derived from the 2004/04 H3N2 season with serology were used as priors, allowing us to infer the age-specific susceptibility profiles at the beginning of each of the 41 subtype-seasons.

## Results

**Figure 3:**
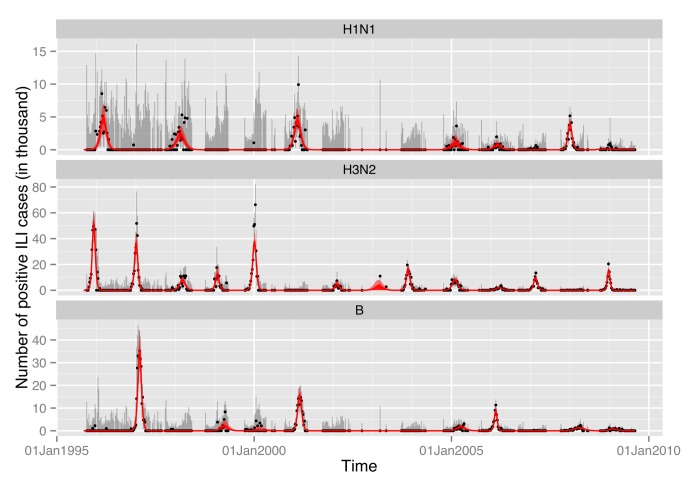
Reconstructed epidemics for the three seasonal subtypes between September 1995 and September 2009. The fit of the model is compared to the age-specific time series of positive ILI cases estimated from the data. For the model, the mean (red line) with 95% CIs (shaded areas) is based on the associated binomial process. For the data, we have represented the unbiased estimator (black dots), with the 95% CI based on a hypergeometric distribution (see Text S1).

**Figure 4:**
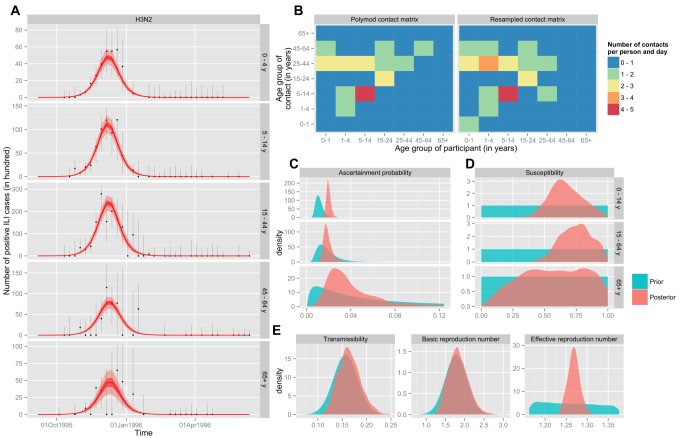
Inference results for H3N2 during the 1995/1996 season. (A) Comparison of the fit of the model to the age-specific time series of positive ILI cases estimated from the data. For the model, the mean (red line) with 50% and 95% CIs (light and dark shaded areas, respectively) is based on the associated binomial process. For the data, we have represented the unbiased estimator (black dots), with the 95% CI associated with the hypergeometric distribution (error bars). (B) Comparison of the contact matrix of the POLYMOD study (left panel) to the resampled matrix of the maximum likelihood MCMC sample (right panel). (C) Age-specific probability of being recorded as ILI and positive if tested and infected. (D) Age-specific susceptibility at the beginning of the flu season. (E) Transmission coefficient (*q*, left panel) and derived quantities: basic (*R*_0_, middle panel) and effective (Re(*t* = 0), right panel) reproduction numbers. For (C–E): the prior distribution is shown in blue, and the posterior distribution in red.

The fitted epidemics manage to reproduce the detailed epidemiological patterns observed in the surveillance data (Figures 3 and 4; Text S1). Reconstructed epidemics by strain reveal the domination of A/H3N2 strains driving influenza epidemiology in the period before the 2009 pandemic. Epidemics from A/H3N2 strains were both more frequent and larger, while B strains tended to result in larger epidemics every few years, and H1N1 remained occasional but smaller in scale. The model is able to capture the epidemiological patterns, and infer key parameters and quantities for each strain in each season of the period. An example of key parameters and quantities inferred from the model is given in Figure 4 (the remaining strains/seasons can be seen in Figures S10–S51 of Text S1). For instance, susceptibility is generally lower in adults than in children (although this is not always the case), and the posterior of the contact matrix is usually relatively close to that observed in a large-scale contact survey [16].

We found the probability of transmission between a susceptible and an infectious person to be 0.17 (0.13–0.22). The ascertainment probability (probability that an infected person will be recorded as having ILI (given the propensity to consult) and have virologically confirmed influenza (assuming that all who consult are swabbed) is 0.0106 (95% credible interval 0.0071–0.0193) among children (15 y), 0.0064 (0.0053–0.0154) among adults (15–64 y), and 0.0139 (0.0063–0.0436) among individuals 65 y or older.

**Figure 5:**
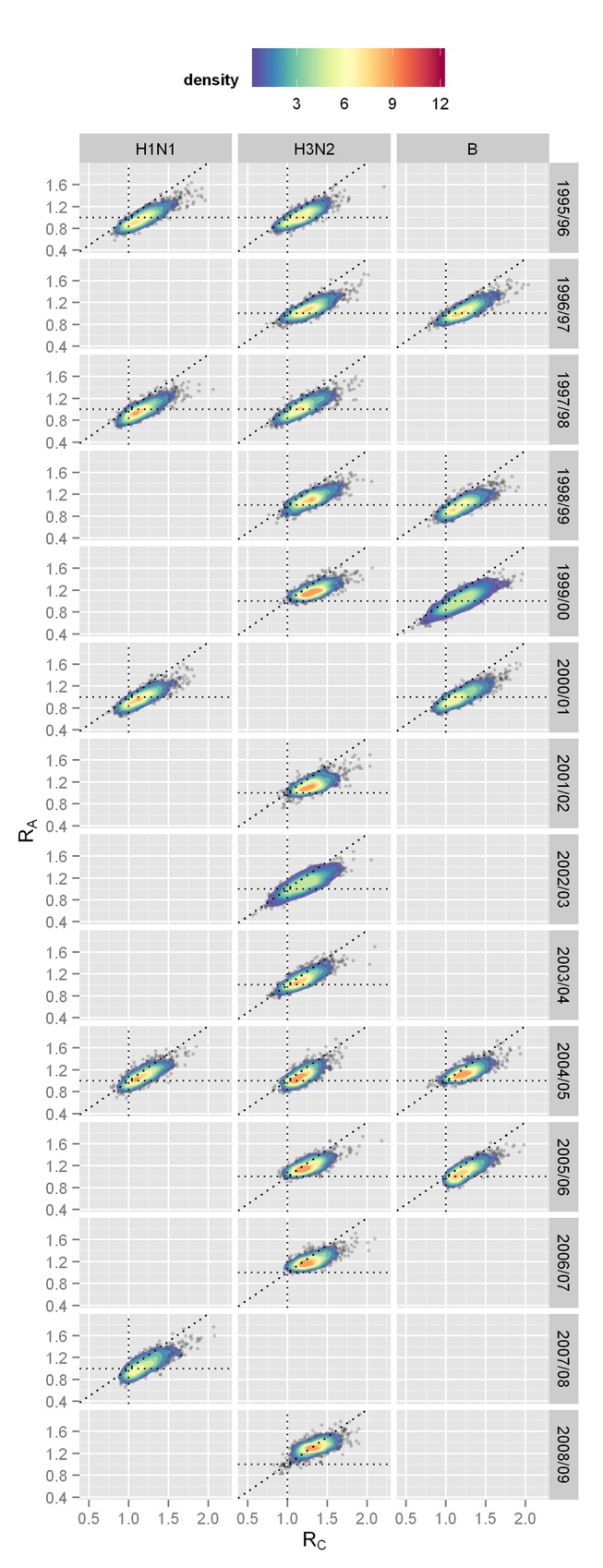
Values (posterior distributions) of the specific reproductive numbers for children (*R*_C_) and adults (*R*_A_) during the study period for all strains present at an epidemic level during the season. Epidemics are defined as including at least 1*R*_C_ and *R*_A_ = 1, respectively. The diagonal dotted line is *R*_C_ = *R*_A_.

We found that in the years where an epidemic occurred, the specific reproductive numbers for children under 15 y and adults (respectively, *R*_C_ and *R*_A_) were equal to 1.2 (95% credible interval 0.9–1.6) and 1.1 (0.8–1.4), respectively. The degree of assortativity *d_R_* was estimated to be 0.3 (0.2–0.4). These specific reproductive numbers vary by subtype, with *R*_A_ being smallest and very close to 1 for H1N1 (see Table 4). With the exception of H3N2 in two seasons, *R*_C_ was consistently greater than *R*_A_ for the remaining 21 strain-specific seasonal outbreaks (Figure 5). Both age groups thus contribute to transmission, with children being the key spreaders.

**Table 4:** Values for the specific reproductive numbers for children under 15(respectively, *R*_C_ and *R*_A_) and the degree of assortativity *d_R_* for the three strains circulating averaged over all seasons for which estimates are possible (i.e., for seasons where a significant epidemic occurred).

Quantity of Interest	Strain		
	H1N1	H3N2	B
*R*_C_	1.2 [0.9–1.6]	1.3 [0.9–1.7]	1.2 [0.9–1.7]
*R*_A_	1.0 [0.8–1.4]	1.1 [0.8–1.5]	1.1 [0.8–1.4]
*d_R_*	0.3 [0.2–0.4]	0.3 [0.2–0.4]	0.3 [0.2–0.4]

**Figure 6:**
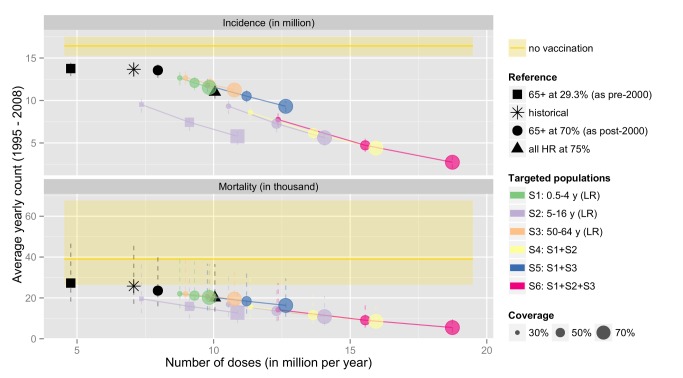
Estimated number of influenza cases and deaths occurring over the 14-y period under the actual vaccination programme, and a series of alternatives. The horizontal axis gives the size (number of doses given) of each of the alternative vaccination programmes (S1 to S6—defined as incremental on the current programme). HR and LR refer to the high-risk and low-risk groups, respectively. The black square represents the estimate of what would have happened if England and Wales had maintained its risk-group-specific vaccination programme throughout the period. The black circle represents what would have happened if the post-2000 programme (targeting vaccination to high-risk and elderly individuals) had been in place throughout the period. The actual vaccination programme is given by the asterisk. Coloured circles represent additions to the current strategy (i.e., extending vaccination to low-risk non-elderly individuals), and coloured squares represent alternative extensions to the pre-2000 programme (i.e., if instead of extending vaccination to low-risk elderly individuals, vaccination had been offered to low-risk individuals in other age groups). The size of the coloured circles and squares represents the assumed coverage achieved, and the different colours represent which age groups are targeted.

Table 5 and Figure 6 show the estimated number of influenza cases and deaths occurring over the 14-y period, under the actual vaccination programme, and a series of alternative scenarios (including no vaccination, which is given by the yellow line in Figure 6). The horizontal axis on Figure 6 gives the number of doses administered under each of the alternative vaccination programmes. The actual vaccination programme is given by the asterisk. Coloured circles represent additions to the current strategy (i.e., extending vaccination to low-risk non-elderly individuals), and coloured squares represent alternative extensions to the pre-2000 programme (i.e., if instead of extending vaccination to low-risk elderly individuals, vaccination had been offered to low-risk individuals in other age groups). If the pre-2000 risk-based programme had remained in place, then an estimated 179 (95% credible interval 168–191) million cases would have occurred, with 338,000 (95% credible interval 221,000–602,000) influenza-related deaths. The expanded programme that included vaccination targeted at all 65+-y-olds post-2000 is estimated to have saved around an additional 18,000 influenza-related deaths, though it reduced incidence only marginally (see Table 5). If, instead, the policy had changed to vaccination of school children (at 30% coverage), this would have significantly reduced both cases and deaths, for about the same number of doses as were in fact used (purple squares in Figure 6). Increasing coverage would reduce both cases and deaths further, but at higher cost in terms of doses used. The optimal choice among policy options (coloured circles) is clear: vaccination of school children is the most efficient strategy, particularly at reducing incidence, followed by the combination strategies, which all involve childhood vaccination.

**Table 5:** Effectiveness of vaccination scenarios.

Baseline^a^	Targeted Populations	Coverage	Doses (in Millions)	Incidence (in Millions per Year)		Mortality (in Thousands per Year)		Infections Averted (per Dose)		Deaths Averted (per 1,000 Doses)	
2	S2: 5–16 y (LR)	15%	6.0	11.5	[10.6–12.4]	22.1	[14.3–40.9]	0.81	[0.75–0.88]	2.46	[1.60–4.30]
2	S2: 5–16 y (LR)	30%	7.4	9.5	[8.6–10.5]	18.8	[11.8–35.0]	0.93	[0.86–1.00]	2.45	[1.61–4.30]
4	S3: 50–64 y (LR)	15%	8.3	13.2	[12.4–14.1]	21.8	[14.3–39.4]	0.38	[0.33–0.44]	1.83	[1.19–3.23]
4	S1: 0.5–4 y (LR)	15%	8.4	13.1	[12.2–13.9]	21.6	[14.2–39.2]	0.40	[0.35–0.45]	1.83	[1.18–3.20]
4	S5: S1+S3	15%	8.7	12.8	[11.9–13.6]	21.1	[13.8–38.3]	0.42	[0.37–0.47]	1.82	[1.18–3.20]
4	S1: 0.5–4 y (LR)	30%	8.8	12.6	[11.8–13.5]	21.0	[13.8–38.1]	0.43	[0.38–0.48]	1.82	[1.18–3.18]
4	S3: 50–64 y (LR)	30%	9.0	12.7	[11.9–13.5]	20.9	[13.6–37.6]	0.42	[0.36–0.47]	1.79	[1.17–3.18]
2	S2: 5–16 y (LR)	50%	9.1	7.4	[6.4–8.5]	15.2	[9.4–28.4]	0.98	[0.90–1.07]	2.41	[1.61–4.12]
4	S2: 5–16 y (LR)	15%	9.2	11.3	[10.4–12.2]	19.0	[12.4–35.0]	0.55	[0.51–0.60]	1.96	[1.27–3.42]
4	S1: 0.5–4 y (LR)	50%	9.3	12.1	[11.2–12.9]	20.2	[13.2–36.6]	0.47	[0.42–0.52]	1.82	[1.17–3.19]
4	S4: S1+S2	15%	9.6	10.9	[10.0–11.8]	18.3	[11.9–33.8]	0.58	[0.53–0.63]	1.95	[1.27–3.41]
4	S5: S1+S3	30%	9.8	11.8	[11.0–12.6]	19.5	[12.8–35.5]	0.47	[0.42–0.52]	1.78	[1.16–3.13]
4	S1: 0.5–4 y (LR)	70%	9.8	11.5	[10.7–12.4]	19.3	[12.7–34.9]	0.50	[0.45–0.55]	1.81	[1.17–3.19]
4	S3: 50–64 y (LR)	50%	9.9	11.9	[11.2–12.7]	19.7	[12.8–35.3]	0.45	[0.40–0.51]	1.75	[1.14–3.12]
4	S6: S1+S2+S3	15%	10.0	10.6	[9.7–11.4]	17.8	[11.5–32.8]	0.58	[0.54–0.63]	1.92	[1.25–3.34]
4	S2: 5–16 y (LR)	30%	10.5	9.3	[8.4–10.2]	16.0	[10.1–30.1]	0.67	[0.62–0.73]	1.99	[1.31–3.46]
4	S3: 50–64 y (LR)	70%	10.8	11.2	[10.5–11.9]	18.5	[12.0–33.1]	0.48	[0.42–0.54]	1.72	[1.12–3.07]
2	S2: 5–16 y (LR)	70%	10.9	5.8	[4.8–6.8]	12.2	[7.4–23.3]	0.97	[0.89–1.06]	2.27	[1.53–3.93]
4	S5: S1+S3	50%	11.2	10.5	[9.8–11.2]	17.5	[11.5–31.7]	0.53	[0.48–0.58]	1.74	[1.14–3.07]
4	S4: S1+S2	30%	11.3	8.6	[7.6–9.5]	14.9	[9.4–28.0]	0.69	[0.64–0.75]	1.95	[1.29–3.40]
4	S2: 5–16 y (LR)	50%	12.3	7.3	[6.2–8.3]	12.9	[8.0–24.3]	0.74	[0.68–0.80]	1.96	[1.31–3.40]
4	S6: S1+S2+S3	30%	12.4	7.8	[6.9–8.6]	13.5	[8.4–25.8]	0.70	[0.65–0.75]	1.90	[1.25–3.30]
4	S5: S1+S3	70%	12.6	9.3	[8.7–10.0]	15.5	[10.1–28.4]	0.56	[0.51–0.62]	1.7	[1.11–3.01]
4	S4: S1+S2	50%	13.6	6.2	[5.2–7.1]	11.2	[6.9–21.2]	0.75	[0.69–0.82]	1.91	[1.26–3.32]
4	S2: 5–16 y (LR)	70%	14.1	5.6	[4.6–6.6]	10.4	[6.3–20.0]	0.76	[0.70–0.83]	1.90	[1.27–3.26]
4	S6: S1+S2+S3	50%	15.6	4.7	[3.9–5.5]	8.8	[5.2–17.2]	0.75	[0.69–0.81]	1.83	[1.21–3.16]
4	S4: S1+S2	70%	15.9	4.4	[3.4–5.3]	8.3	[4.8–16.1]	0.76	[0.69–0.82]	1.82	[1.20–3.16]
4	S6: S1+S2+S3	70%	18.7	2.7	[2.0–3.4]	5.3	[2.9–10.9]	0.73	[0.68–0.79]	1.70	[1.13–2.98]

Each scenario increments on a baseline strategy (numbers refer to Table S7 in Text S1). Numbers inside brackets indicate 95% credible intervals.

a Baseline 2 refers to vaccination as it occurred before 2000 (risk-based only), and baseline 4 is the post-2000 policy (high-risk group and elderly adult group). For more detail see Text S1.

The historical programme is estimated to have prevented 0.39 (95% credible interval 0.34–0.45) infections for each dose given and 1.74 (95% credible interval 1.16–3.02) deaths for every 1,000 doses. Extending the current programme to the low-risk age group most at risk (50–64 y) would improve the overall efficiency of the programme (0.43 [95% credible interval 0.35–0.52] infections prevented for each dose), but deaths prevented would remain at 1.77 (95% credible interval 1.15–3.14) for 1,000 doses. In comparison, a policy extending vaccination to the main transmitters (children aged 5–14 y) would prevent 0.70 (95% credible interval 0.52–0.81) infections per dose and 1.95 (95% credible interval 1.28–3.39) deaths per 1,000 doses given.

**Figure 7:**
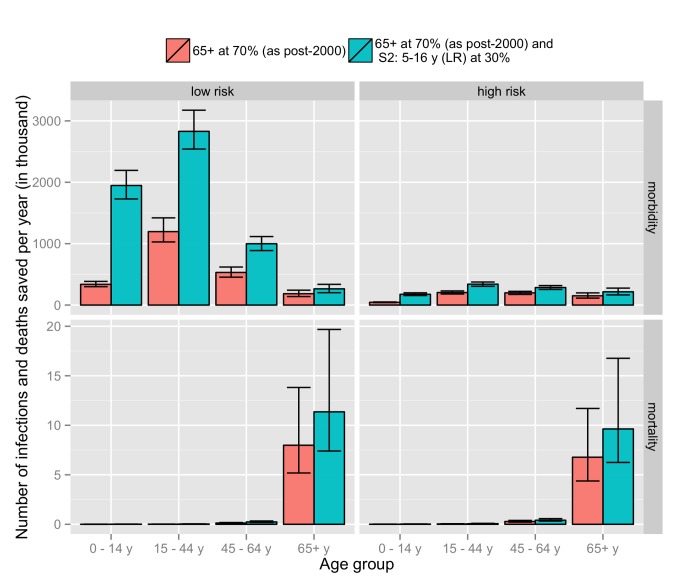
Comparison of the number of cases saved per year for the current strategy (vaccination of high-risk and 65+-y individuals) with an extension of the current strategy that additionally targets the 5–16-y age group. Results are shown for number of infections (morbidity) and deaths (mortality) by age and risk group.

Figure 7 shows the distribution by age and risk group of infections and deaths averted for two of the evaluated strategies (current and the extension to children 5–16 y old). Most of the additional benefit in terms of infection is in the vaccinated age group (low-risk 5–16-y-olds) but with a significant impact in young adults (17–44-y-olds). With regards to mortality, the vast majority of deaths averted are among low- and high-risk elderly adults (65+-y-olds).

## Discussion

Most high-income countries vaccinate high-risk individuals and/or elderly adults against influenza. The necessity to vaccinate annually represents a major financial and logistical challenge. This is especially so in temperate countries where, because of the seasonality of influenza, vaccines need to be given during a short time window. Judging whether the existing programmes represent a good use of these resources, and whether the programmes should be expanded (or indeed reduced) is, however, far from straightforward. Individually randomised controlled trials and cohort studies give information only on individual protection, not on the overall effect of the intervention in the population. Cluster randomised trials can estimate the overall effect [37], but are expensive to conduct and are therefore rare. Before and after studies are difficult to interpret given the variability in influenza and vaccine match from season to season. All of these experimental or observational studies provide no information on alternative strategies (i.e., strategies not directly observed in the study). Here we use a mathematical model in conjunction with a number of detailed datasets collected over multiple seasons to fill help this void.

The close fit of the model to the totality of the data and the extensive simulation and analytical work undertaken to establish the performance of the statistical approaches employed (see Text S1) give confidence in the models ability to accurately reproduce a series of counterfactual histories: in other words, the models ability to predict what would have happened if an alternative vaccination regime had been in place. While the vaccination of the population at highest risk did significantly reduce mortality, it had little effect on transmission. It appears that a vaccination programme based on targeting the main transmitters, i.e., children, is the most efficient at reducing not only infection but also mortality, which disproportionately affects older individuals and those with high-risk conditions. Indeed, the results even suggest that the change to targeting all individuals 65 y and older that occurred in 2000 in the UK was not the best strategy. Targeting children would likely have prevented more cases and deaths for similar numbers of doses, even if low levels of coverage (30%) had been achieved. Such results have clear implications for similar countries considering their influenza vaccination policies. It also shows that in countries, such as the US, that have introduced a childhood programme, albeit at relatively low coverage [38], substantial benefits to children and others in the community can still occur. Improving coverage in children should be a priority as this is likely to bring the greatest benefit to the community as a whole.

This work is based on modern developments of Bayesian statistics allowing us to break down the influenza transmission into biological (immunity, probability of transmission of the virus), social (contact pattern), and healthcare (ascertainment probabilities) parameters. This method is based on data largely available in many other countries. It integrates a novel method to estimate relevant contact patterns. Comparison of the posteriors of the model with the prior shows that the use of contact survey data such as from the POLYMOD study gives a reasonable description of contacts involved in influenza transmission.

Some of the estimates (particularly the number of deaths) have wide credible intervals. This is due to the uncertainty regarding the estimation of epidemiological parameters for influenza and difficulties in estimating the CFRs for the different age groups. This leads to some of the estimates for the different strategies having wide and overlapping credible intervals. However, these estimates are not independent since they are generated from simulations with the same epidemiological parameters and CFRs but different vaccination scenarios. Therefore, one must focus upon the estimate of the difference between two scenarios in order to test for statistical significance (or lack thereof). In fact, when one considers pairs of scenarios, the difference between each of the two scenarios is typically statistically significant. This is not apparent when inspecting the marginals because of the correlation structure. For example, when compared to the non-vaccination strategy, the median number of deaths averted per 1,000 doses is greater for strategy S2 than for strategy S3 (1.99 versus 1.79 incremental on the post-2000 programme and 30% coverage; see Table 2), but they show overlapping 95% credible intervals (1.31–3.46 versus 1.17–3.18). However, when strategy S2 is pairwise compared with strategy S3, we find that strategy S2 averts a median of 0.82 more deaths than strategy S3 per 1,000 doses (95% credible interval 0.53–1.48). In Figures S58 and S59 of Text S1, we show a computation of the *p*-values of the differences in terms of cases and deaths averted per dose for each of the possible pairs of scenarios from the study.

We have used only serological data tested against one A/H3N2 virus during one season. This has been used as an informative prior for the other strains and seasons. Further serological analyses are needed to inform the differences in transmissibility of influenza viruses in human populations. This study provides the methodology to do so using largely available surveillance data combined with serological surveys. Another assumption is that the propensity to consult with a GP for flu-like symptoms is constant throughout the year. Further studies involving sequential serology would help in providing insight into this parameter.

The pattern inferred by the ascertainment probability (linked with the propensity to consult per infection) reveals a V-shape curve, where ascertainment probability is higher in children and elderly adults and twice as low in adults under 65 y. The ascertainment probability can be interpreted as the number of confirmed cases resulting if everybody recorded as ILI were virologically tested. This number is of the order of magnitude of 0.5%–1%, revealing the difficulty of understanding the underlying transmission dynamics through a clinical surveillance system because of the different “filters” (symptoms, consultation, assessment of the GP, sensitivity of the test, etc.).

The calculation of the specific reproductive numbers for children and adults alongside the degree of assortativity of the effective contacts reveals a key characteristic of the epidemic: the transmission is mainly driven by children, with the benefits of vaccination greatest in this age group. Nevertheless, the degree of assortativity of 0.3 (0 representing no contacts between children and adults and 1 representing homogeneous mixing) indicates that intervention in children is likely to provide strong herd immunity for the remaining population. It should also be noted that in this paper we have adopted a two-group representation of the dynamics for ease of visualising the respective role of children and adults. In the full model, we use seven age groups.

In our model, each season and each strain circulating within that season is modelled in isolation. However, there should be a link between strain-specific immunity at the beginning of a season, number of infections during that season, and immunity at the beginning of the following season. The annual change in strain-specific immunity is complicated by the fact that influenza strains are drifting, generating variant viruses escaping the population immunity. The integration of a mechanism for the building and propagation of immunity (including interaction with the immune history of the age group in the population) would help improve understanding of the evolutionary dynamics of the virus and its interaction with immunity at a population level. It should also allow better estimates of the epidemic parameters for each season. Propagation of immunity could also have an impact on the overall efficiency of the vaccine programme. Annual re-vaccination could increase the overall programme benefit by providing protection for longer than one season. However, by reducing natural infections in unvaccinated individuals, it might allow pools of susceptible individuals to build up in particular parts of the population. This is an area of future research.

The vaccination model developed is based on a series of simplifying assumptions. First, protection from vaccination is considered as absolute. Alternatives about the modelling of the impact of vaccination exist. For example, vaccination can reduce susceptibility or transmission. The vaccine efficacy is set to be 70% and 42% for the individuals under 65 y and 44% and 28% for individuals aged 65+ y during, respectively, well-matched years and non-well-matched years, i.e., when the strain present in the vaccine and the strain circulating differ. These values are based of the available data for some of the existing vaccines; they are likely to vary with the type of vaccine used (live attenuated influenza vaccine or trivalent inactivated vaccine), the season, the age and risk groups, and the subtype targeted.

As we have serological data for one season and subtype only, we use the same informative prior for the transmissibility (*q*) of the virus for all age groups and subtypes. This is not a problem, in principle, as the posterior distribution of the parameter should reflect for each subtype and season an update combining the other sources of information with this prior. Nevertheless, because of the identifiability problem with the background immunity, the transmissibility prior is strongly informative and will drive the shape of the posterior. More studies would be needed to evaluate the respective transmissibility of the different circulating subtypes, and possible changes from one season to the other.

The value of the ascertainment probability (ε*_i_*) is assumed in our study to be constant over the course of the season. The underlying assumption is that the quantities contributing to the ε*_i_* remain constant over time. One of them, the propensity to consult with a GP if symptomatic, is likely to vary during the season. Though there is little evidence of large variation of this number for seasonal influenza, it has been shown that this number could vary in particular circumstances such as a pandemic where media coverage changes the perception of the disease among the population where the epidemic is occurring.

To be able to translate the number of infections into expected deaths, we used estimates from another published study on the number of deaths from influenza during a subset of the seasons we analysed. Ideally this number of deaths should be estimated inside the Bayesian inference framework. Unfortunately, deaths are difficult to attribute as the link with a particular pathogen is rarely identified. Thus, we would need to use a non-specific measure of deaths (such as all-cause mortality or acute respiratory illness), and then attribute deaths to influenza, taking account of other potential causes of death. Methods integrating time series from other pathogens could be inserted in the current model to provide such estimates. This work paves the way towards further developments in that direction.

Traditional vaccination strategies against influenza have targeted those most at risk of serious consequences of infection. This comprehensive modelling study, which builds on detailed strain and age- and risk-group-specific data, suggests that with the current level of immunisation in high-income countries, additions to the current risk/age-based strategy should now be considered. The most efficient way of reducing overall influenza-attributable morbidity and mortality appears to be to target the key spreaders—children. This strategy exploits the low reproduction number of the influenza virus and its dependence on spread from children who have higher levels of susceptibility and higher contact rates. Targeting at-risk individuals and elderly adults offers some protection to those immunised, but little to others in the population. Adoption of more innovative strategies that aim to block transmission (in addition to targeting those most at risk) should be more widely adopted. Even with modest coverage, substantial further reductions in morbidity and mortality could be achieved.

## Supporting Information

Text S1 **Supplementary information.**
(PDF)

## Abbreviations

CFR: case fatality ratio
CI: confidence interval
GP: general practitioner
HPA/DH: Health Protection Agency/Department of Health
ILI: influenza-like-illness
MCMC: Markov chain Monte Carlo
RCGP: Royal College of General Practitioners
