# Supplementary material for: Assessing Optimal Target Populations for Influenza Vaccination Programmes: An Evidence Synthesis and Modelling Study
Source: PLoS Med. 2013 Oct 8;10(10):e1001527. doi: 10.1371/journal.pmed.1001527 (PMC3793005; doi:10.1371/journal.pmed.1001527)
Supplement: Text S1 — Supplementary information. (PDF) [file pmed.1001527.s001.pdf]

Supplementary information for

Assessing changes to influenza vaccination programmes: an evidence synthesis and modelling study

M. Baguelin[1,2,*], S. Flasche[1,2,3], A. Camacho[1,2], N. Demiris[4], E.Miller[1] and W.J. Edmunds[2,1]

1 Immunisation, Hepatitis and Blood Safety Department, Health Protection Agency, London, United Kingdom
2 Centre for the Mathematical Modelling of Infectious Diseases, London School of Hygiene and Tropical Medicine, London, United Kingdom
3 Department of Mathematics and Statistics, University of Strathclyde, United Kingdom
4 Department of Statistics, Athens University of Economics and Business, Athens, Greece

[*]*Corresponding author*:
E-mail: marc.baguelin@hpa.org.uk

Contents

1 Introduction 2

2 Additional methods 2
2.1 Details of the modelling approach . . . 2
2.1.1 Diagram and coverage for the vaccination model . . . 2
2.1.2 Equations of the epidemiological model . . . 2
2.1.3 Description of the observation model using sets . . . 3
2.1.4 Reproduction numbers and mixing between groups . . . 5
2.1.5 Estimation of the number of deaths due to influenza . . . 6
2.2 Statistical inference . . . 6
2.2.1 Inference of the contacts . . . 6
2.2.2 Identifiability of the variables . . . 8
2.2.3 Derivation of the priors . . . 8
2.2.4 Derivation of the likelihood . . . 10
2.3 Implementation . . . 12
2.3.1 Implementation of the epidemic model . . . 12
2.3.2 Proposal for the contact matrix . . . 12
2.3.3 Implementation of the likelihood . . . 12
2.3.4 Implementation of the Markov chain Monte Carlo . . . 17
2.4 Comparison of our statistical framework with other approaches . . . 21

3 Supplementary results 24
3.1 Inference for 14 seasons and 3 subtypes . . . 24
3.1.1 Season 2003/04 with serology . . . 24
3.1.2 Other seasons . . . 24
3.1.3 Strain: H1N1 . . . 24
3.1.4 Strain: H3N2 . . . 33
3.1.5 Strain: B . . . 40
3.2 Detailed time series by subtype and age group . . . 47
3.3 Posteriors of the contact matrices . . . 47
3.4 Assortativity and reproduction numbers . . . 47
3.5 Pairwise significance of differences between strategies . . . 48
3.6 Sensitivity analysis . . . 48

3.6.1 Prior on transmissibility 48
3.6.2 Improved vaccine efficacy in elderly 49

Reference numbering

In order to get a common referencing between the main paper and the supplementary information, we have defined an unique list of references for the bibliography. References [1–39] thus are classified by order of appearance in the main manuscript. References [40–46] are only present in the supplementary information and are ordered following their first appearance in the supplementary information.

1 Introduction

This supplementary information report aims at complementing the main paper by describing in more details some of the more technical points of the methods. Section 2.1 presents complementary details about the structure of the model. Section 2.2 is dedicated to the statistical inference of the parameters from the model. Section 2.3 makes explicit the algorithms used for the implementation of the model and finally Section 2.4 reviews and discusses other existing methods. Section 3, the last part of this report, presents additional results that were not included in the main text.

2 Additional methods

2.1 Details of the modelling approach

2.1.1 Diagram and coverage for the vaccination model

Figure S1 represents a diagram of the vaccination model, while in Table S1 the final coverage by age, risk group and season used in the model can be found.

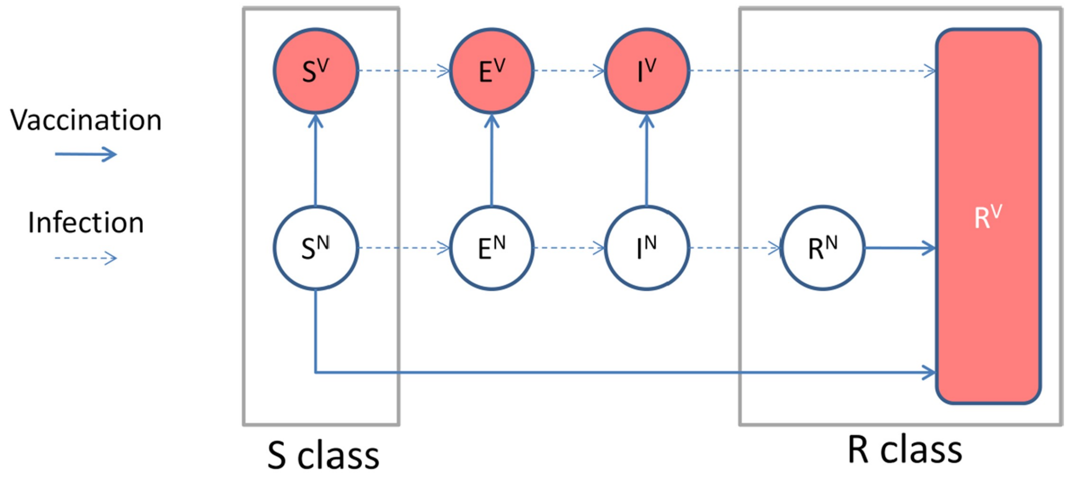

Figure S1: Diagram of the vaccination mode based on a SEIR model with vaccinated (V) and non-vaccinated (N) statuses

2.1.2 Equations of the epidemiological model

The equations of the epidemiological model are as follows (in which time dependencies have been dropped for clarity):

Risk Group	Low							High						
Age group	<1	1-4	5-14	15-24	25-44	45-64	65+	<1	1-4	5-14	15-24	25-44	45-64	65+
1995/96	0.06	0.11	0.44	1.00	1.75	6.30	28.37	0.56	1.11	3.46	7.10	11.55	27.15	52.20
1996/97	0.06	0.11	0.42	0.95	1.60	5.95	27.94	0.83	1.67	3.74	6.55	11.00	26.50	51.14
1997/98	0.06	0.11	0.38	1.10	1.90	7.03	32.10	0.94	1.89	4.04	7.30	12.50	29.93	56.95
1998/99	0.06	0.11	0.45	0.90	1.65	6.35	27.70	0.89	1.78	3.77	6.30	11.65	28.77	50.72
1999/00	0.06	0.11	0.36	0.85	1.55	6.55	30.60	1.33	2.67	4.13	6.45	11.75	29.32	53.98
2000/01	0.06	0.11	0.35	0.90	1.75	7.75	65.80	1.50	3.00	4.80	7.50	14.20	35.38	65.80
2001/02	0.11	0.22	0.46	0.85	1.70	7.65	67.50	1.39	2.78	4.71	7.80	14.75	36.55	67.50
2002/03	0.06	0.11	0.44	0.85	1.65	7.80	69.40	1.44	2.89	4.62	7.50	14.85	37.73	69.40
2003/04	0.17	0.33	0.55	0.90	1.80	8.22	71.55	3.39	6.78	7.70	9.55	17.60	41.73	71.55
2004/05	0.11	0.22	0.48	0.87	1.72	7.89	71.40	2.73	16.10	19.64	41.65	42.81	42.81	71.40
2005/06	0.11	0.22	0.48	0.87	1.72	7.89	75.30	3.28	19.36	23.63	50.10	51.50	51.50	75.30
2006/07	0.11	0.22	0.48	0.87	1.72	7.89	73.90	2.88	16.98	20.72	43.94	45.17	45.17	73.90
2007/08	0.11	0.22	0.48	0.87	1.72	7.89	73.50	3.10	18.27	22.30	47.28	48.60	48.60	73.50
2008/09	0.11	0.22	0.48	0.87	1.72	7.89	73.50	3.10	18.27	22.30	47.28	48.60	48.60	73.50

Table S1: Final coverage by age, risk group and season used in the model. For the 0-1 age-group, only infants over 6 months receive vaccination.

$$\left\{\begin{array}{lllllll}
\frac{dS_{ik}^N}{dt} & = & -\lambda_i S_{ik}^N - \mu_{ik} S_{ik}^N & ; & \frac{dS_{ik}^V}{dt} & = & -\lambda_i S_{ik}^V + (1-\alpha_i)\mu_{ik} S_{ik}^N \\
\frac{dE_{ik}^{1N}}{dt} & = & \lambda_i S_{ik}^N - \gamma_1 E_{ik}^{1N} - \mu_{ik} E_{ik}^{1N} & ; & \frac{dE_{ik}^{1V}}{dt} & = & \lambda_i S_{ik}^V - \gamma_1 E_{ik}^{1V} + \mu_{ik} E_{ik}^{1N} \\
\frac{dE_{ik}^{2N}}{dt} & = & \gamma_1 (E_{ik}^{1N} - E_{ik}^{2N}) - \mu_{ik} E_{ik}^{2N} & ; & \frac{dE_{ik}^{2V}}{dt} & = & \gamma_1 (E_{ik}^{1V} - E_{ik}^{2V}) + \mu_{ik} E_{ik}^{2N} \\
\frac{dI_{ik}^{1N}}{dt} & = & \gamma_1 E_{ik}^{2N} - \gamma_2 I_{ik}^{1N} - \mu_{ik} I_{ik}^{1N} & ; & \frac{dI_{ik}^{1V}}{dt} & = & \gamma_1 E_{ik}^{2V} - \gamma_2 I_{ik}^{1V} + \mu_{ik} I_{ik}^{1N} \\
\frac{dI_{ik}^{2N}}{dt} & = & \gamma_2 (I_{ik}^{1N} - I_{ik}^{2N}) - \mu_{ik} I_{ik}^{2N} & ; & \frac{dI_{ik}^{2V}}{dt} & = & \gamma_2 (I_{ik}^{1V} - I_{ik}^{2V}) + \mu_{ik} I_{ik}^{2N} \\
\frac{dR_{ik}^N}{dt} & = & \gamma_2 I_{ik}^{2N} - \mu_{ik} R_{ik}^N & ; & \frac{dR_{ik}^V}{dt} & = & \gamma_2 I_{ik}^{2V} + \mu_{ik}(R_{ik}^N + \alpha_i S_{ik}^N)
\end{array}\right. \tag{1}$$

where S_{ik}^N and S_{ik}^V represent the number of susceptibles of age group i and risk group k with different vaccine history. The susceptibles indexed by N are naive and the ones indexed by V have received the vaccine. E_{ik}^{1X} and E_{ik}^{2X} represent exposed, but not yet infectious individuals with vaccine history X. I_{ij}^{1X} and I_{ij}^{2X} represent infectious individuals. R_{ik}^X represent immune individuals of age class i and risk group k with vaccine history X, and λ_i is the age-group specific force of infection, given by:

$$\lambda_i = q\sigma_i \sum_{j=1}^{7} \sum_{k=1}^{2} \sum_{X=\{N,V\}} c_{ij}(I_{jk}^{1X} + I_{jk}^{2X}) \tag{2}$$

where q is the transmissibility parameter, c_{ij} is the rate at which individuals in age group i make contact with those in age group j and σ_i is the susceptibility of age group i; $\mu_{ik} = \frac{\nu_{ik}}{S_{ik}^N + E_{ik}^{1N} + E_{ik}^{2N} + I_{ik}^{1N} + I_{ik}^{2N} + R_{ik}^N}$ with ν_{ik} the rate of immunization of risk group k in age group i.

2.1.3 Description of the observation model using sets

We would like to stress here that the ascertainment probability ϵ_i should not be confused with the propensity to consult among infected people. To illustrate the differences between the two, we can use a set notation to write down the different states (which refers to the same subtype) to which an individual in age group i can belong at week j (illustrated in Figure 2 of the main paper):

- A: be infected by one given subtype of influenza;
- B: have an influenza infection detectable[1] by the virological test as applied in field condition;
- C: develop ILI symptoms;
- D: go to the GP;
- E: be recorded as having ILI;

[1]The ability to detect influenza viruses in swabs with the virological test will depend on a certain number of factors, such as the time between sampling and infection, it does not represent the sensitivity of the test in ideal conditions but rather in 'typical' field conditions.

- F: be tested virologically as part of the ILI testing scheme.

It is then possible to describe derived quantities using the "conditional" and "intersection" set operators noted respectively | and $\cap$. These operators have both a logical and probabilistic interpretation[2]. $A \cap D$ can be read as "A and D" i.e. if event A and D are true and corresponds thus to the combined event of being infected by a specific subtype of influenza and going to the GP. Similarly, $P(B|A)$ refers to the probability of event B conditional on event A, so $P(B|A)$ is the probability to be positive to a specific subtype (event B) given than the individual has been infected by this subtype (event A). Then, if we drop the age group and week indices for clarity, the model quantities can be written as:

$$\begin{array}{rclrclrcl} m &=& |E|; & m^+ &=& |B \cap E|; & z^{outside} + z^{\theta} &=& |A|; \\ n &=& |F|; & n^+ &=& |B \cap F|; & & & \end{array} \tag{3}$$

and the ascertainment probability ϵ is equal to $P(B \cap E|A)$ while the probability to consult among infected people is $P(D|A)$. Some other common and tangible epidemiological quantities can be formulated using this set notation:

- $P(B|A)$ is the sensitivity of the virological test against influenza cases;
- $P(B|A \cap C)$ is the sensitivity of the virological test against clinical influenza cases;
- $P(C|A)$ is the proportion of symptomatic[3] among influenza cases;
- $P(D|A \cap C)$ is the propensity to consult[4] for clinical influenza infections;
- $P(E|A \cap C \cap D)$ is the GP ability to recognize a patient with influenza symptoms;

We can thus express ϵ, the ascertainment probability, by way of using these quantities. For this we start with, using conditioning, separating the GP and virological surveillance streams:

$$\epsilon = P(B \cap E|A) \tag{4}$$

$$= \underbrace{P(E|A)}_{GP} \underbrace{P(B|A \cap E)}_{virology}. \tag{5}$$

$P(E|A)$ can be further broken down in terms of the elementary processes:

$$\begin{aligned} P(E|A) &= \frac{P(A \cap E)}{P(A)} \\ &= \frac{P(A \cap E)}{P(A \cap C \cap D)} \frac{P(A \cap C \cap D)}{P(A \cap C)} \frac{P(A \cap C)}{P(A)}. \end{aligned}$$

As we assume that $A \cap E = A \cap C \cap E$ (all individuals recorded as ILI consulting following an influenza infection have ILI symptoms) and $E \subset D$, $P(A \cap E) = P(A \cap C \cap D \cap E)$, we have then:

$$\frac{P(A \cap E)}{P(A \cap C \cap D)} = P(E|A \cap C \cap D), \tag{6}$$

i.e. the GP ability to recognize a patient with influenza symptoms. As $A \cap E = A \cap C \cap E$, we can rewrite ϵ as:

$$\epsilon = P(E|A \cap C \cap D)P(D|A \cap C)P(C|A)P(B|A \cap C \cap E) \tag{7}$$

$P(B|A \cap C \cap E)$ is the probability to have developed a (virologically) detectable influenza infection given that the individual is infected and picked up as ILI by the GP. We can assume that B and E are independent conditional on $A \cap C$. That means that the probability that a symptomatic individual who goes to the GP has a detectable influenza is independent of the assessment by the GP to record this patient as ILI[5]. We thus have $P(B \cap E|A \cap C) \approx P(B|A \cap C)P(E|A \cap C)$ and $P(B|A \cap C \cap E) \approx P(B|A \cap C)$.

We can finally write Equation (7) using only elementary epidemiological quantities:

$$\epsilon \approx P(E|A \cap C \cap D)P(D|A \cap C)P(C|A)P(B|A \cap C), \tag{8}$$

where $P(E|A \cap C \cap D)$ is the GP recognition ability, $P(D|A \cap C)$ the propensity to consult among symptomatic influenza cases, $P(C|A)$ the proportion with symptoms among the infected and $P(B|A \cap C)$ the sensitivity of the test against clinical influenza cases.

[2] Both fundamental logic and probability can be theoretically ultimately ground to set theory.

[3] Given the continuous range of symptoms associated with influenza, a case definition is needed for influenza-like symptoms. We match here the definition used for our ILI data, i.e. fever plus at least one other respiratory symptom.

[4] Assuming that the influenza symptoms are the cause of the visit to the GP.

[5] It is possible though that a more severe influenza infection would have a higher chance of being classified as ILI by the GP or result as a positive from the virology testing. Nevertheless, as this potential bias is integrated into the chain of probabilities of the ascertainment probability ϵ, this assumption does not impact on the model developed.

2.1.4 Reproduction numbers and mixing between groups

The epidemiological potential of a pathogen is often described by way of its basic reproduction number R_0, defined as the average number of secondary cases following the introduction of an infectious individual in a totally susceptible population. Mathematically, R_0 is defined as the biggest eigenvalue of the next generation matrix (NGM). When the population is not entirely susceptible (presence of immunity), a similar quantity can be defined, called the effective reproduction number and noted R_e. Nevertheless, these quantities synthesize the dynamical properties of potential outbreaks using a homogeneous mixing paradigm (all individual are equally likely to contact each other) and thus do not capture the dynamics associated with the intra- and inter-group transmission in population stratified in non-homogeneously mixing groups.

In the setting of targeted vaccination campaigns where interruption of transmission is aimed, summary quantities should reflect the potential of transmission of the targeted groups. The requirement of more detail should be balanced with the necessity for summary quantities to be simple to interpret. We thus have chosen to compute in addition to R_0 and R_e, the 2-by-2 NGM matrix reduced to the children (under 15 years) and adults.

The basic-reproduction number R_0, the effective reproduction number R_e, and the 2-by-2 reduced NGM are derived quantities, dependent on other parameter values, rather than fitted directly in our model. R_0 and R_e are obtained from the dominant eigenvalue of the next generation operator, adjusting for susceptibility, as appropriate. For a given mixing matrix and susceptibility profile, R_0 is proportional to q, the estimated transmission coefficient, which is estimated for each strain and each year.

We investigate in the following sections, the link between this 2-by-2 reduced NGM matrix and the potential of transmission in the whole population summarized by R_0.

General formula for the 2-by-2 case The NGM of a population with two age groups C and A can be written[6] as:

$$M_R = \begin{pmatrix} R_{CC} & R_{CA} \\ R_{AC} & R_{AA} \end{pmatrix} \tag{9}$$

The basic reproductive number R_0 is given by the dominant eigenvalue of the NGM. To calculate the eigenvalues, one should find the roots of the characteristic polynomial:

$$P_R(\lambda) = det(M_R - \lambda I) \tag{10}$$

with I the 2x2 identity matrix. We need thus to solve:

$$\begin{aligned} P_R(\lambda) &= 0 \\ (R_{CC} - \lambda)(R_{AA} - \lambda) - R_{AC}R_{CA} &= 0 \\ \lambda^2 - (R_{CC} + R_{AA})\lambda + R_{CC}R_{AA} - R_{AC}R_{CA} &= 0 \\ \left[\lambda - \frac{1}{2}(R_{CC} + R_{AA})\right]^2 - \left[\frac{1}{2}(R_{CC} + R_{AA})\right]^2 + R_{CC}R_{AA} - R_{AC}R_{CA} &= 0 \\ \left[\frac{1}{2}(R_{CC} + R_{AA})\right]^2 + R_{AC}R_{CA} - R_{CC}R_{AA} &= \left[\lambda - \frac{1}{2}(R_{CC} + R_{AA})\right]^2 \end{aligned}$$

so we finally get

$$\lambda = \frac{1}{2}(R_{CC} + R_{AA}) \pm \sqrt{\left[\frac{1}{2}(R_{CC} + R_{AA})\right]^2 + R_{CA}R_{AC} - R_{CC}R_{AA}}$$

and thus, assuming that $R_{CC}R_{AA} \neq 0$, we have:

$$R_0 = \frac{1}{2}(R_{CC} + R_{AA}) + \sqrt{\left[\frac{1}{2}(R_{CC} + R_{AA})\right]^2 + (d_R - 1)R_{CC}R_{AA}} \tag{11}$$

where $d_R = \frac{R_{CA}R_{AC}}{R_{CC}R_{AA}}$ the degree of assortativity of the matrix. We assume that for a disease such as influenza, where transmission is associated with close contact, transmission $R_{CC}R_{AA} \geq R_{CA}R_{AC}$ and thus $0 \leq d_R \leq 1$.

[6]In the main paper, for clarity reasons, R_{CC} and R_{AA} are denoted by R_C and R_A respectively.

Assortative sub-populations If the transmission structure is very assortative then $d_R \approx 0$ and developping Equation (11) with $d_R = 0$ leads to

$$\begin{aligned} R_0 &= \frac{1}{2}(R_{CC} + R_{AA}) + \sqrt{\left[\frac{1}{2}(R_{CC} + R_{AA})\right]^2 - R_{CC}R_{AA}} \\ &= \frac{1}{2}(R_{CC} + R_{AA}) + \sqrt{\left[\frac{1}{2}(R_{CC} - R_{AA})\right]^2} \\ &= \frac{1}{2}(R_{CC} + R_{AA}) + \frac{1}{2}|R_{CC} - R_{AA}| \end{aligned}$$

where $|R_{CC} - R_{AA}| = R_{CC} - R_{AA}$ if $R_{CC} \geq R_{AA}$ and $R_{AA} - R_{CC}$ else.

As a result, we have[7] that for two fully assortative sub-populations:

$$R_0 = \max(R_{CC}, R_{AA}). \tag{12}$$

Highly coupled sub-populations $d_R = 1$

$$\begin{aligned} R_0 &= \frac{1}{2}(R_{CC} + R_{AA}) + \sqrt{\left[\frac{1}{2}(R_{CC} + R_{AA})\right]^2} \\ &= R_{CC} + R_{AA}. \end{aligned} \tag{13}$$

Different epidemiology for the same R_0 As R_0 varies monotonously when d_R varies between 0 and 1, we have:

$$\max(R_{CC}, R_{AA}) \leq R_0 \leq R_{CC} + R_{AA}. \tag{14}$$

As R_0 is a combination of the intra-group transmission and of the coupling between these groups, two populations with a very different transmission structure can have the same R_0. For example, both the following NGM have the same R_0 (equal to 2):

$$A = \begin{pmatrix} 2 & 0 \\ 0 & 0.1 \end{pmatrix}, \quad B = \begin{pmatrix} 1 & 1 \\ 1 & 1 \end{pmatrix} \tag{15}$$

Control strategies for population A though should be target children, while control strategies for population B should involve the whole population.

2.1.5 Estimation of the number of deaths due to influenza

Tables S2 and S3 alongside Figure S2 are presented below in order to complement the text on the estimation of the number of deaths due to influenza in the main manuscript where they are referred to.

2.2 Statistical inference

2.2.1 Inference of the contacts

In the inference process, the advantages of basing the mixing structure on a resampled set of participants rather than directly on the parameters contact matrix are numerous. First, each of the resampled matrices corresponds to one mixing matrix, with the whole set of these matrices embedding the uncertainty resulting from the contact survey. Secondly, two similar datasets of participants are likely to lead to similar mixing matrices. This means that this structure defines some sort of distance in the matrix space. A 'random-walk' in this matrix space can thus be performed by resampling a certain number of participants from the current matrix to obtain a proposal mixing matrix.

[7] Equation (12) can also be derived more directly by coming back to the mathematical definition of R_0 as, when the matrix is triangular (as one of the off-diagonal value must be equal to zero if $d_R = 0$), the eigenvalues will be the diagonal values.

Table S2: Annual number of deaths attributable to influenza by age-group (mean and 95% CI) estimated from generalized linear models using the model of Hardelid et al. [1].

season	0 - 14 y	15 - 44 y	45 - 64 y	65+ y
1999/00	0 [0 - 43]	268 [11 - 525]	1175 [334 - 2016]	23567 [16187 - 30947]
2000/01	29 [0 - 120]	105 [0 - 293]	442 [21 - 864]	6523 [4661 - 8385]
2001/02	37 [0 - 143]	41 [0 - 244]	421 [0 - 844]	9434 [6079 - 12789]
2002/03	168 [54 - 283]	14 [0 - 261]	320 [0 - 799]	9908 [8043 - 11773]
2003/04	104 [0 - 218]	0 [0 - 205]	997 [504 - 1491]	10984 [8072 - 13896]
2004/05	85 [0 - 196]	112 [0 - 299]	1355 [907 - 1803]	16659 [14280 - 19037]
2005/06	66 [0 - 167]	110 [0 - 291]	912 [506 - 1318]	10185 [7414 - 12956]
2006/07	0 [0 - 0]	63 [0 - 306]	1039 [505 - 1573]	11914 [9700 - 14129]
2007/08	0 [0 - 82]	219 [11 - 428]	700 [279 - 1121]	9481 [6975 - 11988]
2008/09	0 [0 - 75]	157 [0 - 416]	1132 [588 - 1676]	15959 [13738 - 18179]

Table S3: Age-specific risk ratio of the CFRs for acute respiratory infection obtained from analysis of HES hospitalization data

age group	RR_a (sd)
0 - 14 y	20.78 (2.50)
15 - 44 y	18.45 (1.24)
45 - 64 y	6.02 (0.21)
65+ y	1.04 (0.01)

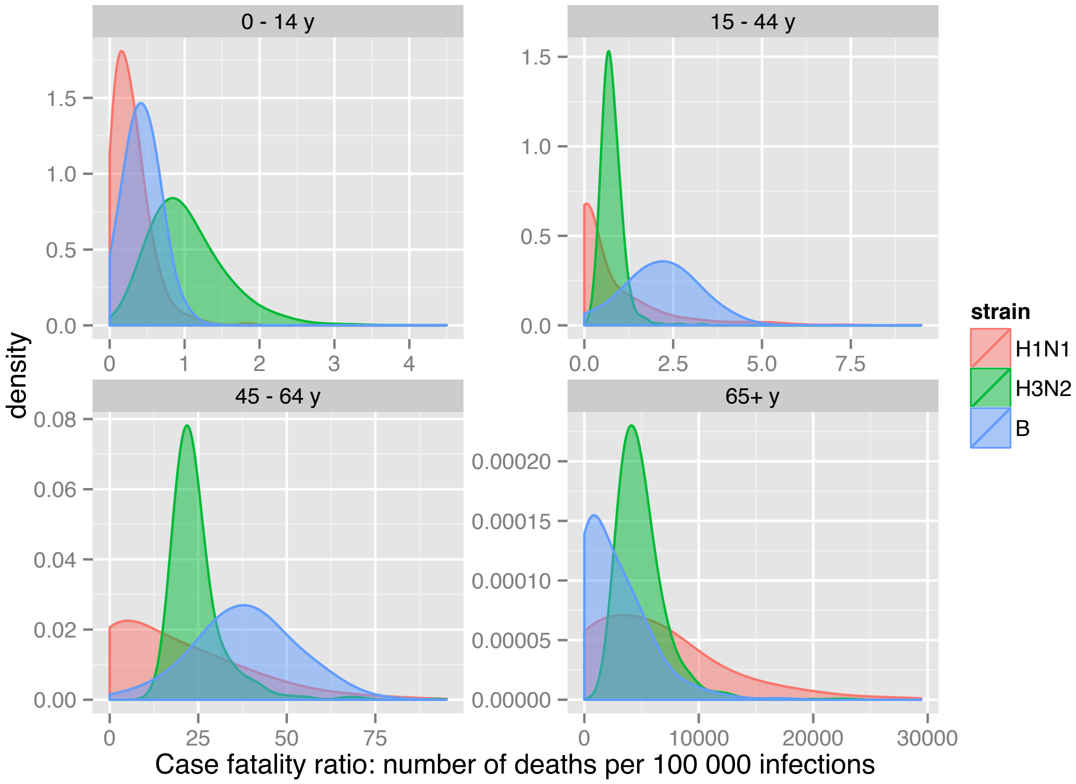

Figure S2: Distribution of the case fatality ratio (CFR) for the low risk group by age and strain estimated by generalized linear model.

2.2.2 Identifiability of the variables

Examination of Equation (2) for the force of infection λ_i among age group i shows that it is impossible to identify both the transmissibility q and the susceptibility profile $\{\sigma_i\}$ from transmission events only. Indeed, for any set $\{q, \sigma_i\}$, the family of parameters $\{q/a, a\sigma_i\}$ (for any constant a such that $a\sigma_i \leq 1$ for all i) would give the same value for the force of infection leading to the same dynamic of transmission for the disease. To avoid this identifiability problem some other sources of data are required to inform the priors of these two parameters. In the next section, we present the methods used to derived priors on susceptibility, transmissibility and the ascertainment probabilities using some serology data existing from one of the season and subtype of the study.

2.2.3 Derivation of the priors

After the season 2003/04, serology assays were performed for antibodies of the A/H3N2 subtype in sera taken before and after the influenza season [19]. This allows us to derive informative priors on susceptibility at the start of the season and the ascertainment probabilities $\{\epsilon_i\}$ for this particular year. For the other years, we use the information gained on this particular year to inform the transmissibility q and the $\{\epsilon_i\}$.

Susceptibility at the start of season 2003/04 In order to use the serology data for the derivation of susceptibility levels for the start of the 2003/04 season, we need to implement a way of deriving level of protection from infection by the influenza virus as a function of the titre of haemagglutination inhibition (HI) antibodies. The most commonly used model [40] is based on an inverse logit function of the logarithm of the titre and two parameters α and β:

$$\pi(T, \alpha, \beta) = \frac{e^{\beta(\log(T)-\alpha)}}{1 + e^{\beta(\log(T)-\alpha)}}$$

where $0 \leq \pi(T, \alpha, \beta) \leq 1$ is the protection conferred by a HI titre of T. If the baseline risk for an individual to develop influenza is λ in absence of protective antibodies, the risk to develop influenza with antibodies is $\lambda(1 - \pi(T, \alpha, \beta))$. The

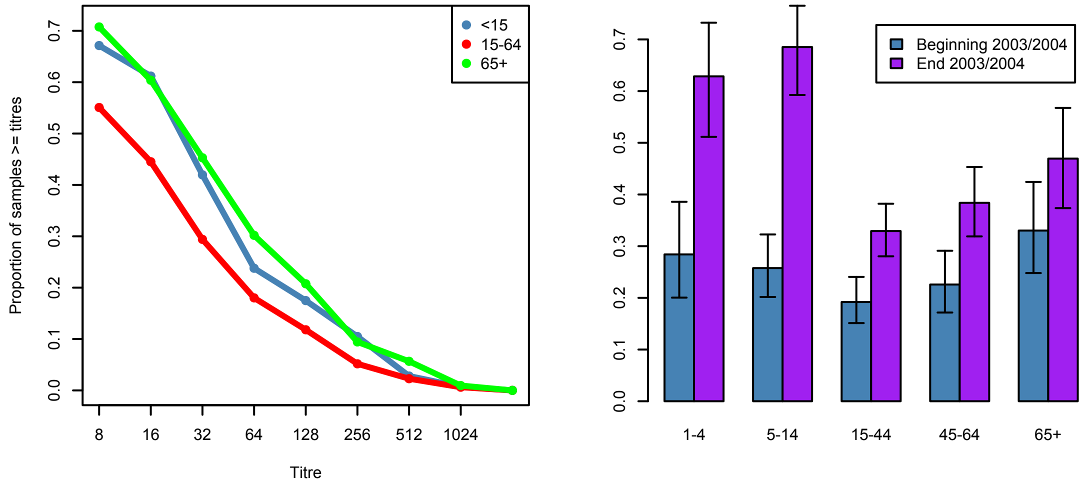

Figure S3: Serology for 2003/04 season. (left) Reverse Cumulative Distributions curves of antibody titres in the 0-14, 15-64 and 65+ year old and (right) Change in proportion of individuals with antibody ≥ 40 as presented in [19].

parameter α is the logarithm of the titre giving 50% protection and β is linked with the shape of the function. Different studies [41, 42] have shown than this relationship does not appear to depend on the nature (arising from vaccines or infection) or the subtypes of the antibodies (at least not being statistically significant). Nevertheless, Black et al. [42] have demonstrated that age was important as younger individuals need higher titres to experience the same levels of protection. Given α_i and β_i the parameters of the protection equation corresponding to the age group i, the link between the protective antibodies of the individuals belonging to the age group and the susceptibility σ_i at the population level in this age group is given by the following equation:

$$\sigma_i = 1 - \frac{1}{N_i} \sum_{j=1}^{N_i} \pi(T_j, \alpha_i, \beta_i)$$

with N_i is the number of individual in age group i and T_j the titre of the jth individual in this age group.

To establish a prior on the three inferred susceptibility levels σ_{0-14}, σ_{15-64} and σ_{65+} at the start of the season 2003/04, we use titres from a study by Johnson [19] (Figure S3). The values for the protection function are sampled using the methodology[8] and values from Coudeville et al. [41] for the age-group 1-4 year old and from Black et al. [42] for individuals older than 15 years. For the age group of the 5-14 year old for which we do not have found studies measuring the range of the α and β (the Coudeville study is restricted to the range of 6 to 72 months children), we use an intermediate value by sampling from a broad distribution encompassing the two distributions. The obtained distributions allow us to derive an estimate of the susceptibility levels of σ_{0-14}, σ_{15-64} and σ_{65+} assuming to be normally distributed (truncated to [0,1]). The values found for the susceptibility priors are summarised in Table S4.

Age group	Samples	Susceptibility (mean)	Susceptibility (s.d.)
0 to 14 y.o.	286	0.688	0.083
15 to 64 y.o.	483	0.529	0.122
65+ y.o.	106	0.523	0.175

Table S4: Derivation of the susceptibility priors

Ascertainment probability Using the set-based definition of the ascertainment probability ϵ, an estimator can be derived from the data. Indeed, as

$$\epsilon = P(B \cap E | A) = \frac{P(B \cap E)}{P(A)},$$

[8]Values of the means and standard deviations of the α and β are sampled from uniform distribution using the values given in Coudeville et al. [41] and then used to generate values for α and β from normal distributions.

ϵ is the ratio of the number of people recorded as ILI and potentially positive for the virological test ($|B \cap E|$) by the total number of people infected by influenza A/H3N2 during the season 2003/04 ($|A|$).

To calculate an estimator $\hat{m}^+_{i,tot}$ of $\sum_{j=1}^{52} m^+_{ij}$ during the year 2003/04, the number of people in the age group i who were recorded for ILI and who would have been positive to influenza A/H1N1 if tested, we summed over the season to get $\hat{m}^+_{tot} = \sum_{j=1}^{52} \frac{n^+_{ij}}{n_{ij}} m_{ij}$. To get an estimate $\hat{Z}_{i,tot}$ of the total number of infection in age group i over the season, we use the number of individual who seroconverted during this season, as defined in [19] by the difference of the proportion of people with a titre $\geq$40.

Age group	$\hat{m}^+_{tot}$	$\hat{Z}_{i,tot}$ (C.I.) in 000's	ϵ_i (mode)	Lower bound	Upper bound	Meanlog	S.D.log
0 to 14 y.o.	37174	3603 (2449,4596)	0.0103	0.00809	0.0152	-4.49	0.286
15 to 64 y.o.	64372	4952 (2303,7503)	0.0130	0.00858	0.0279	-4.12	0.475
65+ y.o.	9507	1104 (39, 2119)	0.00861	0.00449	0.243	-2.98	1.33

Table S5: Derivation of the ascertainment probability priors

As an important proportion of children seroconverted, we have a good estimate of ϵ_{0-14} while for the elderly, there is a lot of uncertainty regarding individuals who actually seroconverted.. This is reflected by the upper bound of ϵ_{65+} which is very high. To reflect the skewness of the data, we use lognormal distributions, with a mode in $\hat{m}^+_{tot}/\hat{Z}_{i,tot}$ and a mean at half of the boundaries.

Transmissibility of the virus Susceptibility levels in a population are changing due to antibody dynamics (immunity built up from past season and by decay), demographic renewal and drift of the virus. It is difficult thus to infer immunity from another season especially if distant in time or measured for a different subtype. Transmissibility, though probably not constant every year, is much more likely to remain similar from one year to another. The value of transmissibility from one year is likely to be a good prior for other year.

As susceptibility levels and transmissibility are intimately linked and cannot be measured independently, we use the value of the transmissibility inferred for the season 2003/04 in H3N2 as a prior for the other seasons and strains, while employing an uninformative prior on the susceptibility. To account for uncertainty, we have chosen as a prior for the transmissibility for the other years a normal distribution with the same mean as the posterior of season 2003/04 and 1.5 times the standard deviation.

2.2.4 Derivation of the likelihood

We start by deriving the likelihood for the full model and then derive the likelihood for the simplified model that we used for the implementation.

Likelihood of the full model with augmented data Let $\mathcal{D}$ be the set of the observable variables (i.e. our data). We are interested in inferring the parameters of the dynamical system θ, the ascertainment probabilities ϵ and the outside infection probability ψ. At the same time, we need to account of the m^+ which appear in the hierarchical component of the observation model and which can be considered as "nuisance parameters". Two options exist, the first is to treat them as normal parameters and then consider the marginal distributions from the posterior; the second is to integrate them out. These two options lead to two different possible "likelihood" functions.

In a Bayesian framework the first approach would be favoured as it allows to define for example a prior distribution on these parameters. Nevertheless, the second is equivalent, as integrating out the m^+ can be done using a prior. In term of computation, the former results in a more complex parameter space to explore while the latter leads to a more complex likelihood to compute. We present both versions of the likelihood, while we have chosen to use the second version of our likelihood which is equivalent to assuming a flat prior on the m^+ but simplify the dimension of our parameter space.

The first likelihood function $\mathcal{L}_1$ is derived from the hierarchical structure of the observation part of our model (see Figure 1 of the main paper) by breaking down the virological and GP attendance parts of the model:

$$\mathcal{L}_1(\mathcal{D}|m^+, \epsilon, \theta, \psi) = \underbrace{\mathcal{L}(\mathcal{D}_1|m^+, \theta)}_{Virology} \underbrace{\mathcal{L}(\mathcal{D}_2|\epsilon, \psi, m^+, \theta)}_{GP}, \quad (16)$$

with $\mathcal{D} = \{\mathcal{D}_1, \mathcal{D}_2\}$.

The virological testing is independent of θ so the first part of Equation (16) is given by the hypergeometric distribution of n_{ij}^{+}, the probability of getting exactly n_{ij}^{+} positive among the n_{ij} samples drawn at week j among age group i is:

$$\begin{aligned}\mathcal{L}(\mathcal{D}_1|m^{+},\theta) &= P(\mathcal{D}_1 = \{m_{ij}, n_{ij}^{+}, n_{ij}\}|m^{+}) \\ &= \prod_{i=1}^{5}\prod_{j=1}^{52} \frac{\binom{m_{ij}^{+}}{n_{ij}^{+}}\binom{m_{ij}-m_{ij}^{+}}{n_{ij}-n_{ij}^{+}}}{\binom{m_{ij}}{n_{ij}}} \qquad (17)\end{aligned}$$

The second part of the likelihood, describing the model of attendance to the GP and test sensitivity is a sum of two random variables and thus its distribution is the discrete convolution of the two distributions $\mathcal{B}(z_{ij}^{\theta}, \epsilon_i)$ and $\mathcal{P}(\psi\epsilon_i N_{ij}^{mon})$:

$$\begin{aligned}P(\mathcal{D}_2|\epsilon, m^{+},\theta,\psi) &= \prod_{i=1}^{5}\prod_{j=1}^{52}\sum_{h=h_{ij}^{min}}^{m_{ij}^{+}} \left[\binom{z_{ij}^{\theta}}{m_{ij}^{+}-h}\epsilon_i^{m_{ij}^{+}-h}(1-\epsilon_i)^{z_{ij}^{\theta}-m_{ij}^{+}+h}\frac{(\psi N_{ij}^{mon}\epsilon_i)^h}{h!}e^{-\psi N_{ij}^{mon}\epsilon_i}\right] \\ &= \prod_{i=1}^{5}\prod_{j=1}^{52}\sum_{h=h_{ij}^{min}}^{m_{ij}^{+}} \left[\binom{z_{ij}^{\theta}}{m_{ij}^{+}-h}\epsilon_i^{m_{ij}^{+}}(1-\epsilon_i)^{z_{ij}^{\theta}-m_{ij}^{+}+h}\frac{(\psi N_{ij}^{mon})^h}{h!}e^{-\psi N_{ij}^{mon}\epsilon_i}\right], \qquad (18)\end{aligned}$$

with $h_{ij}^{min} = \max(m_{ij}^{+} - z_{ij}^{\theta}, 0)$ and z_{ij}^{θ} the number of cases in age group i predicted in week j by the model given the parameters θ. Hence, by combining (17) and (18) in (16), we obtain the complete likelihood for one season:

$$\mathcal{L}_1(\mathcal{D}|m^{+},\epsilon,\theta,\psi) = \prod_{i=1}^{5}\prod_{j=1}^{52} \frac{\binom{m_{ij}^{+}}{n_{ij}^{+}}\binom{m_{ij}-m_{ij}^{+}}{n_{ij}-n_{ij}^{+}}}{\binom{m_{ij}}{n_{ij}}} \sum_{h=h_{ij}^{min}}^{m_{ij}^{+}} \left[\binom{z_{ij}^{\theta}}{m_{ij}^{+}-h}\epsilon_i^{m_{ij}^{+}}(1-\epsilon_i)^{z_{ij}^{\theta}-m_{ij}^{+}+h}\frac{(\psi N_{ij}^{mon})^h}{h!}e^{-\psi N_{ij}^{mon}\epsilon_i}\right]. \qquad (19)$$

Likelihood of the full model with summation over $\mathbf{m_{ij}^{+}}$ It is possible to derive a likelihood function free from the m_{ij}^{+} by summing them over all the values they can take, for this we need to find first the lower and upper boundaries of all possible m_{ij}^{+}.

Using the set notation with the indices i and j denoting respectively the age-group and the week, we have:

$$E_{ij} = (E_{ij} \cap B_{ij}) \cup (E_{ij} \cap \bar{B}_{ij}) \qquad (20)$$

As $B_{ij} \cap \bar{B}_{ij} = \emptyset$, we can combine the values from (3) with Equation (20) to get:

$$m_{ij} = m_{ij}^{+} + |E_{ij} \cap \bar{B}_{ij}| \qquad (21)$$

and, from $(F_{ij} \cap \bar{B}_{ij}) \subseteq (E_{ij} \cap \bar{B}_{ij})$ we have the inequality:

$$m_{ij} \geq m_{ij}^{+} + |F_{ij} \cap \bar{B}_{ij}|. \qquad (22)$$

Using the same decomposition for F_{ij} than (20) for E_{ij}

$$F_{ij} = (F_{ij} \cap B_{ij}) \cup (F_{ij} \cap \bar{B}_{ij}), \qquad (23)$$

we obtain, plugin in the values from (3) and $B_{ij} \cap \bar{B}_{ij} = \emptyset$:

$$|F_{ij} \cap \bar{B}_{ij}| = n_{ij} - n_{ij}^{+}. \qquad (24)$$

Then, by combining (22) with (24) we obtain the first upper boundary for m_{ij}^{+}:

$$m_{ij}^{+} \leq m_{ij} - n_{ij} + n_{ij}^{+}. \qquad (25)$$

Finally using $(B_{ij} \cap F_{ij}) \subseteq (B_{ij} \cap E_{ij})$, and thus $n_{ij}^{+} \leq m_{ij}^{+}$, we get the final boundaries for m_{ij}^{+}:

$$n_{ij}^{+} \leq m_{ij}^{+} \leq \min(z_{ij}^{\theta}, m_{ij} - n_{ij} + n_{ij}^{+}) \qquad (26)$$

Hence, if we define $k_{ij}^{max} = \min(z_{ij}^{\theta}, m_{ij} - n_{ij} + n_{ij}^{+})$, the complete likelihood for one season is equal to:

$$\mathcal{L}_2(\mathcal{D}|\epsilon,\theta,\psi) = \prod_{i=1}^{5}\prod_{j=1}^{52}\sum_{k=n_{ij}^{+}}^{k_{ij}^{max}} \left[\frac{\binom{k}{n_{ij}^{+}}\binom{m_{ij}-k}{n_{ij}-n_{ij}^{+}}}{\binom{m_{ij}}{n_{ij}}} \sum_{h=h_{ij}^{min}}^{k}\left[\binom{z_{ij}^{\theta}}{k-h}\epsilon_i^{k}(1-\epsilon_i)^{z_{ij}^{\theta}-k+h}\frac{(\psi N_{ij}^{mon})^h}{h!}e^{-\psi N_{ij}^{mon}\epsilon_i}\right]\right] \qquad (27)$$

with z_{ij}^{θ} the number of cases in the monitored population in age group i predicted in week j by the model given the parameters θ.

2.3 Implementation

We present here some details about the implementation of our study. Though the work has been coded in C for speed and flexibility, we describe the used algorithm using pseudo-code. Transcription in other programming languages should be easy.

2.3.1 Implementation of the epidemic model

We implement the epidemic model using a simple Euler integration with a time step of 0.25 days.

2.3.2 Proposal for the contact matrix

The Metropolis-Hastings algorithms works efficiently when the proposed set of parameters at each step leads to a reasonable acceptance rate in order to provide good mixing. A trade off needs to be found between high acceptance of proposed parameters close to the current ones, leading to slow mixing, and low acceptance of more distant values leading to poor efficiency of the algorithms and high correlation of the sampled values. For this, one needs to be able to define a metric on the parameter sets to determine if the proposed values are close or far from the current ones and adapt them (whether manually or using more sophisticated approaches). It is relatively easy to define such a distance for real valued parameters (though tuning the actual proposal for high dimensional parameters remains a challenging problem). Here, we propose a way of efficiently producing proposals for contact matrices "near" the current matrix in a reversible way (condition necessary for the validity of the resulting Markov chain). The aim of this section is not to define formally the proposed solution in terms of distance[9] in a discrete matrix spaces but to explain our approach and allow reproducibility of our results.

Instead of tracking the transmission matrix itself (or a contact matrix of some sort), we track a representative set of contacts. The update is done on the set of contacts rather than the transmission (or contact) matrix coefficients by resampling the original contact survey (POLYMOD) with replacement. The more similar the set of contacts, the more similar the transmission will be, and one can tune the extent of possible change at each step by allowing j substitution with a probability p_j. When this selection is done with a flat likelihood, the random walk on the matrices resulting from progressively updating the set of contacts produces a sampled of transmission matrix with the expected matrix being the POLYMOD matrix and a variance being the one of the original survey. This method allows for the inclusion of the transmission matrix into the inference process, with the transmission matrix effectively representing an additional parameter of the model.

2.3.3 Implementation of the likelihood

To shorten the computation time, we need to optimize the calculation of the likelihood function. We start by reordering the terms in Equation (27):

$$\mathcal{L}_2(\mathcal{D}|\epsilon,\theta,\psi) = \prod_{i=1}^{5}\prod_{j=1}^{52}\sum_{k=n_{ij}^{+}}^{k_{ij}^{max}}\sum_{h=h_{ij}^{min}}^{k}\left[\frac{\binom{k}{n_{ij}^{+}}\binom{m_{ij}-k}{n_{ij}-n_{ij}^{+}}}{\binom{m_{ij}}{n_{ij}}}\binom{z_{ij}^{\theta}}{k-h}\epsilon_i^k(1-\epsilon_i)^{z_{ij}^{\theta}-k+h}\frac{(\psi N_{ij}^{mon})^h}{h!}e^{-\psi N_{ij}^{mon}\epsilon_i}\right].$$

In order to be used by the Metropolis-Hasting algorithm, the likelihood function needs to be defined up to a multiplicative constant. This means that the computation of any multiplicative factors not depending on ϵ, θ or ψ can be omitted in order to speed up the calculation. As $\binom{m_{ij}}{n_{ij}}$ is only dependent on the data, we have:

$$\mathcal{L}_2(\mathcal{D}|\epsilon,\theta,\psi) \propto \prod_{i=1}^{5}\prod_{j=1}^{52}\sum_{k=n_{ij}^{+}}^{k_{ij}^{max}}\sum_{h=h_{ij}^{min}}^{k}\left[\binom{k}{n_{ij}^{+}}\binom{m_{ij}-k}{n_{ij}-n_{ij}^{+}}\binom{z_{ij}^{\theta}}{k-h}\epsilon_i^k(1-\epsilon_i)^{z_{ij}^{\theta}-k+h}\frac{(\psi N_{ij}^{mon})^h}{h!}e^{-\psi N_{ij}^{mon}\epsilon_i}\right]$$

which can be expanded using factorials:

$$\begin{aligned}\mathcal{L}_2(\mathcal{D}|\epsilon,\theta,\psi) \quad \propto \quad & \prod_{i=1}^{5}\prod_{j=1}^{52}\sum_{k=n_{ij}^{+}}^{k_{ij}^{max}}\sum_{h=h_{ij}^{min}}^{k}\left[\frac{k!(m_{ij}-k)!z_{ij}^{\theta}!}{n_{ij}^{+}!(k-n_{ij}^{+})!(n_{ij}-n_{ij}^{+})!(m_{ij}+n_{ij}^{+}-k-n_{ij})!(k-h)!(z_{ij}^{\theta}-k+h)!}\right.\\ & \left.\epsilon_i^k(1-\epsilon_i)^{z_{ij}^{\theta}-k+h}\frac{(\psi N_{ij}^{mon})^h}{h!}e^{-\psi N_{ij}^{mon}\epsilon_i}\right] \qquad (28)\end{aligned}$$

[9] We refer here only loosely to the notion of distance, in an intuitive way and for explanatory purposes, without trying to defining any rigorous metrics.

and can be further simplified noticing that n_{ij}^+ and n_{ij} are observable (and thus constant):

$$\mathcal{L}_2(\mathcal{D}|\epsilon,\theta,\psi) \propto \prod_{i=1}^{5}\prod_{j=1}^{52}\sum_{k=n_{ij}^+}^{k_{ij}^{max}}\sum_{h=h_{ij}^{min}}^{k}\left[\frac{k!(m_{ij}-k)!z_{ij}^{\theta}!}{(k-n_{ij}^+)!(m_{ij}+n_{ij}^+-k-n_{ij})!(k-h)!(z_{ij}^{\theta}-k+h)!}\right.$$
$$\left.\epsilon_i^k(1-\epsilon_i)^{z_{ij}^{\theta}-k+h}\frac{(\psi N_{ij}^{mon})^h}{h!}e^{-\psi N_{ij}^{mon}\epsilon_i}\right]. \quad (29)$$

The computation of the likelihood function involves the summation of the "elementary" terms inside the square brackets in two directions to get a factor which is part of a bigger product involving each week and age group. Figure S4 depicts the surface of summation for one week and age group.

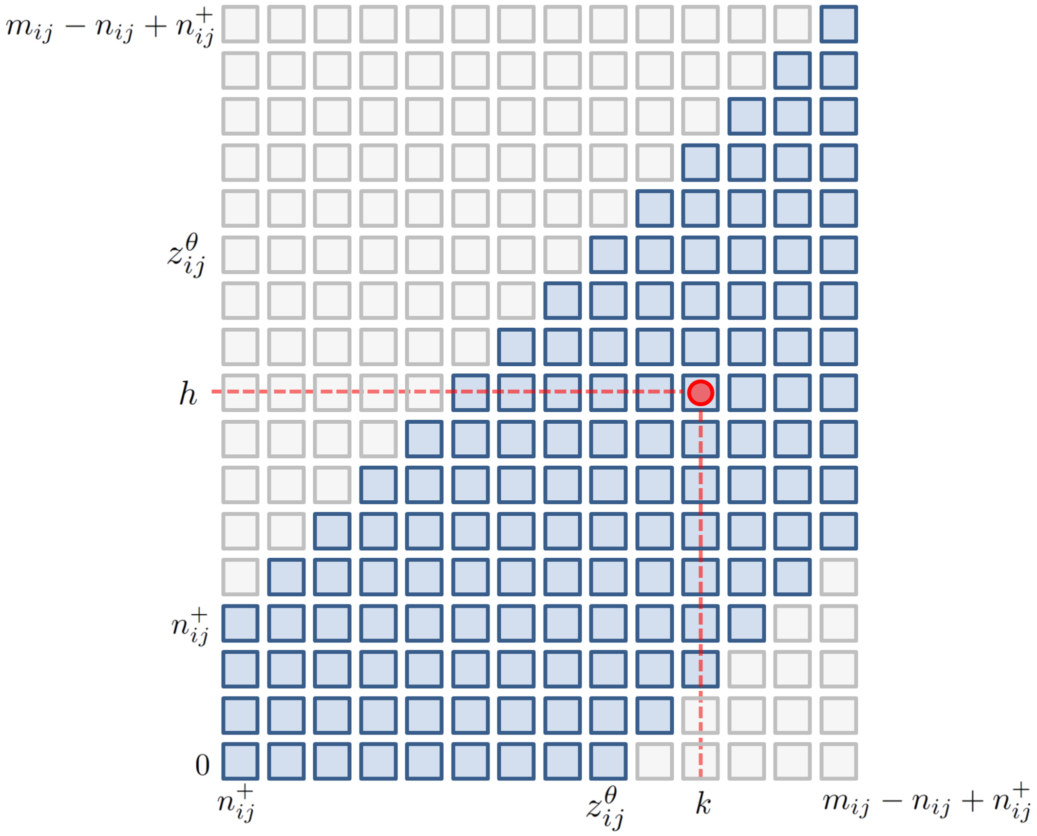

Figure S4: Surface of summation of the likelihood function for a given week i and age group j. The red dot is giving the position of a_{kh}^{ij}. The calculation of the likelihood involves the summation of all the blue squares of the corresponding a_{kh}^{ij} for each week and age group.

Each of the blue squares represents one of the term of the double sum in Equation (29) for a given i and j. If we call a_{kh}^{ij} the term at position (k, h), we have:

$$a_{kh}^{ij} = \frac{k!(m_{ij}-k)!z_{ij}^{\theta}!}{(k-n_{ij}^+)!(m_{ij}+n_{ij}^+-k-n_{ij})!(k-h)!(z_{ij}^{\theta}-k+h)!}\epsilon_i^k(1-\epsilon_i)^{z_{ij}^{\theta}-k+h}\frac{(\psi N_{ij}^{mon})^h}{h!}e^{-\psi N_{ij}^{mon}\epsilon_i} \quad (30)$$

From a practical point of view, the summation can be made in any order as soon as all the "blue squares" are integrated. Equation (27) suggests a summation by columns (by varying first the h) but any other scheme is valid. For example, the likelihood can be computed by summing over the lines (h constant, k varying) or the diagonals ($h - k$ constant, h and k varying). The summation scheme will exploit that neighbouring a_{kh}^{ij} will share an important number of factors in common and thus passing from one to another will only involves relatively simple calculations based on a recursive scheme.

For example, the following system, presenting respectively vertical, horizontal and diagonal increments allows to compute all the a^{ij}_{kh}:

$$\begin{cases} a^{ij}_{k,h} &= \frac{k-h+1}{z^{\theta}_{ij}-k+h}\frac{\psi N^{mon}_{ij}}{h}(1-\epsilon_i)a^{ij}_{k,h-1} \\ a^{ij}_{k,h} &= \frac{k(m_{ij}+n^+_{ij}-k-n_{ij}+1)(z^{\theta}_{ij}-k+h+1)}{(m_{ij}-k+1)(k-n^+_{ij})(k-h)}\frac{\epsilon_i}{1-\epsilon_i}a^{ij}_{k-1,h} \\ a^{ij}_{k,h} &= \frac{k(m_{ij}+n^+_{ij}-k-n_{ij}+1)}{(m_{ij}-k+1)(k-n^+_{ij})}\frac{\psi N^{mon}_{ij}\epsilon_i}{h}a^{ij}_{k-1,h-1} \end{cases} \tag{31}$$

starting from any value of a^{ij}_{kh} for example $a^{ij}_{n^+_{ij},n^+_{ij}}$:

$$a^{ij}_{n^+_{ij},n^+_{ij}} = \frac{(m_{ij}-n^+_{ij})!}{(m_{ij}-n_{ij})!}\epsilon_i^{n^+_{ij}}(1-\epsilon_i)^{z^{\theta}_{ij}}(\psi N^{mon}_{ij})^{n^+_{ij}}e^{-\psi N^{mon}_{ij}\epsilon_i}. \tag{32}$$

The sum a^{ij}_{sum} of the $a^{ij}_{k,h}$

$$a^{ij}_{sum} = \sum_{k=n^+_{ij}}^{k^{max}_{ij}}\sum_{h=h^{min}_{ij}}^{k} a^{ij}_{k,h} \tag{33}$$

allows us to derive the likelihood

$$\mathcal{L}_2(\mathcal{D}|\epsilon,\psi,\theta) \propto \prod_{i=1}^{5}\prod_{j=1}^{52} a^{ij}_{sum}$$

which is equivalent to the more computing-friendly form:

$$\log(\mathcal{L}_2(\mathcal{D}|\epsilon,\psi,\theta)) = \sum_{i=1}^{5}\sum_{j=1}^{52}\log a^{ij}_{sum} + constant. \tag{34}$$

The value of the constant is equal to $\sum_{i=1}^{5}\sum_{j=1}^{52}\log\left(\frac{m_{ij}!}{n_{ij}!(m_{ij}-n_{ij})!nijp!(n_{ij}-n^+_{ij})!}\right)$ which is typically extremely big. The use of the log-likelihood rather the likelihood allows to avoid numerical problems.

Approximation of the likelihood In order to speed up the computation of Equation (34), we use an approximation of the a^{ij}_{sum}. For this, we start by reordering the terms in Equation (33):

$$a^{ij}_{sum} = \sum_{h=0}^{m_{ij}-n_{ij}+n^+_{ij}}\sum_{k=\max(n^+_{ij},h)}^{\min(z^{\theta}_{ij}+h,m_{ij}-n_{ij}+n^+_{ij})} a^{ij}_{k,h}, \tag{35}$$

which, given a constant d, the RHS of Equation (35) can be broken into two parts:

$$a^{ij}_{sum} = \left(\sum_{h=0}^{h^{trunc}_{ij}}\sum_{k=\max(n^+_{ij},h)}^{\min(z^{\theta}_{ij}+h,m_{ij}-n_{ij}+n^+_{ij})} a^{ij}_{k,h}\right) + b_{ij} \tag{36}$$

with $h^{trunc}_{ij} = \min(d, m_{ij}-n_{ij}+n^+_{ij})$ and

$$\begin{cases} b_{ij} &= \sum_{h=d+1}^{m_{ij}-n_{ij}+n^+_{ij}}\sum_{k=\max(n^+_{ij},h)}^{\min(z^{\theta}_{ij}+h,m_{ij}-n_{ij}+n^+_{ij})} a^{ij}_{k,h} \quad \text{if } d < m_{ij}-n_{ij}+n^+_{ij}, \\ b_{ij} &= 0 \quad \text{if } d \geq m_{ij}-n_{ij}+n^+_{ij}. \end{cases}$$

Equation (36) splits the summation surface (Figure S4) into an upper ($h > d$) and a lower ($h \leq d$) part. By definition of the observation process, the height (indexed by h) of a cell represents the outcome of the random variable $m^{outside}_{ij}$ (see description of the observation model in the main paper) i.e. the number of people recorded as ILI who have been infected outside of the main modelled epidemic. The upper part of the summation surface corresponds thus to the outcomes where $m^{outside}_{ij} > d$ and, as the sum over the entire surface is defined in Equation (33) as being equal to a^{ij}_{sum}, b_{ij} can be expressed as:

$$b_{ij} = a^{ij}_{sum}P(m^{outside}_{ij} > d) \tag{37}$$

By combining Equations (36) and (37), we thus get:

$$a_{sum}^{ij} = \frac{\sum_{h=0}^{h_{ij}^{trunc}} \sum_{k=\max(n_{ij}^{+},h)}^{\min(z_{ij}^{\theta}+h, m_{ij}-n_{ij}+n_{ij}^{+})} a_{k,h}^{ij}}{1 - P(m_{ij}^{outside} > d)}. \tag{38}$$

As $m_{ij}^{outside}$ is Poisson distributed with coefficient $\psi N_{ij}^{mon}\epsilon_i \ll 1$, we have $P(m_{ij}^{outside} > d) \approx 0$ and:

$$a_{sum}^{ij} \approx \sum_{h=0}^{h_{ij}^{trunc}} \sum_{k=\max(n_{ij}^{+},h)}^{\min(z_{ij}^{\theta}+h, m_{ij}-n_{ij}+n_{ij}^{+})} a_{k,h}^{ij}. \tag{39}$$

We thus can define the truncated likelihood $\mathcal{L}_3^d$ by:

$$\mathcal{L}_3^d(\mathcal{D}|\epsilon, \psi, \theta) = \prod_{i=1}^{5}\prod_{j=1}^{52}\left[\sum_{h=0}^{h_{ij}^{trunc}} \sum_{k=\max(n_{ij}^{+},h)}^{\min(z_{ij}^{\theta}+h, m_{ij}-n_{ij}+n_{ij}^{+})} a_{k,h}^{ij}\right] \tag{40}$$

with $h_{ij}^{trunc} = \min(d, m_{ij} - n_{ij} + n_{ij}^{+})$.

Relationship between $\mathcal{L}_2(\mathcal{D}|\epsilon,\psi,\theta)$ and $\mathcal{L}_3^d(\mathcal{D}|\epsilon,\psi,\theta)$ Given the range of values taken by ψ and ϵ_i during the inference, computing $\log(\mathcal{L}_3^d(\mathcal{D}|\epsilon,\psi,\theta))$ is equivalent in practice (and much quicker) than computing $\log(\mathcal{L}_2(\mathcal{D}|\epsilon,\psi,\theta))$. More precisely, by plugging in (38) into (34) we have:

$$\begin{aligned}
\log(\mathcal{L}_2(\mathcal{D}|\epsilon,\psi,\theta)) &= \sum_{i=1}^{5}\sum_{j=1}^{52}\left[\log\left(\sum_{h=0}^{h_{ij}^{trunc}} \sum_{k=\max(n_{ij}^{+},h)}^{\min(z_{ij}^{\theta}+h, m_{ij}-n_{ij}+n_{ij}^{+})} a_{k,h}^{ij}\right) - \log\left(1 - P(m_{ij}^{outside} > d)\right)\right] \\
&\quad + \sum_{i=1}^{5}\sum_{j=1}^{52}\log\left(\frac{m_{ij}!}{n_{ij}!(m_{ij}-n_{ij})!nijp!(n_{ij}-n_{ij}^{+})!}\right) \\
&= \log(\mathcal{L}_3^d(\mathcal{D}|\epsilon,\psi,\theta)) - \sum_{i=1}^{5}\sum_{j=1}^{52}\log\left(1 - P(m_{ij}^{outside} > d)\right) \\
&\quad + \sum_{i=1}^{5}\sum_{j=1}^{52}\log\left(\frac{m_{ij}!}{n_{ij}!(m_{ij}-n_{ij})!nijp!(n_{ij}-n_{ij}^{+})!}\right)
\end{aligned}$$

As $\sum_{i=1}^{5}\sum_{j=1}^{52}\log\left(\frac{m_{ij}!}{n_{ij}!(m_{ij}-n_{ij})!nijp!(n_{ij}-n_{ij}^{+})!}\right)$ is constant and

$$\sum_{i=1}^{5}\sum_{j=1}^{52}\log\left(1 - P(m_{ij}^{outside} > d)\right) \approx 0,$$

in the range of values considered, we get:

$$\log\left(\mathcal{L}_2(\mathcal{D}|\epsilon,\psi,\theta)\right) \approx \log(\mathcal{L}_3^d(\mathcal{D}|\epsilon,\psi,\theta)) + constant. \tag{41}$$

Algorithm to compute the approximate likelihood To compute the approximate likelihood function $\mathcal{L}_3^d(\mathcal{D}|\epsilon,\theta,\psi)$, we need to define an initial value and a summation path over the reduced surface of summation. For computational reasons, as the values of the terms in the sum decrease quickly as h increases, it is best to start with the lowest line, and then sum line by line until reaching the line at height d. The height of the lowest line is given by:

$$h_{init} = \max(n_{ij}^{+} - z_{ij}^{\theta}, 0). \tag{42}$$

If $h_{init} > d$ then the surface of summation is empty and $\mathcal{L}_3^d(\mathcal{D}|\epsilon, \theta, \psi) = 0$. Otherwise, the first value ($k = n_{ij}^+$) on the line can be computed using the following set of equations:

$$\begin{cases} a_{init}^{ij} = a_{n_{ij}^+,0}^{ij} = \frac{(m_{ij}-n_{ij}^+)!}{(m_{ij}-n_{ij})!} \frac{z_{ij}^\theta !}{(z_{ij}^\theta - n_{ij}^+)!} \epsilon_i^{n_{ij}^+} (1-\epsilon_i)^{z_{ij}^\theta - n_{ij}^+} e^{-\psi \epsilon_i N_{ij}^{mon}}, & \text{if } h_{init} = 0, \\ a_{init}^{ij} = a_{n_{ij}^+, n_{ij}^+ - z_{ij}^\theta}^{ij} = \frac{(m_{ij}-n_{ij}^+)!}{(m_{ij}-n_{ij})!} \frac{n_{ij}^+ !}{(n_{ij}^+ - z_{ij}^\theta)!} \epsilon_i^{n_{ij}^+} (\psi N_{ij}^{mon})^{n_{ij}^+ - z_{ij}^\theta} e^{-\psi \epsilon_i N_{ij}^{mon}}, & \text{if } h_{init} = n_{ij}^+ - z_{ij}^\theta. \end{cases} \tag{43}$$

The calculation of the initial seeding value from System (43) not explicitly calculating the factorial function will depend on the different possible values of n_{ij}, n_{ij}^+ and z_{ij}^θ. We can break down the different element of a_{init}^{ij} and evaluate them for each of the different possibilities:

$$a_{init}^{ij} = B_{ij} C_{ij} D_{ij} \epsilon_i^{n_{ij}^+} e^{-\psi \epsilon_i N_{ij}^{mon}} \tag{44}$$

with the values of B_{ij}, C_{ij} and D_{ij} as follows:

<table>
<tr><td></td><td colspan="2">B_{ij}</td><td colspan="3">C_{ij}</td><td rowspan="2">D_{ij}</td></tr>
<tr><td></td><td>$n_{ij}^+ = n_{ij}$</td><td>$n_{ij}^+ \neq n_{ij}$</td><td>$z_{ij}^\theta = n_{ij}^+$</td><td colspan="2">$z_{ij}^\theta \neq n_{ij}^+$</td></tr>
<tr><td rowspan="2">$n_{ij}^+ \leq z_{ij}^\theta$
$(h_{init} = 0)$</td><td rowspan="4">1</td><td rowspan="4">$\prod_{g=n_{ij}^+}^{n_{ij}-1} (m_{ij} - g)$</td><td rowspan="4">$n_{ij}^+!$</td><td>$n_{ij}^+ = 0$</td><td>$n_{ij}^+ > 0$</td><td rowspan="2">$(1-\epsilon_i)^{z_{ij}^\theta - n_{ij}^+}$</td></tr>
<tr><td>1</td><td>$\prod_{g=0}^{n_{ij}^+ - 1} (z_{ij}^\theta - g)$</td></tr>
<tr><td rowspan="2">$n_{ij}^+ \geq z_{ij}^\theta$
$(h_{init} = n_{ij}^+ - z_{ij}^\theta)$</td><td>$z_{ij}^\theta = 0$</td><td>$z_{ij}^\theta > 0$</td><td rowspan="2">$(\psi N_{ij}^{mon})^{n_{ij}^+ - z_{ij}^\theta}$</td></tr>
<tr><td>1</td><td>$\prod_{g=0}^{z_{ij}^\theta - 1} (n_{ij}^+ - g)$</td></tr>
</table>

Algorithm 1 Calculation of INIT_SUM(i,j), the first value of the sum for age group i, week j

$a \leftarrow \epsilon_i^{n_{ij}^+} e^{-\psi \epsilon_i N_{ij}^{mon}}$
if $n_{ij}^+ < n_{ij}$ **then**
  for $g = n_{ij}^+$ to $n_{ij} - 1$ **do**
    $a \leftarrow a * (m - g)$
  end for
end if
if $(z_{ij}^\theta = n_{ij}^+)$ and $(z_{ij}^\theta > 0)$ **then**
  for $g = 1$ to n_{ij}^+ **do**
    $a \leftarrow a * g$
  end for
end if
if $(n_{ij}^+ > 0)$ and $(n_{ij}^+ < z_{ij}^\theta)$ **then**
  for $g = 0$ to $n_{ij}^+ - 1$ **do**
    $a \leftarrow a * (z_{ij}^\theta - g)$
  end for
end if
if $(z_{ij}^\theta > 0)$ and $(n_{ij}^+ > z_{ij}^\theta)$ **then**
  for $g = 0$ to $z_{ij}^\theta - 1$ **do**
    $a \leftarrow a * (n_{ij}^+ - g)$
  end for
end if
if $n_{ij}^+ > z_{ij}^\theta$ **then**
  $a \leftarrow a * (\psi N_{ij}^{mon})^{n_{ij}^+ - z_{ij}^\theta}$
end if
if $n_{ij}^+ < z_{ij}^\theta$ **then**
  $a \leftarrow a * (1 - \epsilon_i)^{z_{ij}^\theta - n_{ij}^+}$
end if
return a

When the seed value for the first line is calculated (see Algorithm 1), it is possible to start calculating and summing over the values of the first line by iteration of the k's between n^+_{ij} and $\min(z^\theta_{ij} + h_{init}, m_{ij} - n_{ij} + n^+_{ij})$ using the relationship given in the System (31) (horizontal increment). Once all the values of the first line are summed up, the initial seed is used to calculate the value of the seed for the upper line. The vertical increment of System (31) is used if $h \leq n^+_{ij}$, else the diagonal increment is used. Then the calculation and summing of the values of the second line are performed. The same process is repeated to calculate iteratively the values on the lines above until reaching the height d. Figure S5, depicts the summation surface and paths for the truncated likelihood calculation and in Algorithm 2, some code is provided.

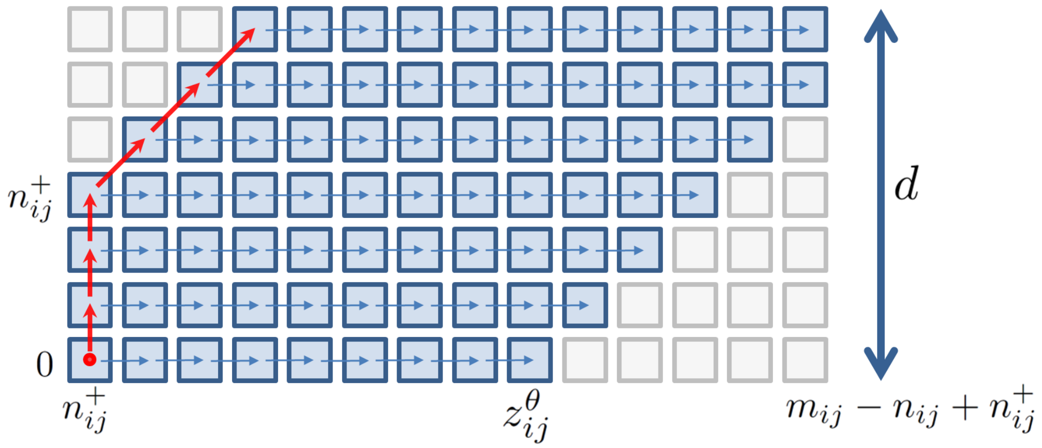

Figure S5: Surface of summation of the truncated likelihood function for a given week i and age group j. The red dot is giving the starting position of recursive calculation.

In practice Even extremely small values of d's give good results for the inference. For example, for our dataset, even a value of $d = 0$ works for most seasons. Only for the season 2008/09, with a huge increase in sampling due to the testing for the new pandemic strain, a value of $d = 2$ was chosen.

2.3.4 Implementation of the Markov chain Monte Carlo

Reduction of the dimension of the parameter space The use of the likelihood with summation over the m^+_{ij} (Equation (27) simplified in (40)) rather than the original likelihood (19) allowed us to reduce the dimension of the parameter space to explore by 260 for each of the years considered (52 weeks in 5 age groups). Furthermore, to avoid overfitting and stabilise estimates, susceptibility was grouped into three age bands, children (<15), adults (15-64), and the elderly (65+), as was the ascertainment probability, ϵ_i.

The dimension of the final model used for the inference is thus considerably lower than the original model (Table S6). While we would have need to estimate 11,466 parameters in the original model (273 * 3 subtypes * 14 seasons) and 42 sets of contacts, this has dropped to 378 parameters with 42 sets of contacts in the simplified model. This considerably simplifies the parameter space to explore even if its geometry remains complex with highly correlated variable and banana shaped[10] connected parameters.

In the rest of the section, we use the notation $\Theta = \{\epsilon_{0-14}, \epsilon_{15-64}, \epsilon_{65+}, \psi, q, \sigma_{0-14}, \sigma_{15-64}, \sigma_{65+}, l\}$ to describe the set of inferred parameters.

Proposal mechanism and adaption We are interesting in drawing from the posterior of Θ and the set of resampled contacts. For this, we used a proposal mechanism combining an Adaptive Metropolis type proposal [43] for Θ with the replacement of 1 contact in the current set $\mathcal{A}$ at probability 0.1.

The proposal function $Q(x, .)$ for the Adaptive Metropolis is defined as follow:

$$Q_n(x, .) = (1 - \zeta)\mathcal{N}\left(x, (2.38)^2 \frac{\Sigma_n}{d}\right) + \zeta\mathcal{N}\left(x, (0.1)^2 \frac{I_d}{d}\right) \quad (45)$$

[10]By this we mean that some pairs of parameters presents banana-shaped likelihood function if projected in the plane of these parameters (this can be visualised by making a scatterplot of the concerned sampled parameters), making the exploration of this region of the parameter space difficult.

Algorithm 2 Calculation of the truncated log-likelihood $\log(\mathcal{L}_3^d)$

```
LL ← 0
for i = 1 to 5 do
  for j = 1 to 52 do
    h_init ← max(n+_ij − z^θ_ij, 0)
    if h_init > d then
      return -10,000
    end if
    h_max ← min(d, m_ij − n_ij + n+_ij)
    for h = h_init to h_max do
      if h = h_init then
        k_seed ← n+_ij
        a_seed ← INIT_SUM(i,j) {Calculation of the first value of the sum}
      end if
      if h > n+_ij then
        k_seed ← k_seed + 1
        a_seed ← a_seed * [k(m_ij+n+_ij−k−n_ij+1) / ((m_ij−k+1)(k−n+_ij))] [ψ N^mon_ij ε_i / h] {Diagonal increment}
      end if
      if 0 < h ≤ n+_ij then
        a_seed ← a_seed * [(k−h+1) / (z^θ_ij−k+h)] [ψ N^mon_ij / h] (1 − ε_i) {Vertical increment}
      end if
      a ← a_seed
      a_sum ← a_sum + a
      k_max ← min(z^θ_ij + h, m_ij − n_ij + n+_ij)
      if k_max > k_seed then
        for k = k_seed + 1 to k_max do
          a ← a * [k(m_ij+n+_ij−k−n_ij+1)(z^θ_ij−k+h+1) / ((m_ij−k+1)(k−n+_ij)(k−h))] [ε_i / (1−ε_i)] {Iterative calculation of the elements of the h^th line}
          a_sum ← a_sum + a
        end for
      end if
    end for
    LL ← LL + log(a_sum)
  end for
end for
return LL
```

where Σ_n is the empirical variance-covariance matrix defined as the unbiased estimator of the variance-covariance matrix:

$$\Sigma_n = \frac{1}{n-1}\sum_{k=1}^{n}\left(\Theta^k - \bar{\Theta}\right)\left(\Theta^k - \bar{\Theta}\right)^T \tag{46}$$

with $\bar{\Theta} = \frac{1}{n}\sum_{k=1}^{n}\Theta^k$ the empirical mean of the samples Θ^k. The chain $\{\Theta^k\}_{1\leq k\leq n}$ loses the Markov property it has with other proposal functions (e.g. for symmetric random walks), but fulfill the Diminishing Adaptation and Bounded Convergence conditions [43] which guarantees the ergodicity and asymptotic convergence of the chain towards the correct target distribution.

This algorithm, with the $(2.38)^2\frac{\Sigma_n}{d}$ variance for the adaptive bit, the $(0.1)^2\frac{I_d}{d}$ variance for the fix part and $\zeta = 0.05$, leads to poor acceptance rates due to the shape of the posterior distribution of our parameters and for some seasons, "learning" problems as the fix bit at the start of the chain does not lead to any valid values. For this we used an improvement of the algorithm [44]. By adding another adaptive element, to "learn" the scale of the proposal in order to lead to an acceptance rate close to the theoretical 0.234 value. For this we define:

$$Q'_n(x,.) = (1-\zeta)\mathcal{N}\left(x, \xi_n\Sigma_n\right) + \zeta\mathcal{N}\left(x, \Delta\right) \tag{47}$$

Parameter	Description	Dimension	
		Original	Final
m^+_{ij}	Number of individuals with detectable influenza among the total number of people consulting for ILI in age group i in week j	260	-
ϵ_i	Ascertainment probability of an infectious individual	5	3
ψ	Probability to become infected outside of the main epidemic	1	1
q	Transmissibility of the virus	1	1
σ_i	Susceptibility in age group i due to pre-existing immunity	5	3
l	Initial infectious seed for the epidemics	1	1
$\mathcal{A}$	Set of participants representative of contacts leading to transmission in the population	-	-

Table S6: Parameters to infer for each of the 14 seasons and 3 strains.

where Δ is a suitably chosen fixed diagonal matrix and ξ_n is updated as follows:

$$\begin{cases} \xi_{n+1} \leftarrow \xi_n - 0.234 * \delta_n, & \text{if the proposal is rejected,} \\ \xi_{n+1} \leftarrow \xi_n + 0.766 * \delta_n, & \text{if the proposal is accepted,} \end{cases} \tag{48}$$

with $\lim_{n\to\infty} \delta_n = 0$ to keep the Diminishing Adaptation property. The use of this adaptive scaling factor allows the algorithm to learn the optimal scaling for the proposal distribution and gives in practice very good results.

From an implementation point of view, to avoid storing the whole history of the chain $\{\Theta^k\}_{1\leq k\leq n}$ with n potentially very big (several millions for long chains), we use that, as Σ_n^{ij} the element at row i and column j is equal to:

$$\begin{aligned} \Sigma_n^{ij} &= \frac{1}{n-1}\sum_{k=1}^{n}\left(\Theta_{ij}^k - \bar{\Theta}_{ij}\right)\left(\Theta_{ji}^k - \bar{\Theta}_{ji}\right) \\ &= \frac{1}{n-1}\sum_{k=1}^{n}\left[\Theta_{ij}^k\Theta_{ji}^k - \Theta_{ij}^k\bar{\Theta}_{ji} - \Theta_{ji}^k\bar{\Theta}_{ij} - \bar{\Theta}_{ij}\bar{\Theta}_{ji}\right] \\ &= \frac{1}{n-1}\sum_{k=1}^{n}\left[\Theta_{ij}^k\Theta_{ji}^k\right] - n\bar{\Theta}_{ij}\bar{\Theta}_{ji}, \end{aligned} \tag{49}$$

we only need to store at each step the matrix $\sum_{k=1}^{n}\left[\Theta^k(\Theta^k)^T\right]$ and $\bar{\Theta} = \frac{1}{n}\sum_{k=1}^{n}\Theta^k$. Both are easily stored and updated on the fly.

Coding methodology To reduce the chances of coding errors, the functions used in the inference algorithm have been coded using three parallel routes:

1. the built-in statistical functions available in R [35],
2. "fast" functions using the algorithms presented in this supplementary document implemented in R,
3. these "fast" functions implemented in C.

For each functions, the results from the "fast" algorithms coded in R were compared with the results from the built-in functions from the R package (usually being the same up to a constant). Then the results from the C code were compared to the results given by the "fast" functions implemented in R. The parallel development of three codes, though more time consuming, reduces the chances of coding errors.

The C code for the transmission model and the inference algorithm are available from the corresponding author on request.

Performance of the algorithm The inference presented in this work has been produced using 11 million length chains (1 million for the burn-in), with a thinning of 10,000. This took $\approx$ 4 hours on a cluster for the 14 seasons and 3 subtypes of the study.

Performance of the algorithm on simulated data Additionally, we checked whether we could retrieve parameters from simulated epidemics using associated (simulated) data. Inputting the simulated data, the inference algorithm managed to estimate the correct values and propagate the corresponding uncertainty associated with the sample scheme adopted to generate the mock sets of data. As in any Bayesian analysis though, the accuracy of the resulting posterior to retrieve the "true" values is a combination of the quality of the data and the assumptions made for the prior estimation. Strong informative priors distant from the "true" values will bias the estimates. This is true for the real valued parameters but also for the inferred structure of contacts.

To illustrate this and demonstrate how our inference algorithm works on simulated data, we have chosen to present the following example. We simulated an epidemic peaking at a similar time than the 1997/98 season for H3N2. We assumed that the background of ILI (from different pathogens) mirrors the epidemiology of the influenza virus considered (the simulated influenza epidemic is thus assumed to be at a constant 10% of the total ILI). The value of the ascertainment probabilities and transmissibility are chosen near the median of the prior distribution. We assumed a non informative prior on the susceptibility parameters and a contact matrix distant from the POLYMOD mean matrix (the algorithm infers which contacts are likely to describe best the epidemics and uses the POLYMOD data for this). The results of the inference can be seen in Figure S6 with the "true" values marked with a dashed line (panels **C**, **D** and **E**) and the posteriors in red. The contact matrix resulting from the "true" set of resampled contacts used to simulate the epidemic and the mean POLYMOD matrix are represented for comparison in panel **B** of the figure.

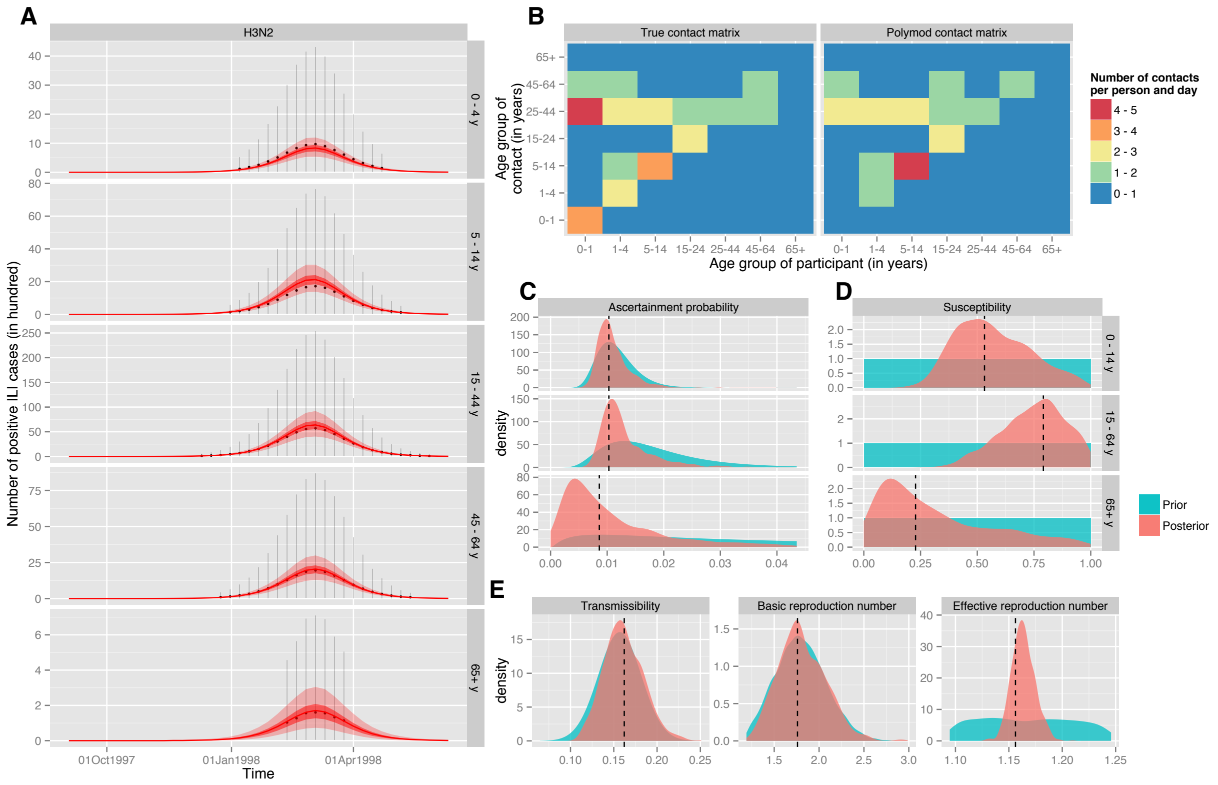

Figure S6: Inference results from a simulated dataset. **A**: Comparison of the fit of the model to the age-specific time-series of positive ILI cases (m^+_{ij}) generated from the simulated epidemic. For the model, the mean (red line) with 50 and 95% CI (shaded areas) for m^+_{ij} are based on the binomial process $m^+_{ij} \sim Binom(Z^{\theta}_{ij}, \epsilon_i)$. For the "data", since $n^+_{ij} \sim HyperGeo(n_{ij}, m^+_{ij}, m_{ij})$, the unbiased estimator for m^+_{ij} is $n^+_{ij} \times m_{ij}/n_{ij}$ (black dots) whereas its 95% CI can be computed using the R function hypergeo.conf (error bars). **B**: Comparison of the contact matrix used to simulate the data (left panel) to the Polymod contact matrix resulting from the contact survey used as a prior for the structure of contact in the population (right panel). **C**: Age-specific probability of being recorded as ILI and positive if tested and infected (ϵ_i). **D**: Age-specific susceptibility at the begining of the flu season (σ_i). **E**: Transmission coefficient (q, left panel) and derived quantities: basic (R_0, middle panel) and effective ($R_e(t = 0)$, right panel) reproduction numbers. For **C** $-$ **E**: The prior distribution is shown in blue and the posterior distribution in red.

Results show that the algorithm is "learning" from the data. The algorithm is able to estimate the parameters correctly, in particular the ones with no prior information (initial susceptibility). Also, the correlations between susceptibility and

ascertainment probabilities in a given age group appears clearly together with the "banana" shape of the likelihood function (see Figure S7).

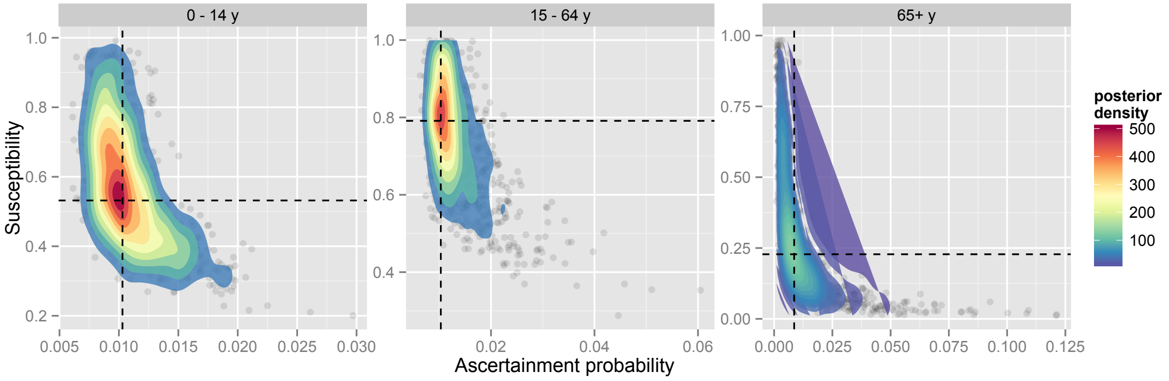

Figure S7: Values (posterior distributions) of the ascertainment probabilities and susceptibility for age groups 0-14 year old, 15-44 year old and 65+ year old. The dashed lines indicate the position of the "true" values used to generate the data.

However, the actual epidemic curves underestimate the number of cases in the 0-4 year old children and overestimate the infections among the 5-14. As the two age group share the same ascertainment probability and the same susceptibility profile, the estimation relies on the contact matrix to adjust the mixing accordingly to the patterns observed in the data. A closer examination of (for example) the posterior distribution of the dominant eigen values of the inferred contact matrices (used as a summary statistics) shows that the resulting distribution "moves" away from the prior towards the "true" value but still largely covers the prior spectrum of values (Figure S8).

If, for the same dataset, the priors (apart the one on the contacts) are tighten towards their "true" values, then the inferred matrices move towards the "true" matrix. It is illustrated in Figure S9 where the posterior of the dominant eigen values can be seen moving toward its "true" value.

Tests of the algorithm on simulated data suggest that the algorithm is able to infer correctly both real valued parameters with their uncertainty and correlation structure, but also the structure of relevant epidemiological contacts inside the population.

2.4 Comparison of our statistical framework with other approaches

Our study is the first to combine, at the scale of 14 seasons and for the three subtypes circulating in human populations, an evidence synthesis with dynamical modelling. We utilize this analysis to assess the impact of a change of vaccination policy. It takes into account the health care scope by including the risk structure of the population as well as the age mixing. One of the most difficult parts of such a project is to propose a coherent framework to combine rigourous statistical inference with dynamical modelling.

The main difficulty of the statistical modelling is to manage to link accurately the set of infectious individuals (used in the transmission model) with the set of people recorded by GP as presenting with ILI (the observation variables). In simple words, one needs to assess how many of the cases recorded by the GPs as ILI are actually consulting following infections by influenza viruses. The observation process can be seen as the intersection of sets (see Venn diagram in Figure 2 of the main paper). The accurate linkage of these two quantities is the keystone of the approach and is performed in our study by the following model:

$$\begin{cases} m_{ij}^{+} \sim \mathcal{B}(z_{ij}^{\theta}, \epsilon_i) + \mathcal{P}(\psi \epsilon_i N_{ij}^{mon}) \\ n_{ij}^{+} \sim \mathcal{H}(n_{ij}, m_{ij}^{+}, m_{ij}) \end{cases} \quad (50)$$

This equation mechanistically describes the drawing process of sampling and thus represent as faithfully as possible the observation process. The inclusion of an hypergeometric distribution makes the numerical implementation of the problem computationally challenging. Our approach to implement our model is described in Section 2.3.

To circumvent the difficulties of its implementation, it could be tempting to approximate the hypergeometric distribution with a simpler distribution such as the binomial distribution. For the approximation to work well, the size of the sample should be much smaller than the population from which it is drawn and the probability of success must not be too close to 0 or 1. Unfortunately, under typical surveillance-oriented sampling, these conditions are constantly violated.

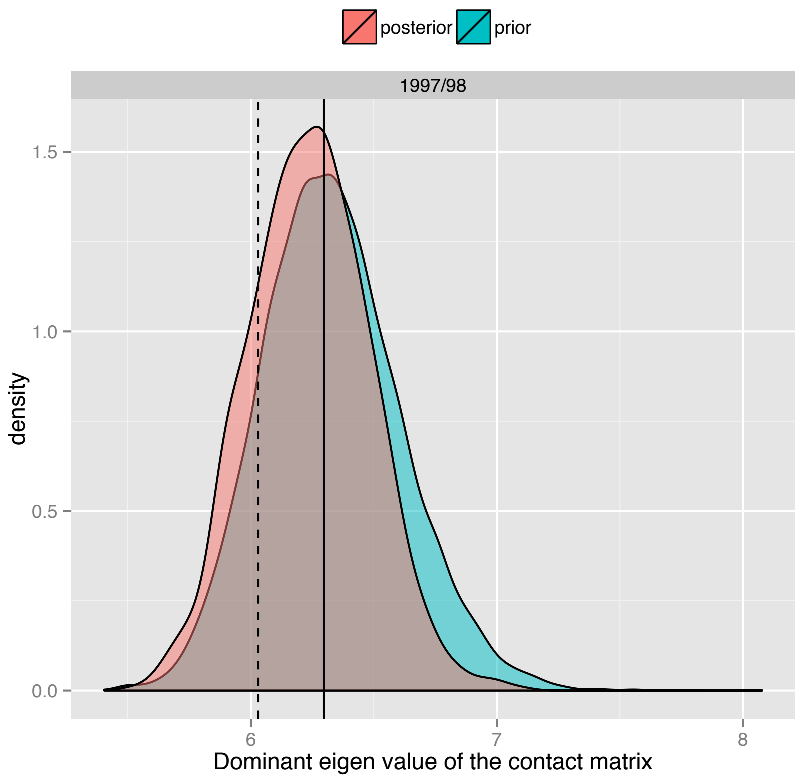

Figure S8: Prior (blue) and posterior (red) distributions of the dominant eigen values of the contact matrices. The solid vertical line represents the mode of the distribution of the eigen values of the contact matrices obtained by resampling the POLYMOD data. The dashed line represents the 'true' eigen value of the contact matrix used to generate the mock data set.

For example, at the beginning of the epidemics when ILI cases are sparse in the population monitored and surveillance high, the number of virological samples can be close to the number of people consulting. If other pathogens are low, the positivity might be high in the course of the epidemics. The binomial approximation is likely to introduce biases, especially during the beginning of the epidemics, the period on which the estimation of R_0 is highly dependent.

Two recently published studies (both with pandemic data) present the objective of coupling statistical inference from health care associated data and dynamical models [45, 46]. Both have adopted a binomial approximation which has lead these studies to add further layers to enable the inference (negative binomial distributions to introduce overdispersion, strong priors on some of the paramters, etc.).

In the first study, Birrell et al. [45] combined age-specific daily ILI, virological, confirmed cases and serological data collected during the 2009 influenza pandemic in London. Thus, in comparison with our study, the authors benefited of two additional type of data as well as a finer temporal resolution. Focusing only on the type of data we have in common, Birrell et al. [45] assume that the ILI (m_{ij}) and the virological (n_{ij}^{+}) data are generated as follows:

$$\begin{cases} m_{ij}^{+} = p_{ij}^{GP} \sum_{k=0}^{j} p^{sympt} z_{i,j-k}^{\theta} f_{lag}(k) \\ m_{ij} \sim neg\mathcal{B}\left(m_{ij}^{+} + m_{ij}^{-}, \eta_j\right) \\ n_{ij}^{+} \sim \mathcal{B}\left(n_{ij}, \frac{m_{ij}^{+}}{m_{ij}^{+} + m_{ij}^{-}}\right), \end{cases} \tag{51}$$

where m_{ij}^{-} is the age-specific daily number of non-flu ILI cases (background consultation), η_j is a dispersion parameter, p^{sympt} the probability, given infection, to become symptomatic, p_{ij}^{GP} the probability, given symptomatic infection, to consult a GP and be recorded as ILI (age and time dependent probability and the lag between infection and GP report, which is assumed to be gamma distributed with fixed mean ($\mu_{lag} = 4.1$ days) and standard deviation ($\sigma_{lag} = 3.5$ days) where $f_{lag}(k)$ gives the probability that the gamma random lag lies in the interval $[k, k+1[$.

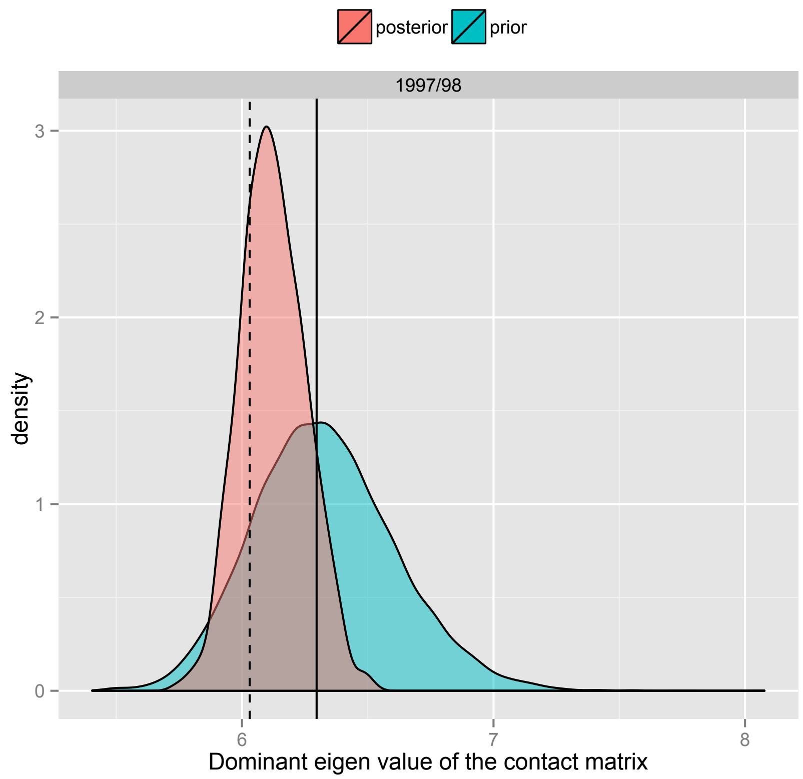

Figure S9: Prior (blue) and posterior (red) distributions of the dominant eigen values of the contact matrices. The solid vertical line represents the mode of the distribution of the eigen values of the contact matrices obtained by resampling the POLYMOD data. The dashed line represents the "true" eigen value of the contact matrix used to generate the mock data set.

Thus, in addition to the choice of the binomial for the virological sampling, Birrell's model differs from ours as a deterministic approach for the mean number of people consulting among the infected combined with a negative binomial integrating the background consultation rate. The deterministic part of the process includes a time lag in ILI reporting by GP. Though we could have included it in our model, little effect is expected on weekly aggregated ILI data as the time-scale of this lag is likely to be less than one week. Thus, in our context, the computationally expensive evaluation of this equation (as noted by Birrell et al. [45]) doesn't appear fully justified.

This model is coupled with strongly informative priors on the m^-_{ij}. For this, the background consultation rate (m^-_{ij}) in London is assumed to follow the relation:

$$\log(m^-_{ij}) = \alpha_j + \beta_i + \gamma_{ij} \tag{52}$$

where the time (α_j), age (β_i) and interaction (γ_{ij}) terms are obtained by regressing this log-linear model (52) on empirical estimates of $\widehat{m}^-_{ij} = (1 - n^+_{ij}/n_{ij})m_{ij}$ obtained from the regions outside London. More precisely, the log-linear regression provides means and covariance matrix for these parameters, which are then used as priors in the Bayesian framework of Birrell et al. [45].

The second paper, by Dorigatti et al. [46], combined age-specific weekly ILI data with weekly virological data collected during the 2009 influenza pandemic in Italy. In contrast to our study, the weekly virological data were only available at the population level. Put another way, the authors had to use the age-aggregated weekly number of tested ($\bar{n}_j = \sum_i n_{ij}$) and positive ($\bar{n}^+_j = \sum_i n^+_{ij}$) samples.

Dorigatti et al. [46] introduce the weekly true positivity in the entire ILI population: $\pi_j = \frac{\sum_i m^+_{ij}}{\sum_i m_{ij}}$ and assume that m^+_{ij} and $\bar{n}^+_j$ are generated by the following processes:

$$\begin{cases} m_{ij}^{+} \sim \mathcal{B}(z_{ij}^{\theta}, \epsilon_i), \\ \bar{n}_j^{+} \sim \mathcal{B}(\bar{n}_j, \pi_j) \\ m_{ij}^{-} \sim neg\mathcal{B}(m_{ij}^{+}, 1-\pi_j), \end{cases} \tag{53}$$

The use of the negative binomial to generate the m_{ij}^{-} is mathematically convenient as it allows to use a conjugate beta prior to simplify the calculation of the associated likelihood. It is nevertheless not appropriate in terms of representation of the sampling mechanism, and, as noted by Dorigatti et al. the process can generate situations where the inferred m_{ij}^{-} are bigger than the monitored populations.

3 Supplementary results

3.1 Inference for 14 seasons and 3 subtypes

3.1.1 Season 2003/04 with serology

Figure S32 gives the serological data for the 2003/04 season. Reasonable convergence of the Markov chain was achieved and the model gives sensible best-fitting parameter estimates. For instance, the best fit gives a value of $R_0 = 1.8$ and $R_e(t = 0) = 1.35$, which are consistent with other estimates for influenza (e.g. Ferguson et al. 2005). The transmissibility q is estimated as 0.165 with a 95% credible interval of [0.132, 0.205]. We thus have chosen for the other years a normal prior where q follows a normal distribution $\mathcal{N}(0.165, 7.69 \times 10^{-4})$ distribution (so the prior of the other year have the same mean and a standard deviation 1.5 times bigger than the posterior of the 2003/04 season in H3N2).

3.1.2 Other seasons

Figures S10 to S51 compare the fit of the model to the age-specific swabbing data (left 4 panels), and show posterior distributions for some of the estimated model parameters, and the derived quantities R_0 and R_e (bottom row), for H3N2, H1N1 and influenza B and each of the year of the period. The comparison of the predicted positivity with the observed one suggests that the model is able to capture the timing and magnitude of the strain-specific epidemic peaks, as well as the age-specific positivity. It also highlights the wide credibility intervals on the swabbing data, which are derived from relatively small sample sizes taken in any given week for any given age group.

In general, for a given strain, in years in which there were significant numbers of positive cases, then the expected pattern of susceptibility with age is observed, with higher levels of susceptibility in the younger age groups, declining into adulthood and the elderly (see for instance H3N2 1996/97, H1N1 2000/1 and B 2000/01). In these years, the effective reproduction number at the outset of the epidemic, (Effective reproduction number, middle of panel D) is estimated to be well above one, as expected. In years in which there were very few positive samples, the model cannot accurately distinguish between low numbers of initial seeds (which is related to low importations) with perhaps relatively high susceptibility, or higher number of seeds and lower susceptibility. Hence, in these years there are wide credibility intervals on the estimates of age-specific susceptibility (see for instance, H1N1 1996/97). However, even in these years very high levels of susceptibility are not well supported by the data, and the distribution for the reproduction number only just exceeds one. Examining the pattern of susceptibility over time for a given strain, it is clear that the model suggests that susceptibility is generally higher at the outset of an epidemic year, and lower in years in which there is no epidemic (see for instance, for influenza B, comparing the posterior distribution on susceptibility around the epidemic years of 1996/97, 2000/01 and 2005/06).

Estimates of the contact patterns (top right panel for Figures S10 to S51) are relatively tight and consistent from one year to the next and between strains; with contact within and between age group 3 (5-14) and other age groups being consistently estimated as the highest. The exception to this is the mixing within the first age class (under 1), in which there are very wide CIs on the underlying contact rate. This is due to a combination of very small sample size in the original POLYMOD dataset for this age group, and no age-specific epidemiological data (no swabs were taken from the under 1s).

3.1.3 Strain: H1N1

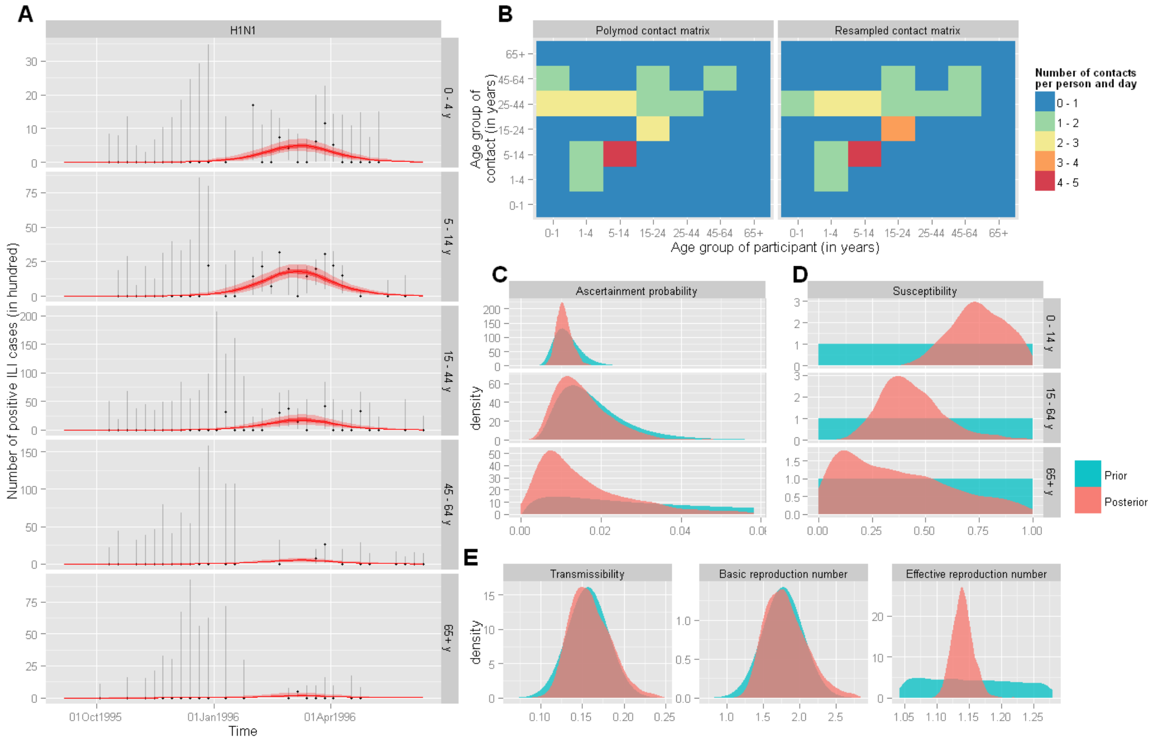

Figure S10: Inference results for H1N1 during the 1995-96 season. **A**: Comparison of the fit of the model to the age-specific time-series of positive ILI cases (m^+_{ij}) estimated from the data. For the model, the mean (red line) with 50 and 95% CI (shaded areas) for m^+_{ij} are based on the binomial process $m^+_{ij} \sim Binom(Z^\theta_{ij}, \epsilon_i)$. For the data, since $n^+_{ij} \sim HyperGeo(n_{ij}, m^+_{ij}, m_{ij})$, the unbiased estimator for m^+_{ij} is $n^+_{ij} \times m_{ij}/n_{ij}$ (black dots) whereas its 95% CI can be computed using the R function hypergeo.conf (error bars). **B**: Comparison of the contact matrix of the Polymod study (left panel) to the resampled matrix of the maximum likelihood MCMC sample (right panel). **C**: Age-specific probability of being recorded as ILI and positive if tested and infected (ϵ_i). **D**: Age-specific susceptibility at the begining of the flu season (σ_i). **E**: Transmission coefficient (q, left panel) and derived quantities: basic (R_0, middle panel) and effective ($R_e(t = 0)$, right panel) reproduction numbers. For **C** – **E**: The prior distribution is shown in blue and the posterior distribution in red.

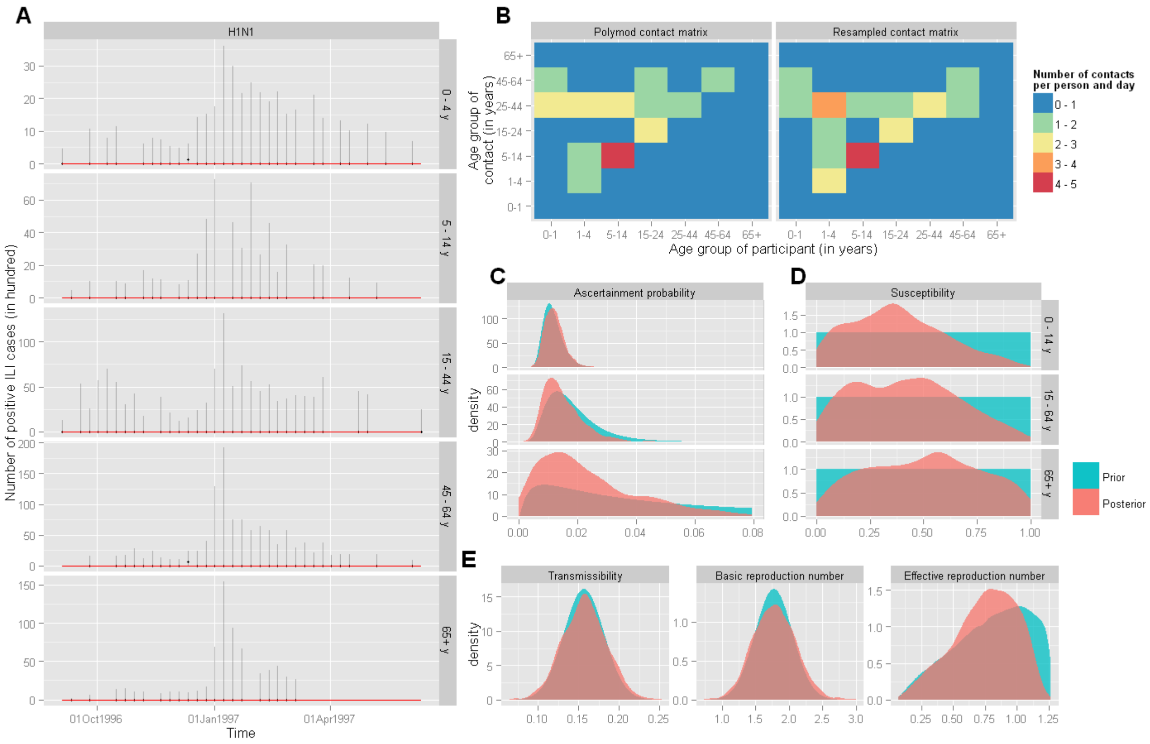

Figure S11: Inference results for H1N1 during the 1996-97 season. See the legend of the Figure S10 for more details.

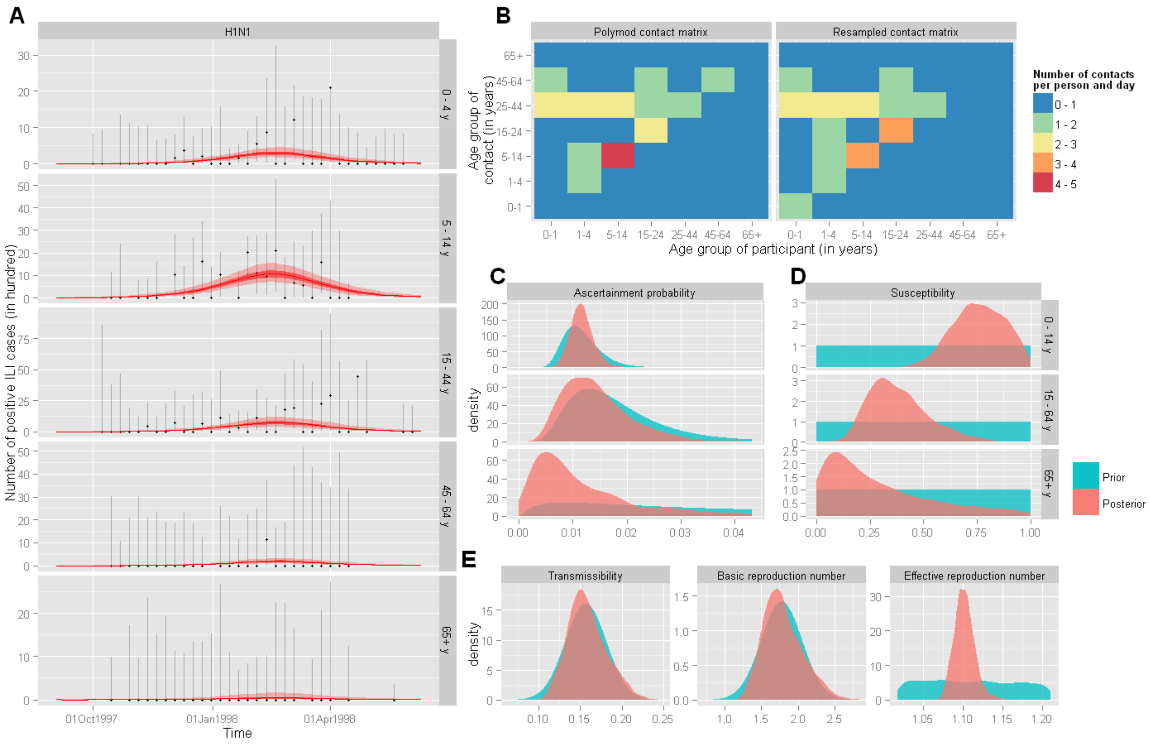

Figure S12: Inference results for H1N1 during the 1997-98 season. See the legend of the Figure S10 for more details.

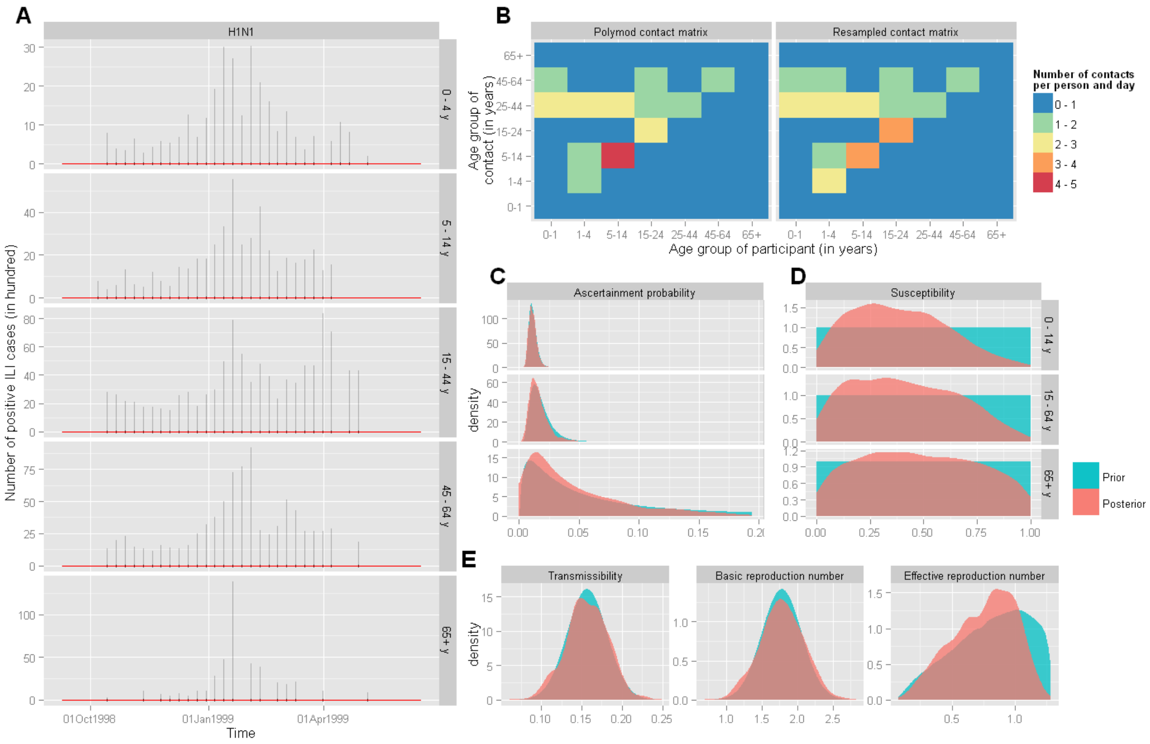

Figure S13: Inference results for H1N1 during the 1998-99 season. See the legend of the Figure S10 for more details.

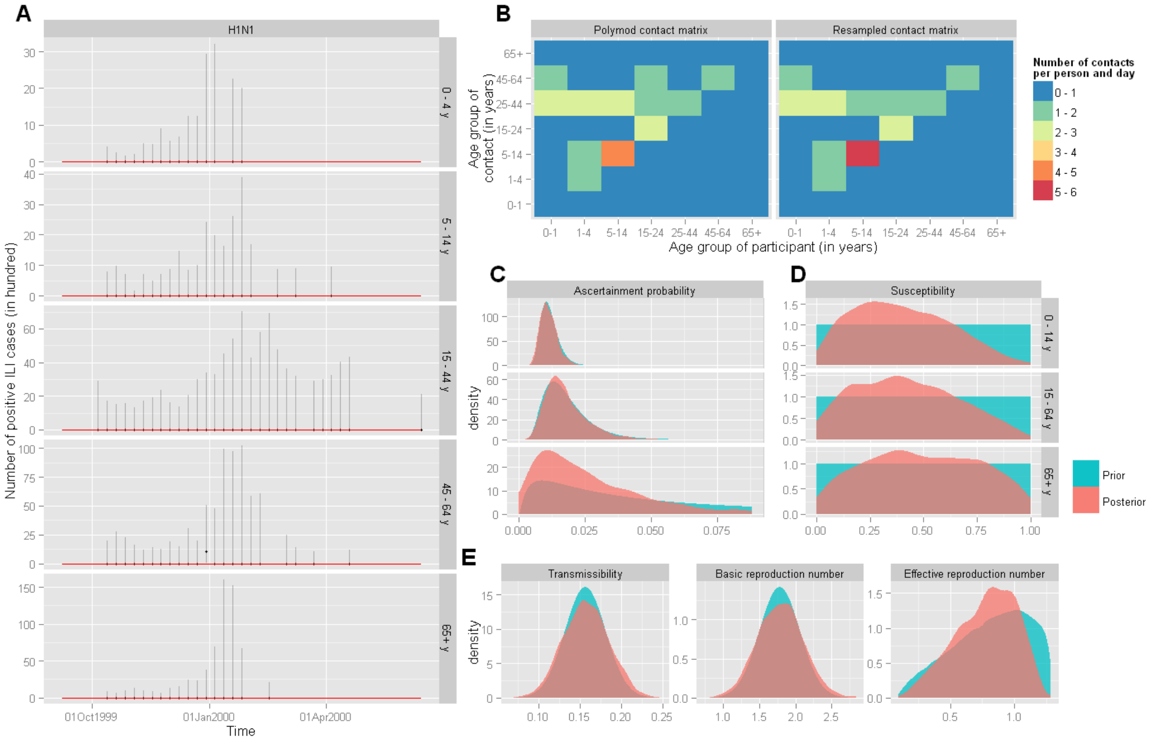

Figure S14: Inference results for H1N1 during the 1999-00 season. See the legend of the Figure S10 for more details.

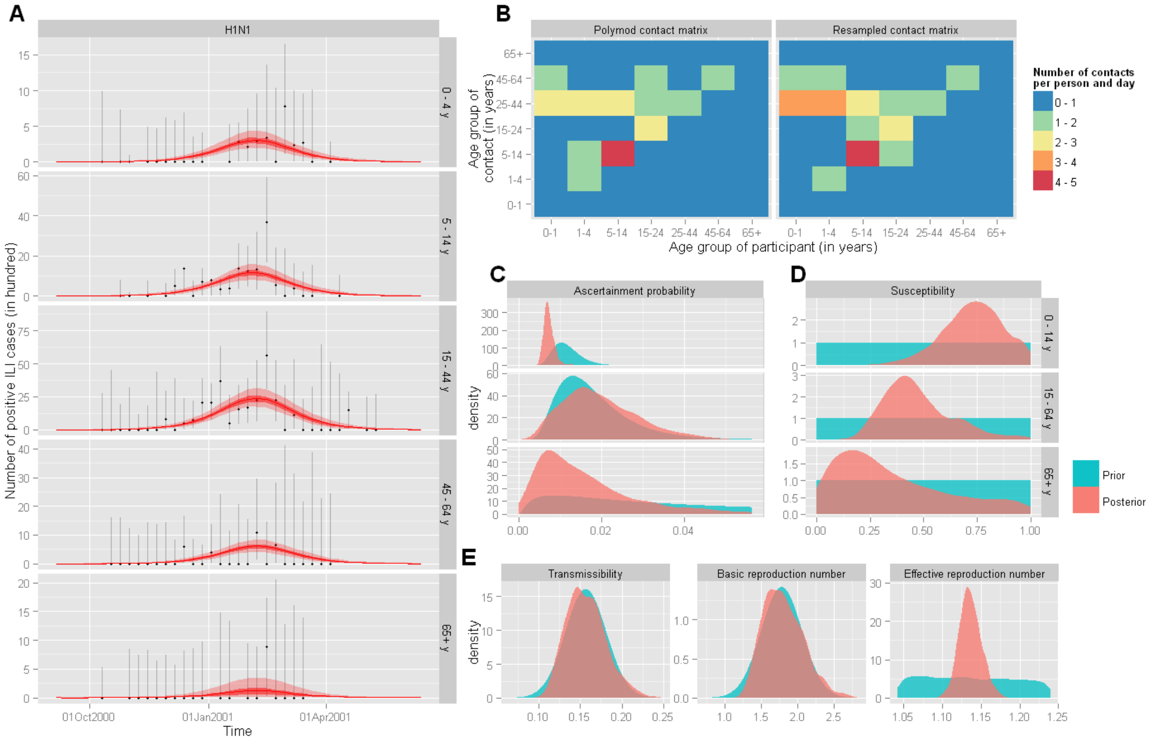

Figure S15: Inference results for H1N1 during the 2000-01 season. See the legend of the Figure S10 for more details.

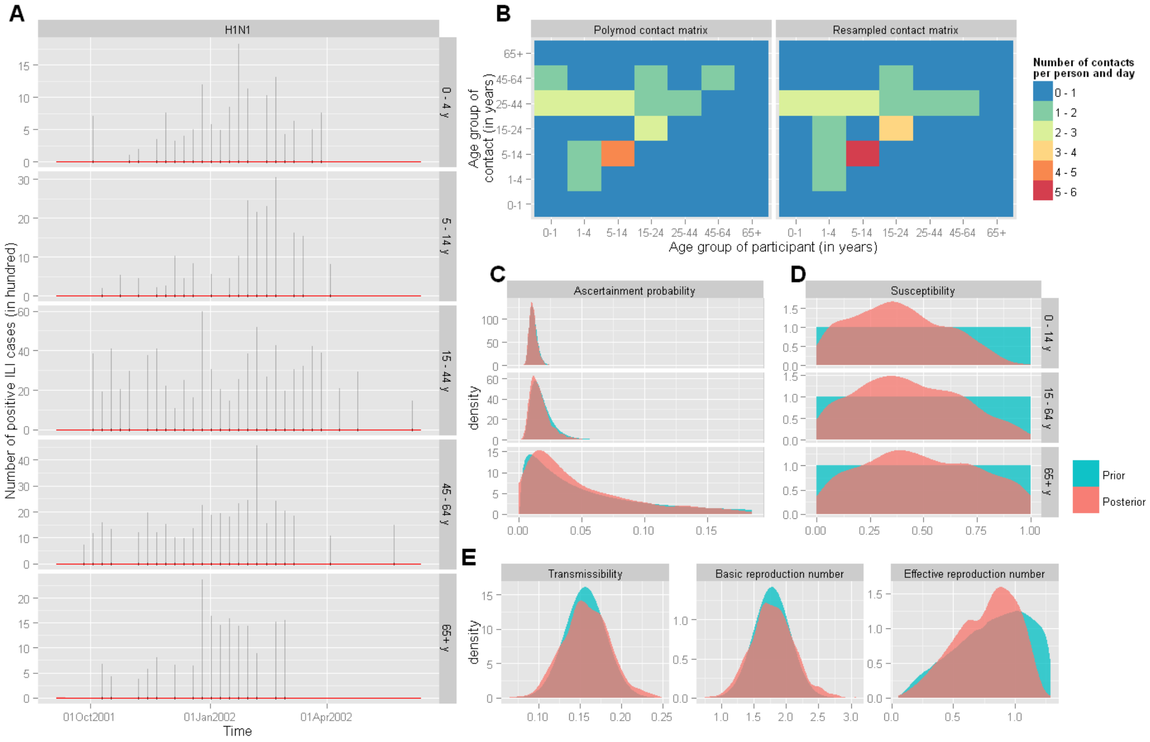

Figure S16: Inference results for H1N1 during the 2001-02 season. See the legend of the Figure S10 for more details.

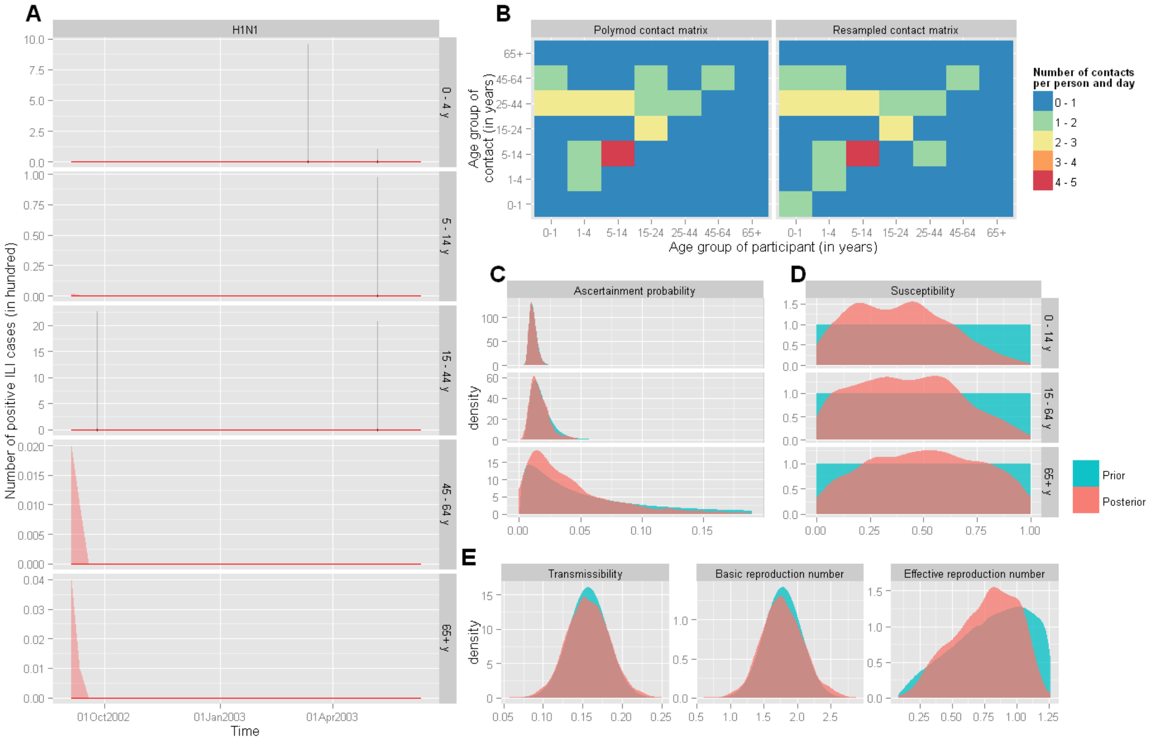

Figure S17: Inference results for H1N1 during the 2002-03 season. See the legend of the Figure S10 for more details.

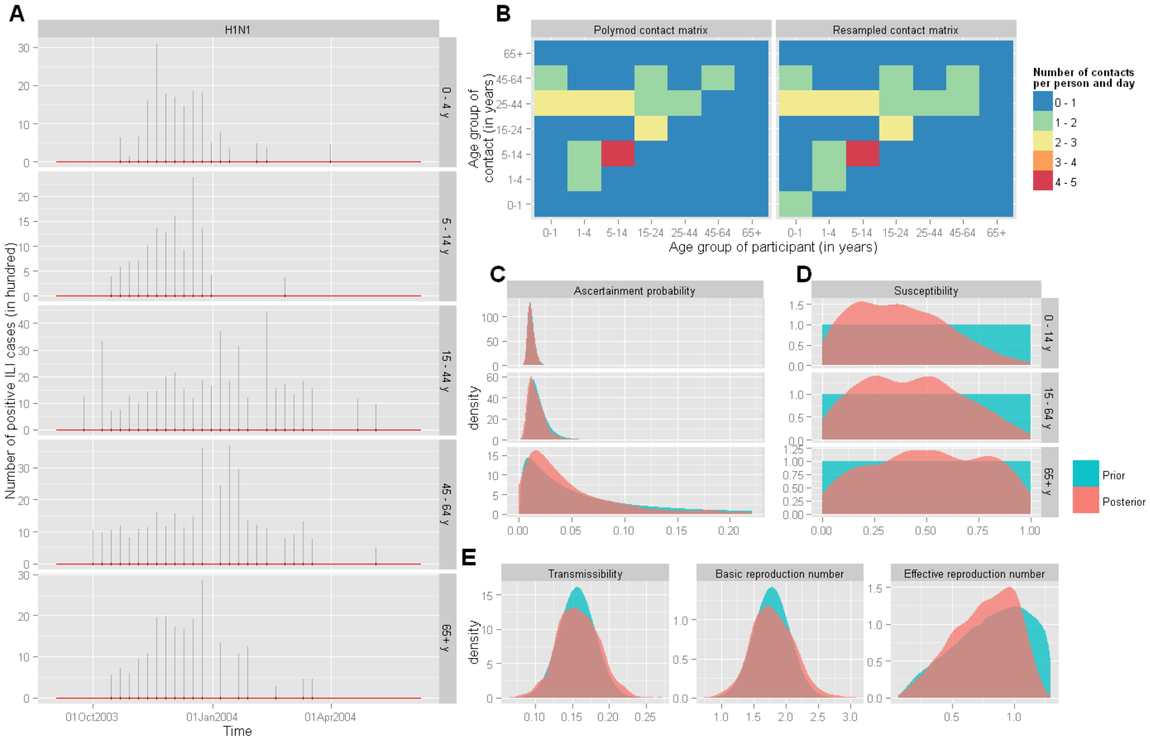

Figure S18: Inference results for H1N1 during the 2003-04 season. See the legend of the Figure S10 for more details.

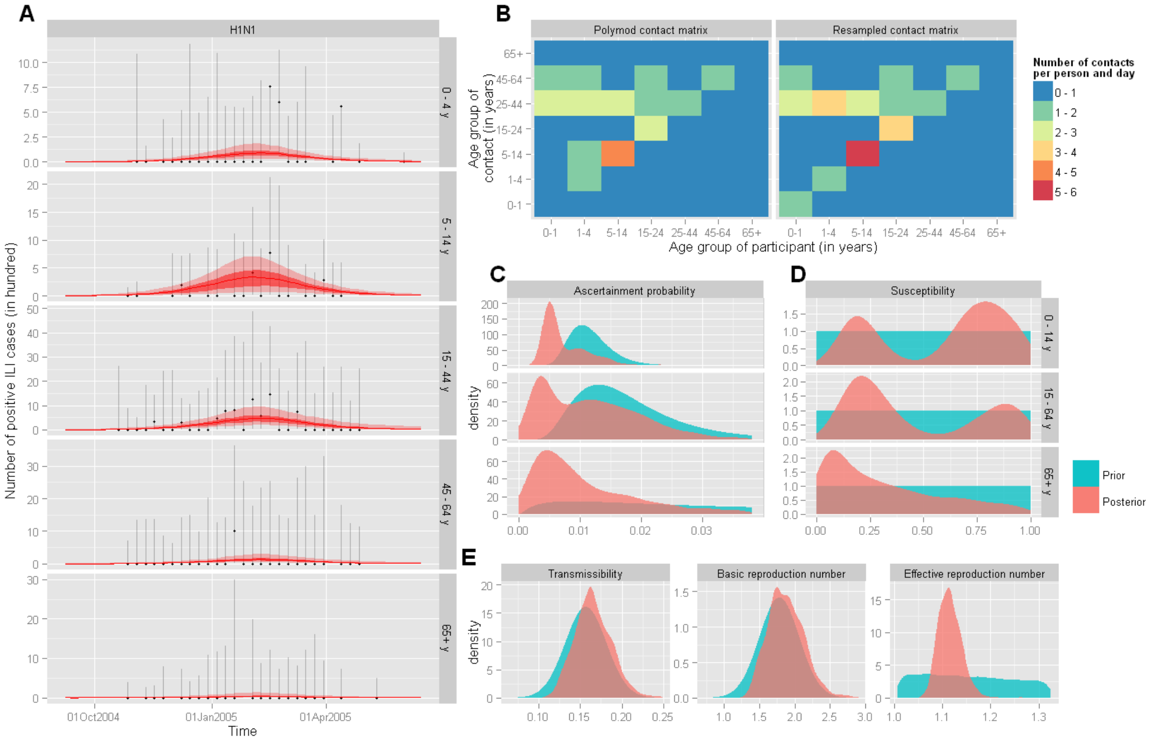

Figure S19: Inference results for H1N1 during the 2004-05 season. See the legend of the Figure S10 for more details.

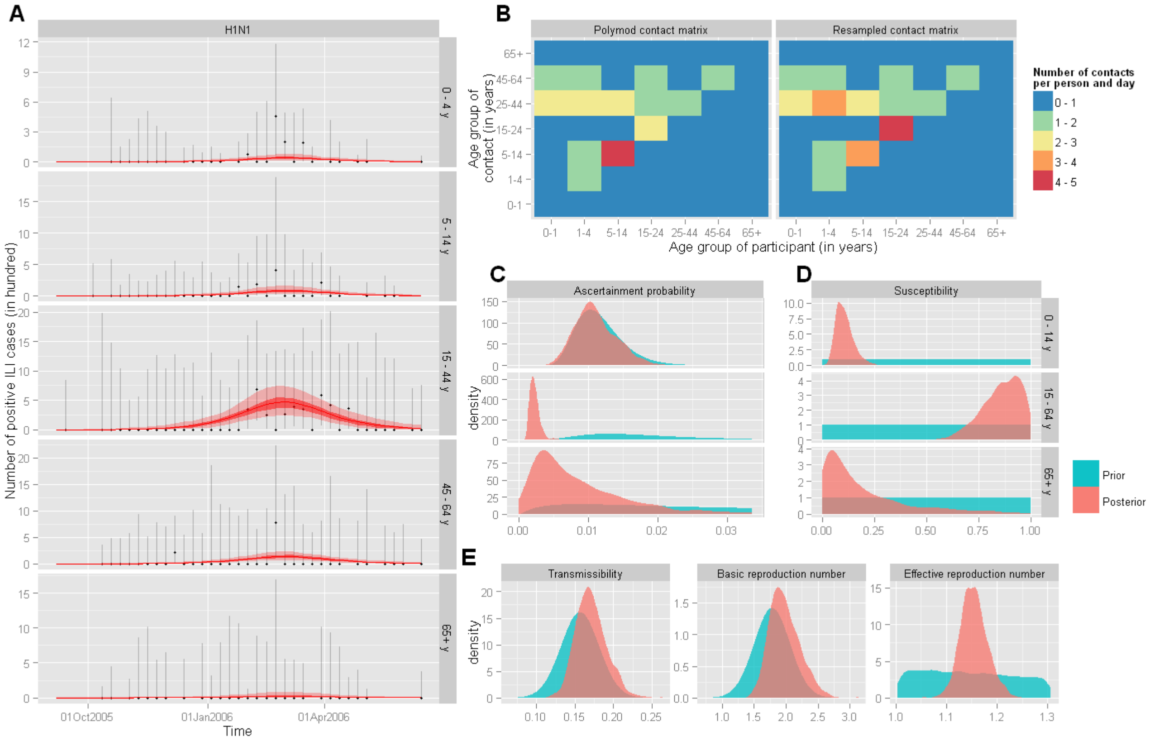

Figure S20: Inference results for H1N1 during the 2005-06 season. See the legend of the Figure S10 for more details.

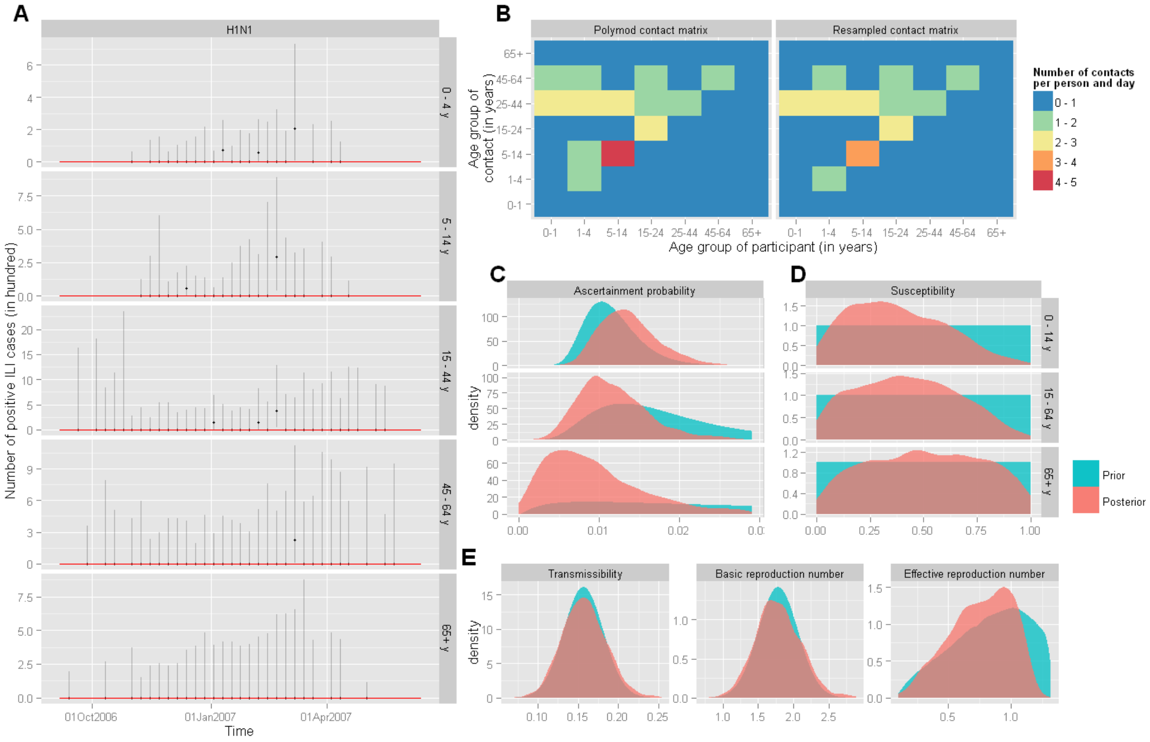

Figure S21: Inference results for H1N1 during the 2006-07 season. See the legend of the Figure S10 for more details.

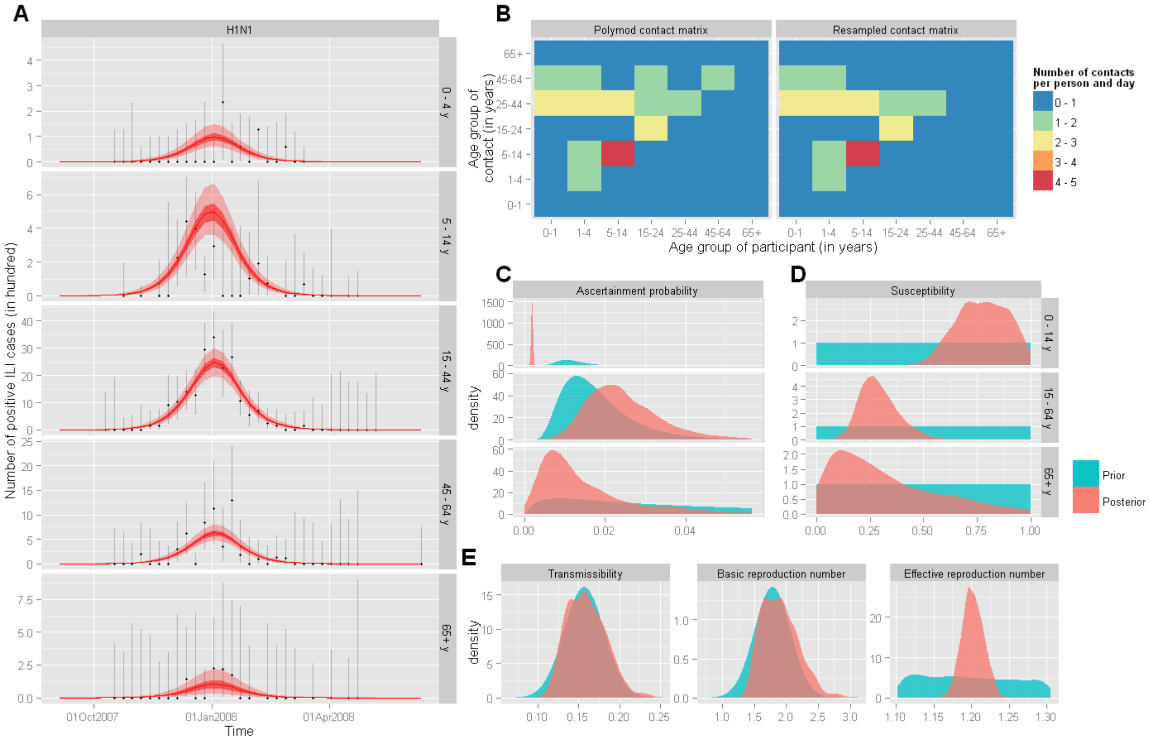

Figure S22: Inference results for H1N1 during the 2007-08 season. See the legend of the Figure S10 for more details.

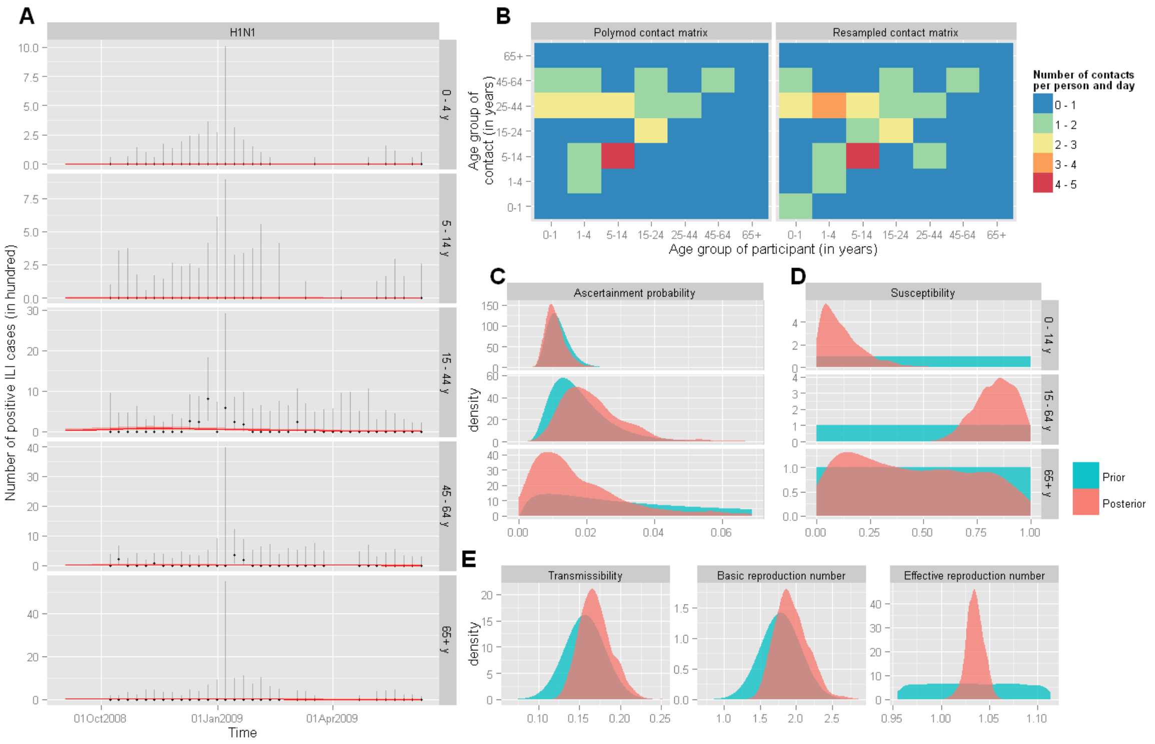

Figure S23: Inference results for H1N1 during the 2008-09 season. See the legend of the Figure S10 for more details.

3.1.4 Strain: H3N2

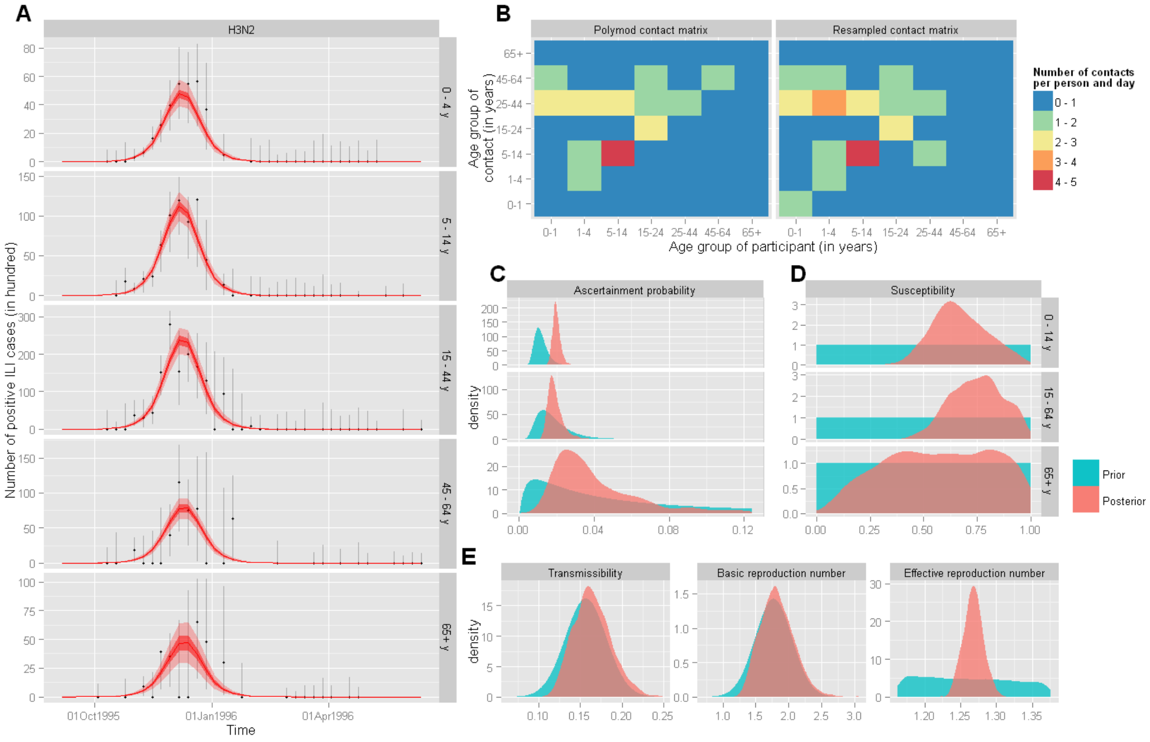

Figure S24: Inference results for H3N2 during the 1995-96 season. See the legend of the Figure S10 for more details.

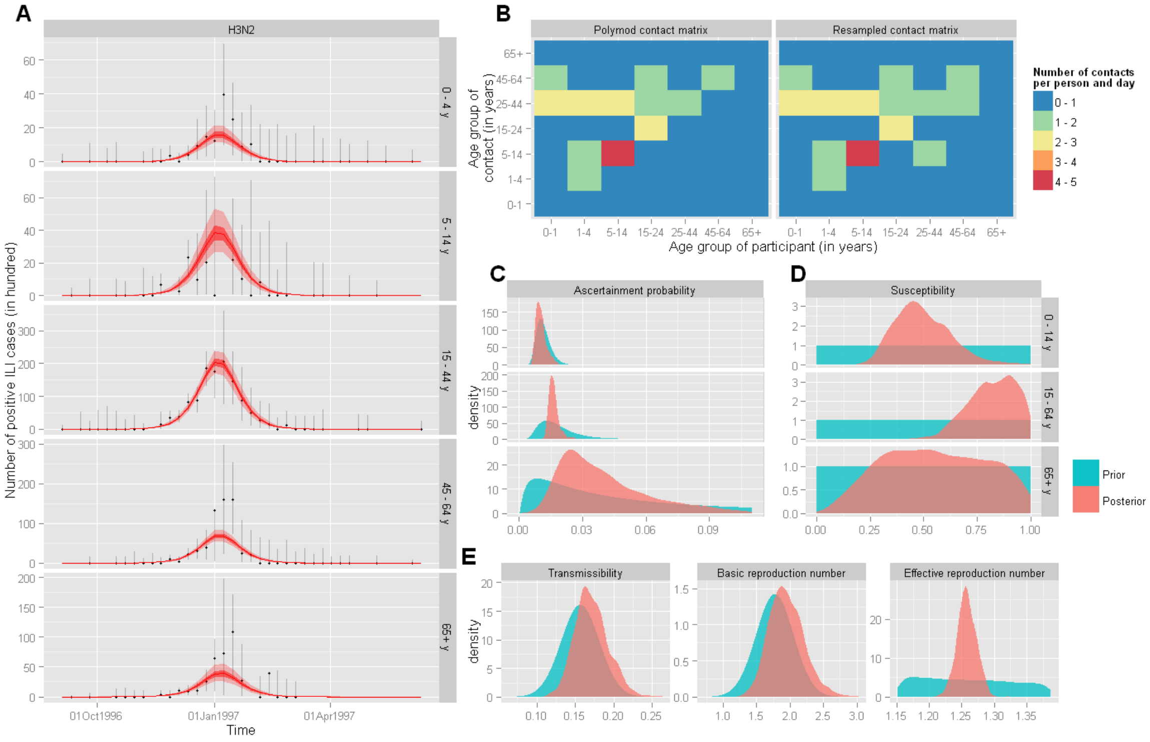

Figure S25: Inference results for H3N2 during the 1996-97 season. See the legend of the Figure S10 for more details.

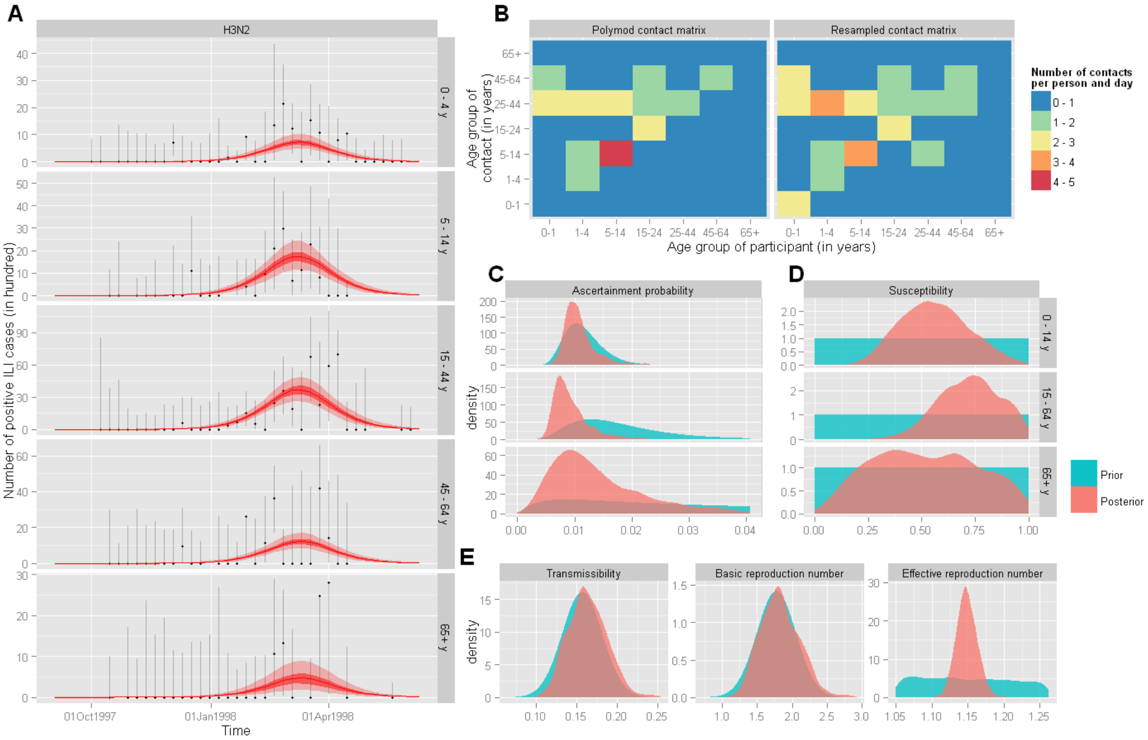

Figure S26: Inference results for H3N2 during the 1997-98 season. See the legend of the Figure S10 for more details.

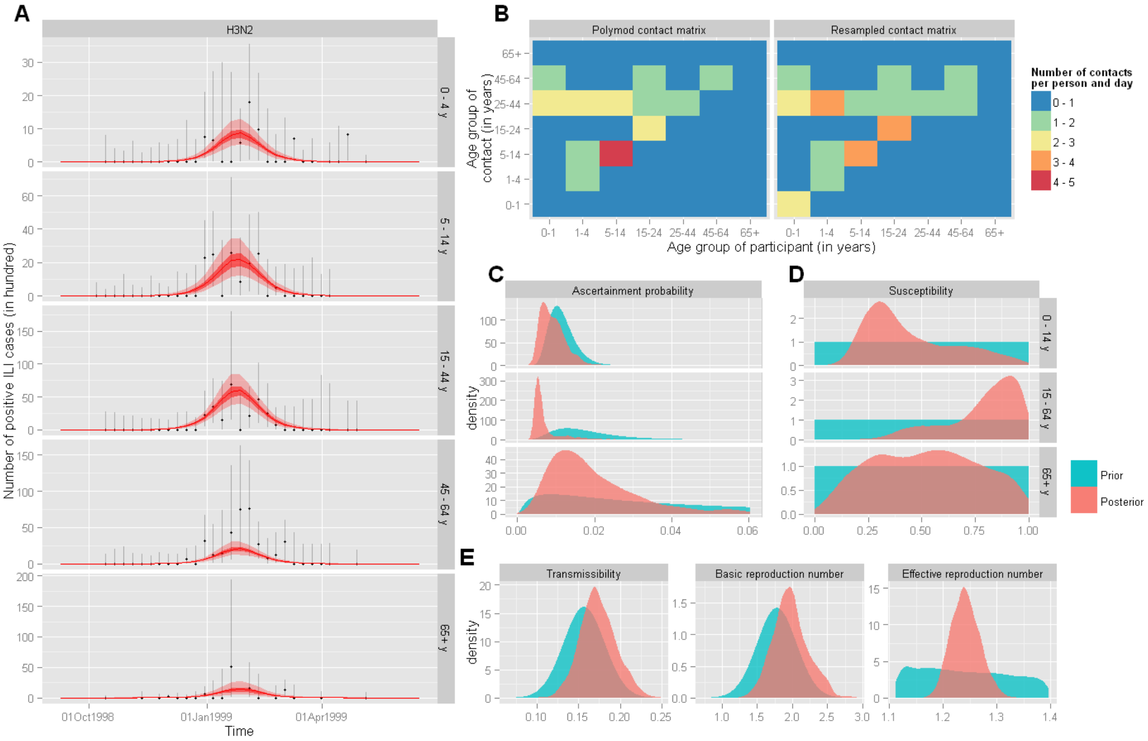

Figure S27: Inference results for H3N2 during the 1998-99 season. See the legend of the Figure S10 for more details.

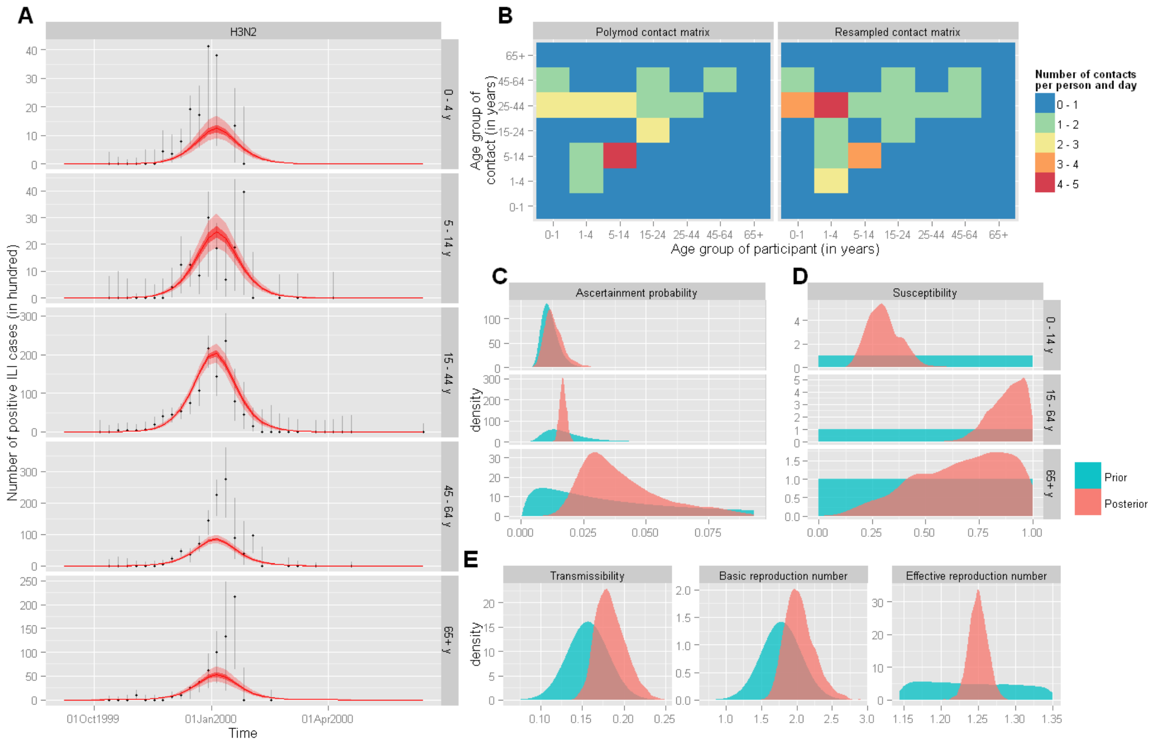

Figure S28: Inference results for H3N2 during the 1999-00 season. See the legend of the Figure S10 for more details.

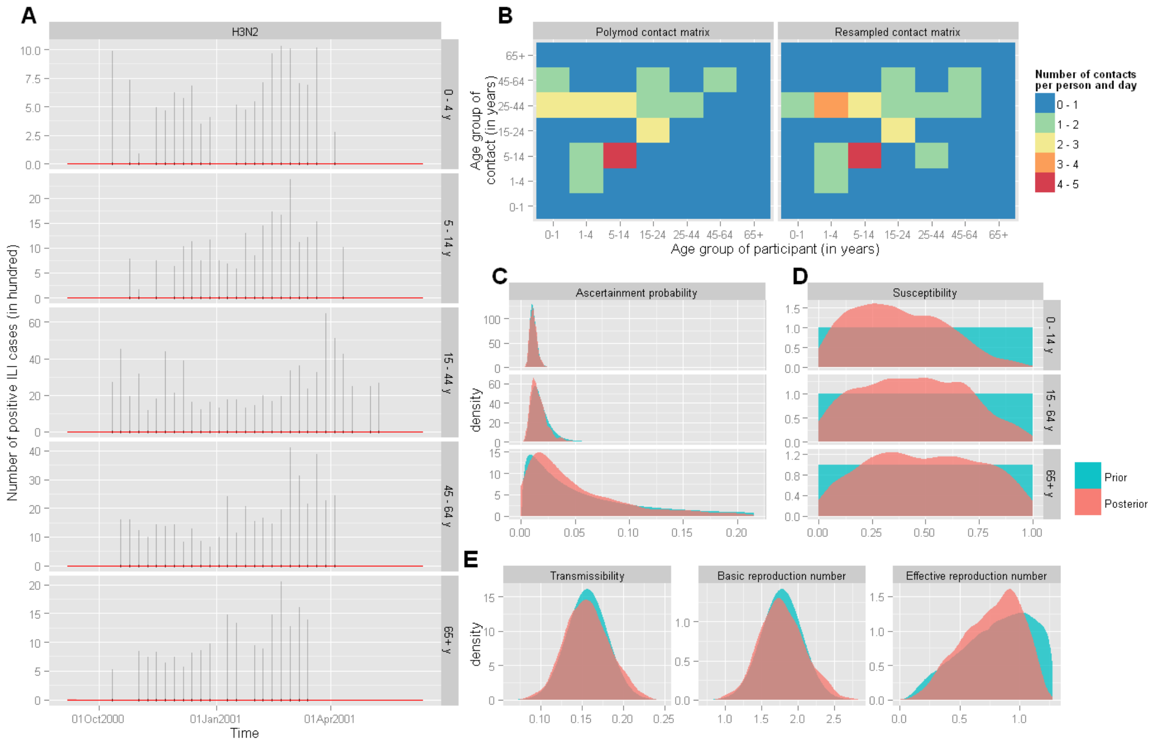

Figure S29: Inference results for H3N2 during the 2000-01 season. See the legend of the Figure S10 for more details.

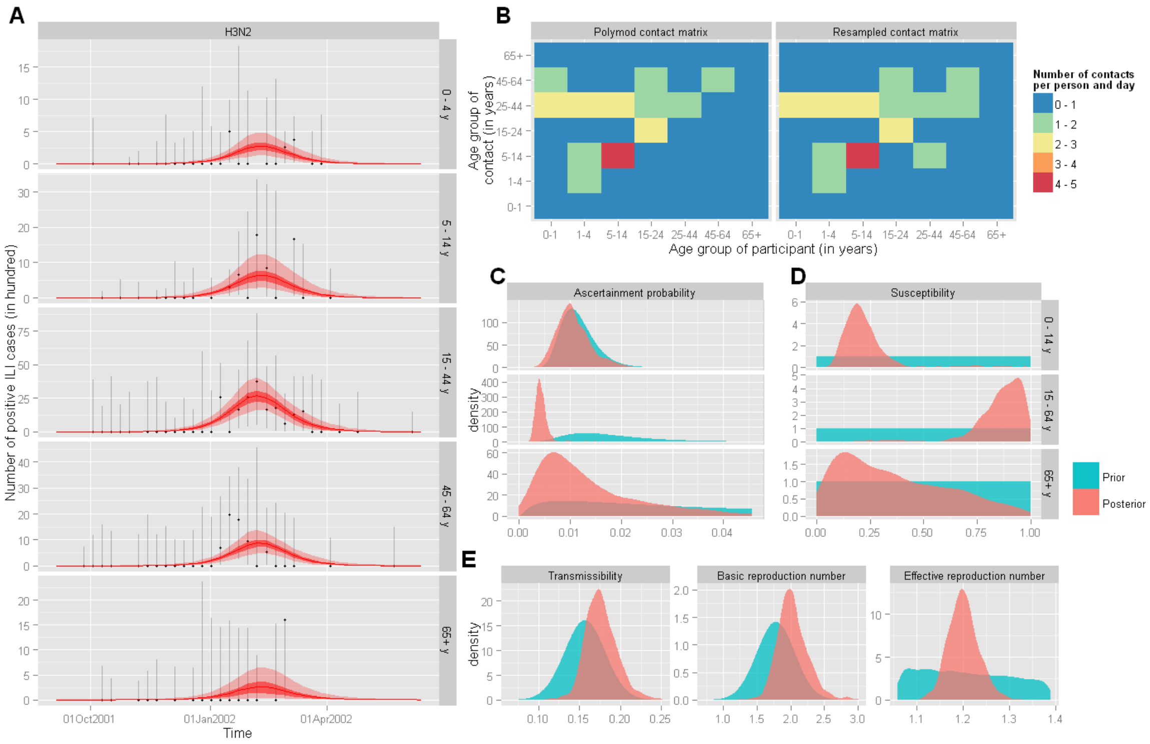

Figure S30: Inference results for H3N2 during the 2001-02 season. See the legend of the Figure S10 for more details.

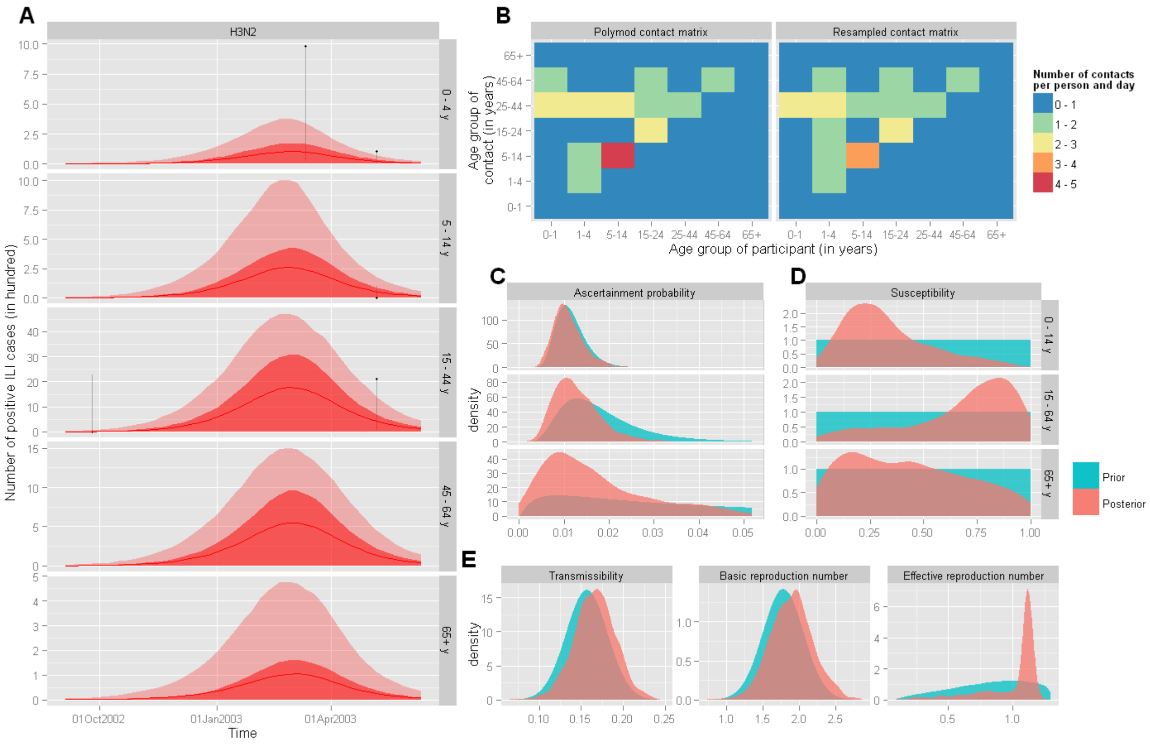

Figure S31: Inference results for H3N2 during the 2002-03 season. See the legend of the Figure S10 for more details.

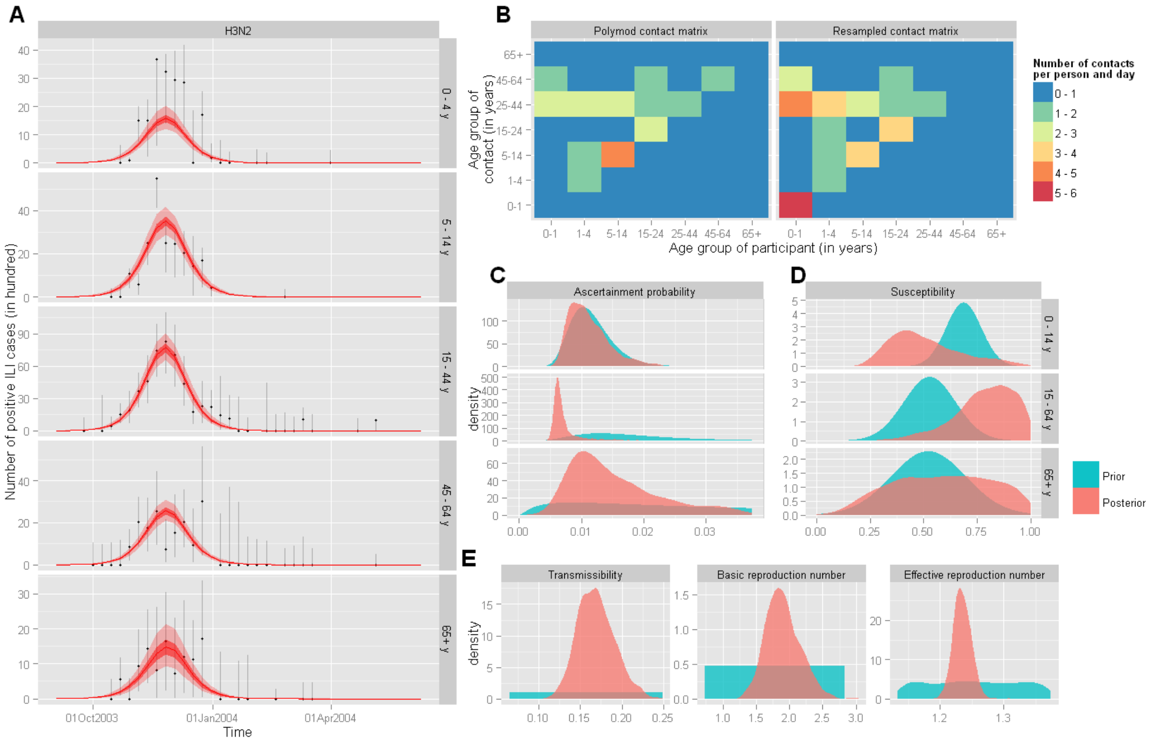

Figure S32: Inference results for H3N2 during the 2003-04 season. See the legend of the Figure S10 for more details.

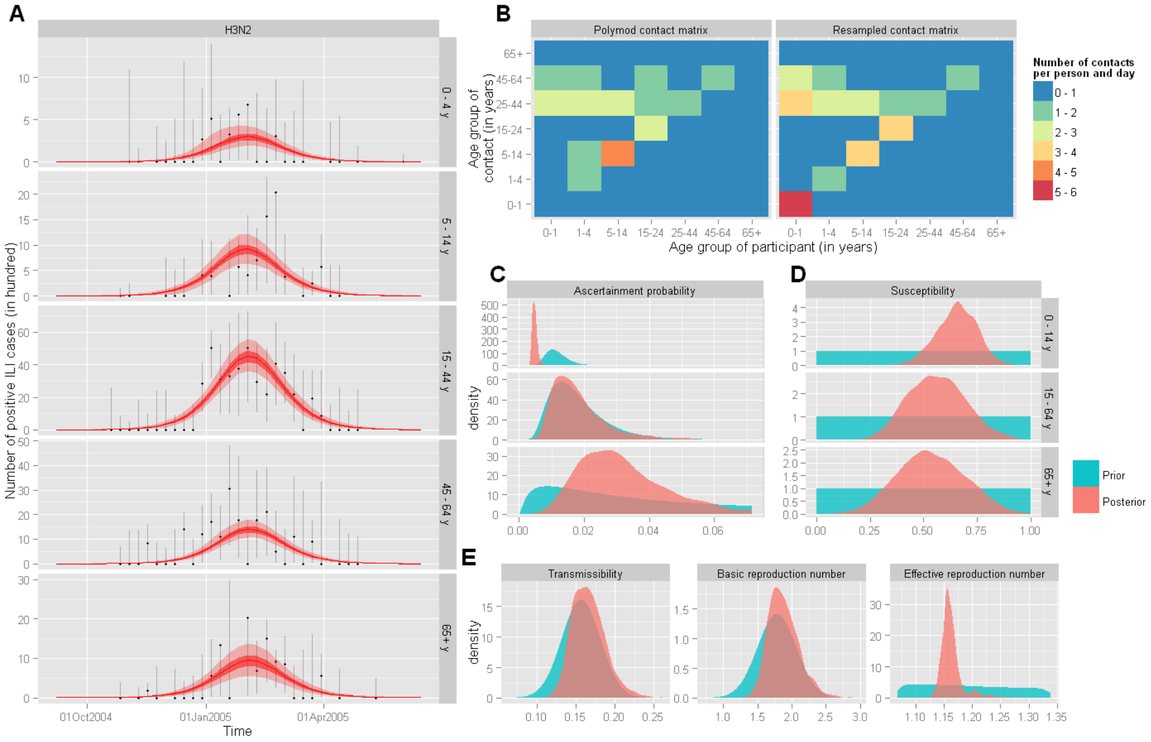

Figure S33: Inference results for H3N2 during the 2004-05 season. See the legend of the Figure S10 for more details.

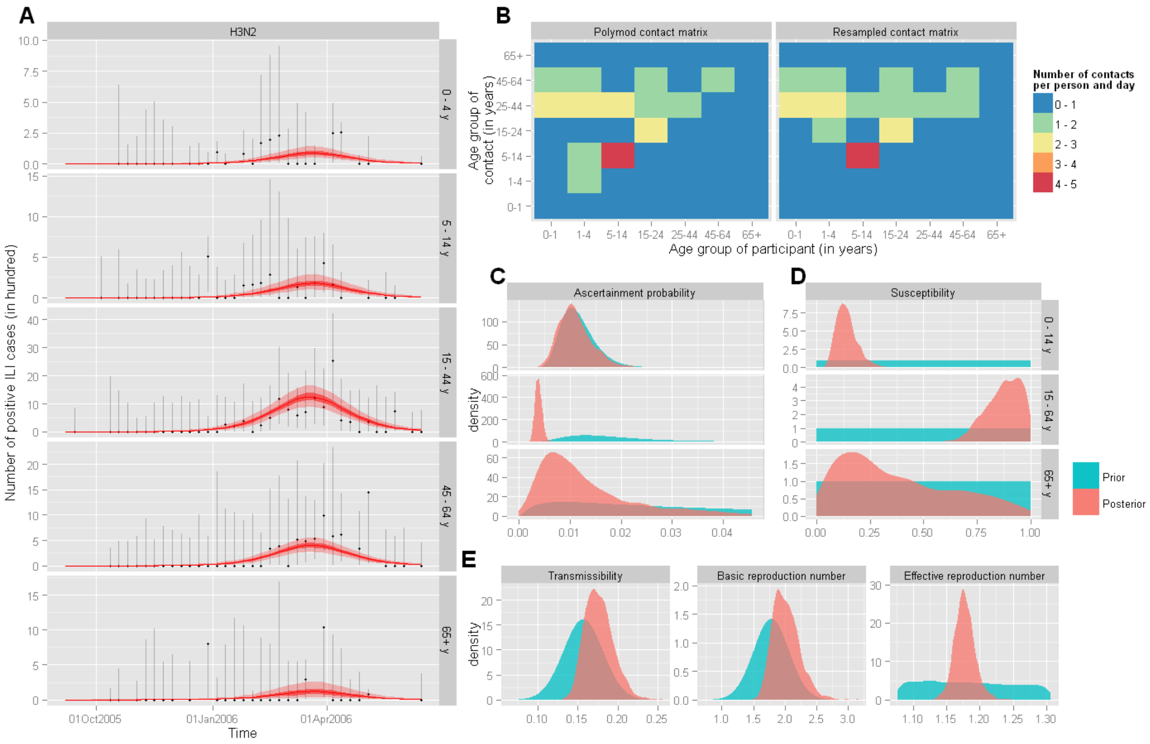

Figure S34: Inference results for H3N2 during the 2005-06 season. See the legend of the Figure S10 for more details.

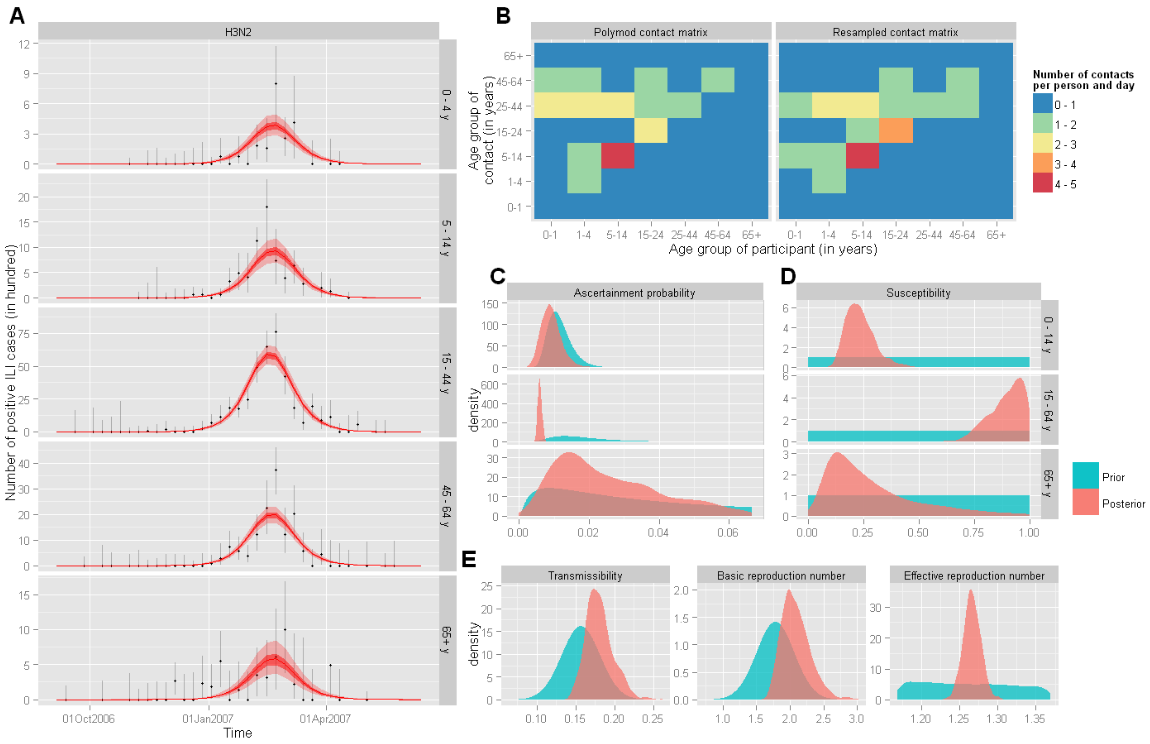

Figure S35: Inference results for H3N2 during the 2006-07 season. See the legend of the Figure S10 for more details.

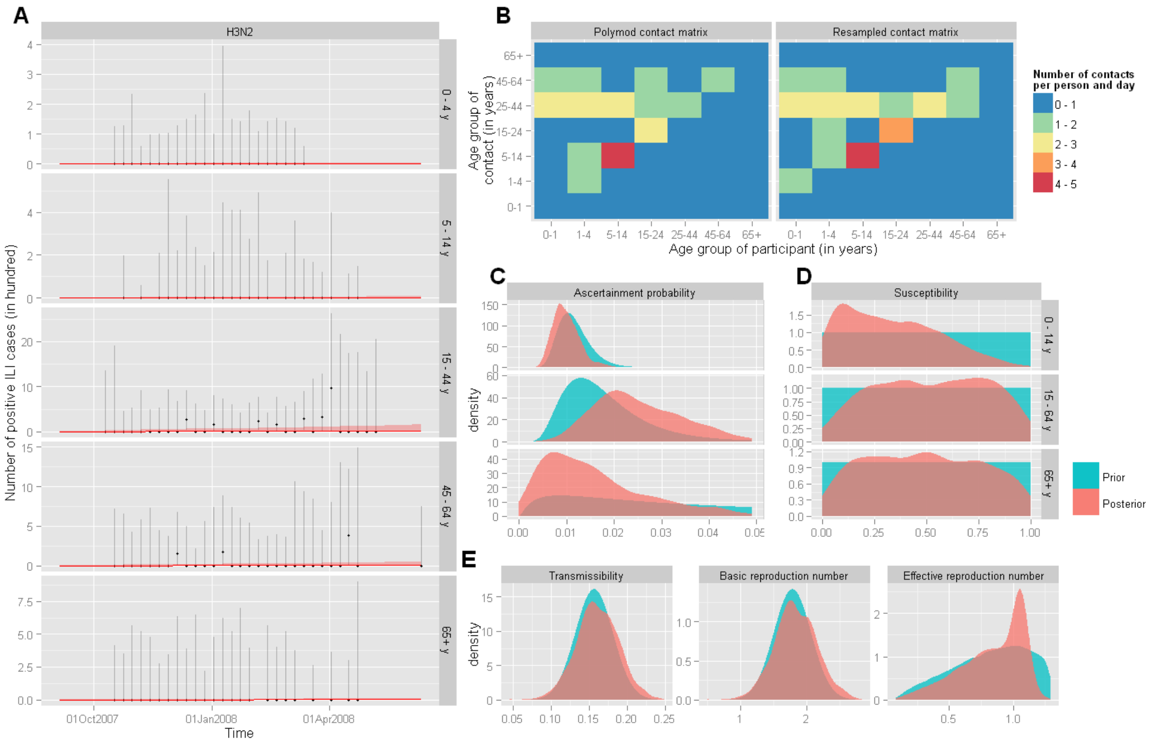

Figure S36: Inference results for H3N2 during the 2007-08 season. See the legend of the Figure S10 for more details.

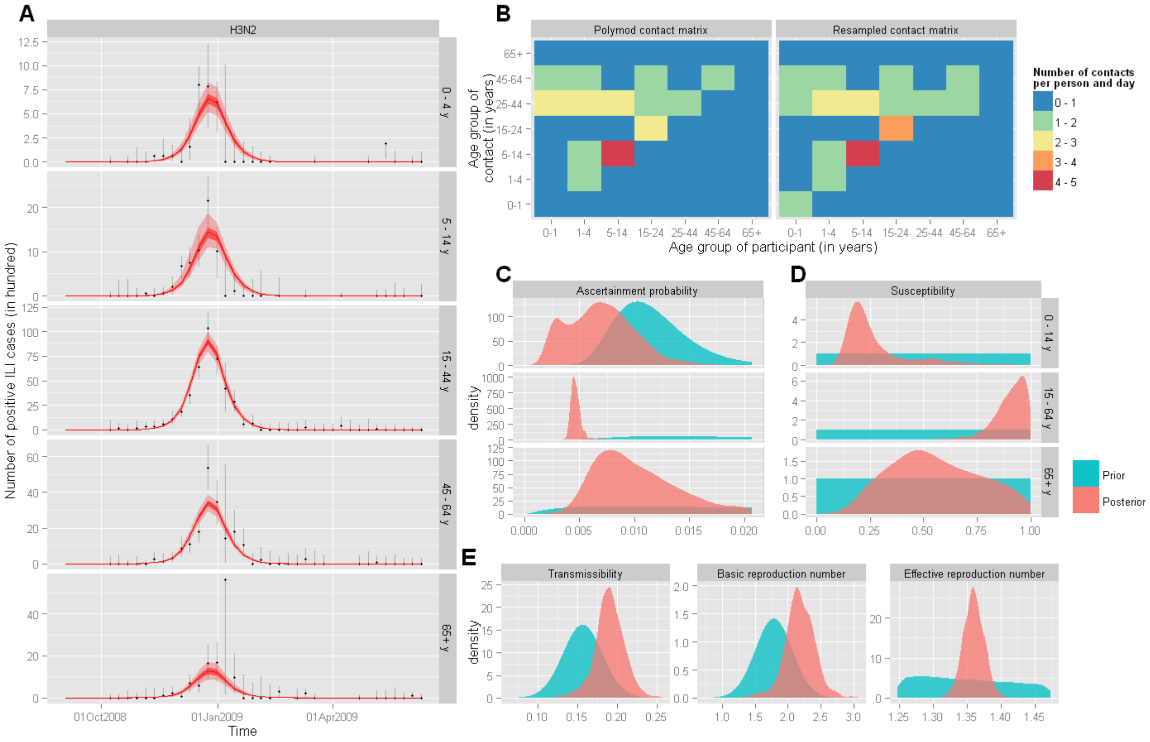

Figure S37: Inference results for H3N2 during the 2008-09 season. See the legend of the Figure S10 for more details.

3.1.5 Strain: B

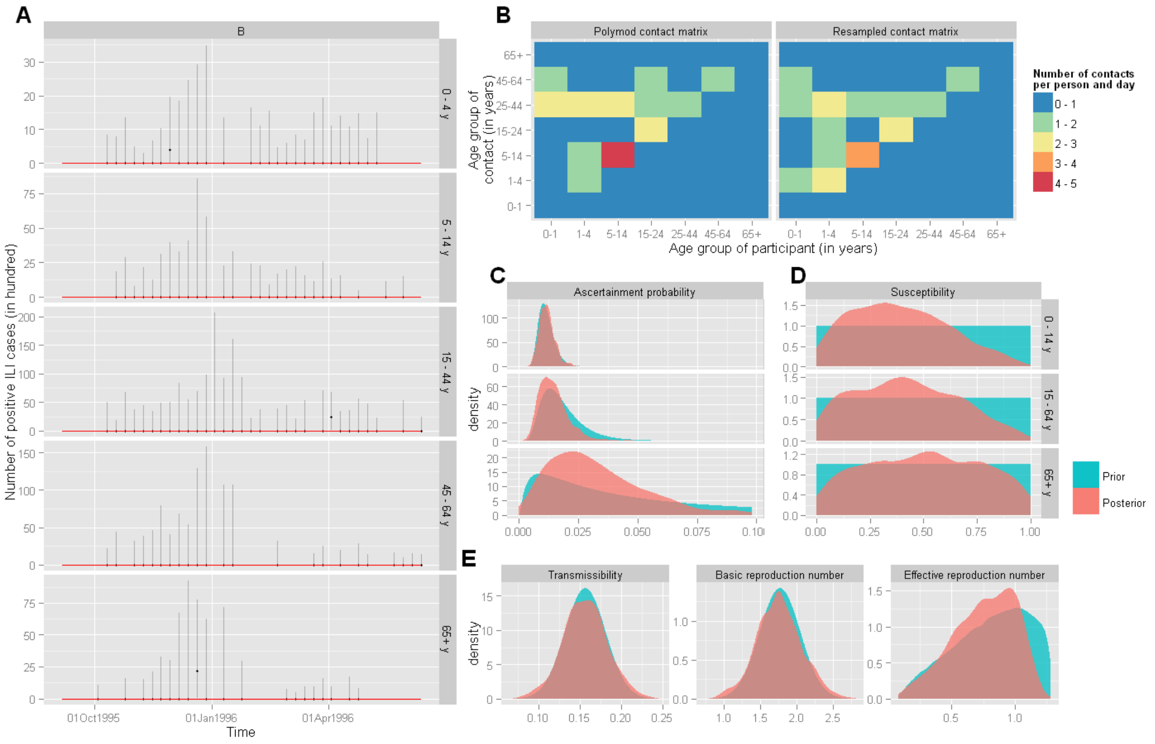

Figure S38: Inference results for B during the 1995-96 season. See the legend of the Figure S10 for more details.

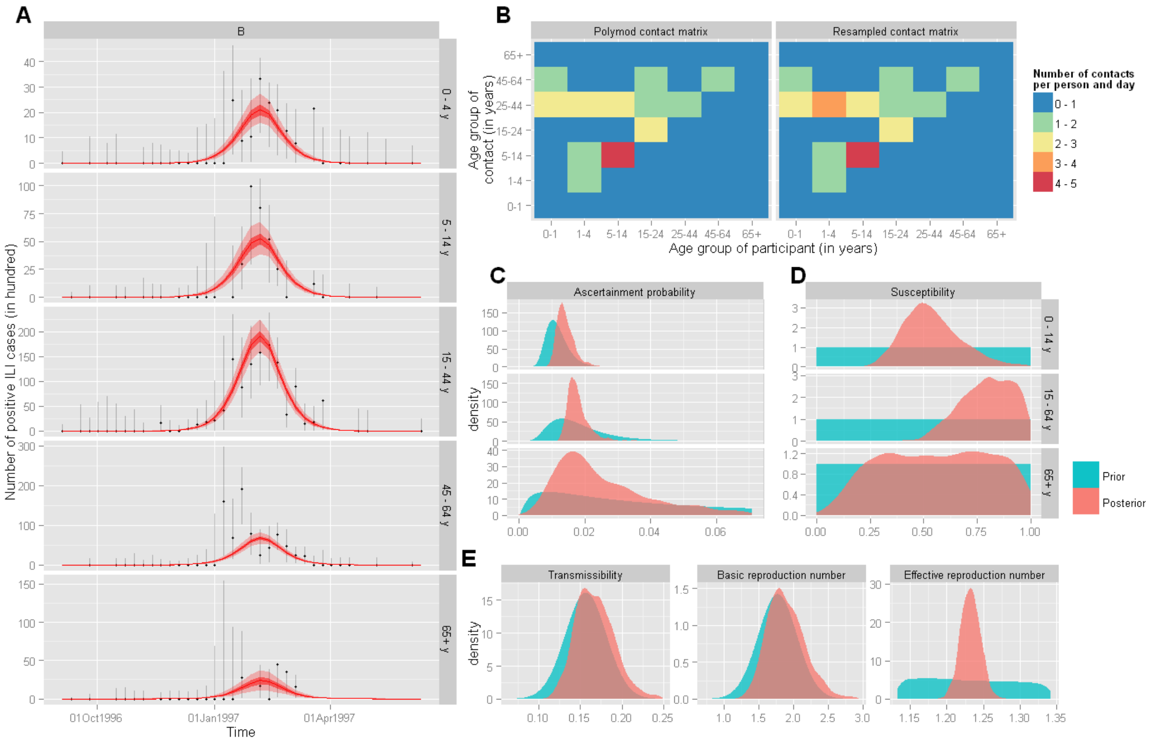

Figure S39: Inference results for B during the 1996-97 season. See the legend of the Figure S10 for more details.

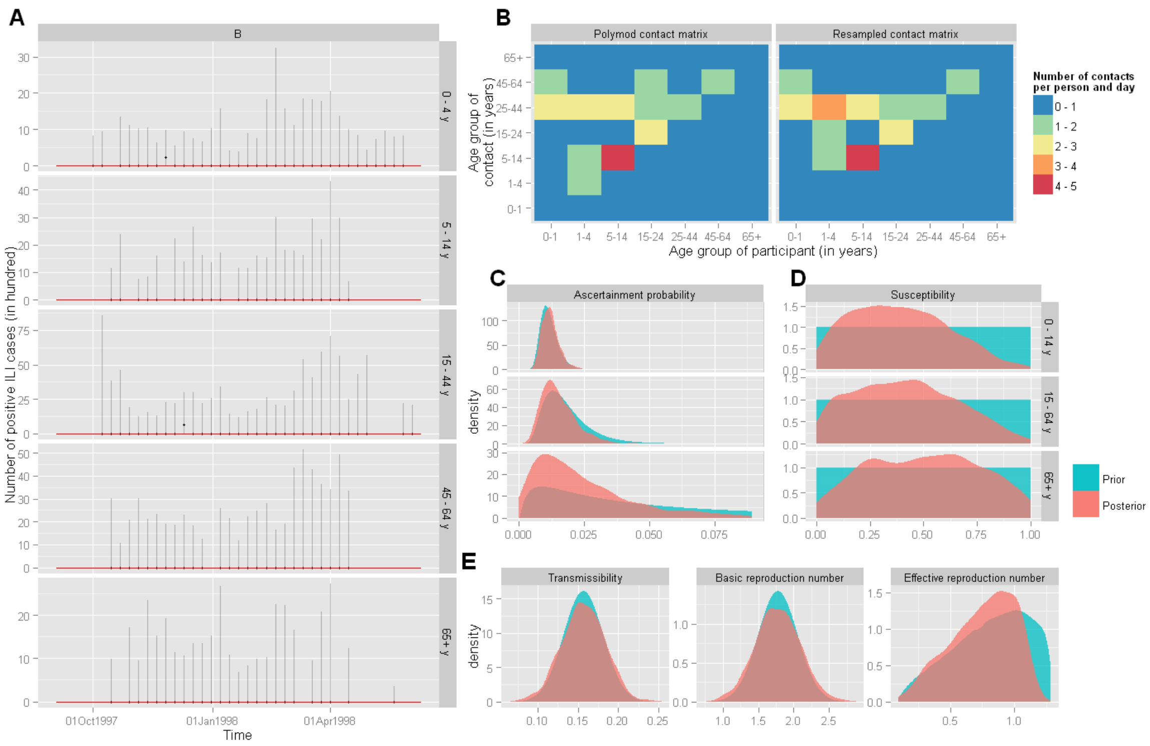

Figure S40: Inference results for B during the 1997-98 season. See the legend of the Figure S10 for more details.

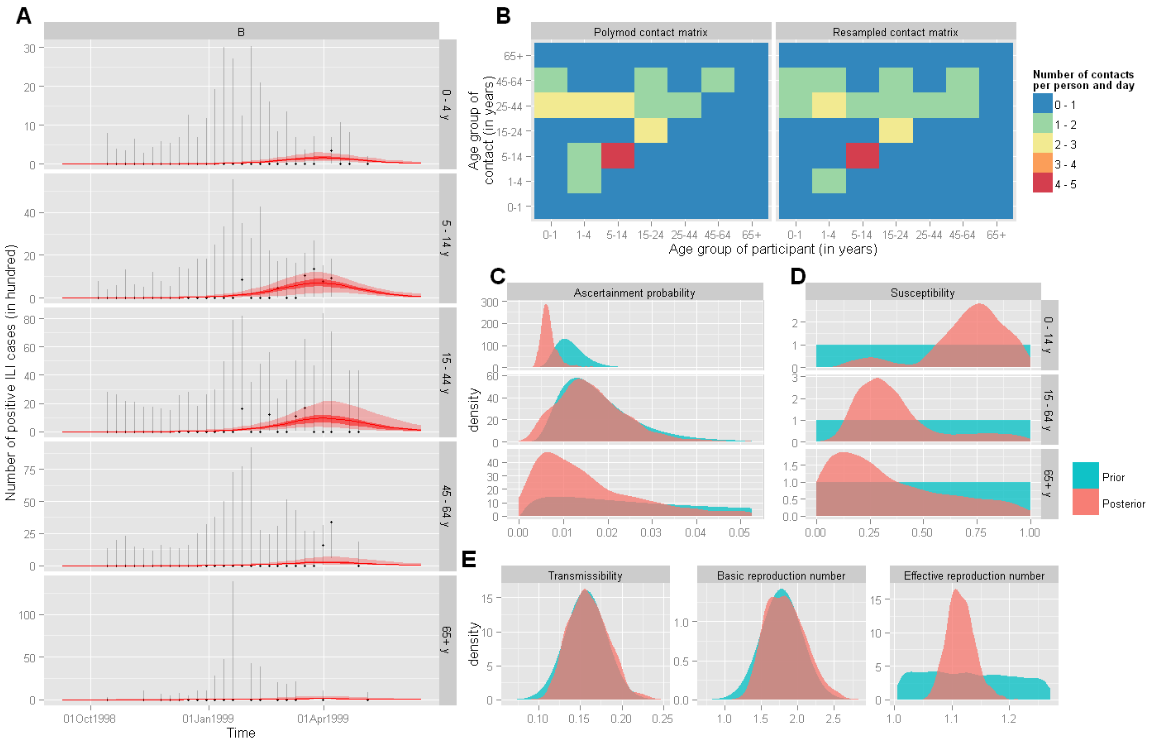

Figure S41: Inference results for B during the 1998-99 season. See the legend of the Figure S10 for more details.

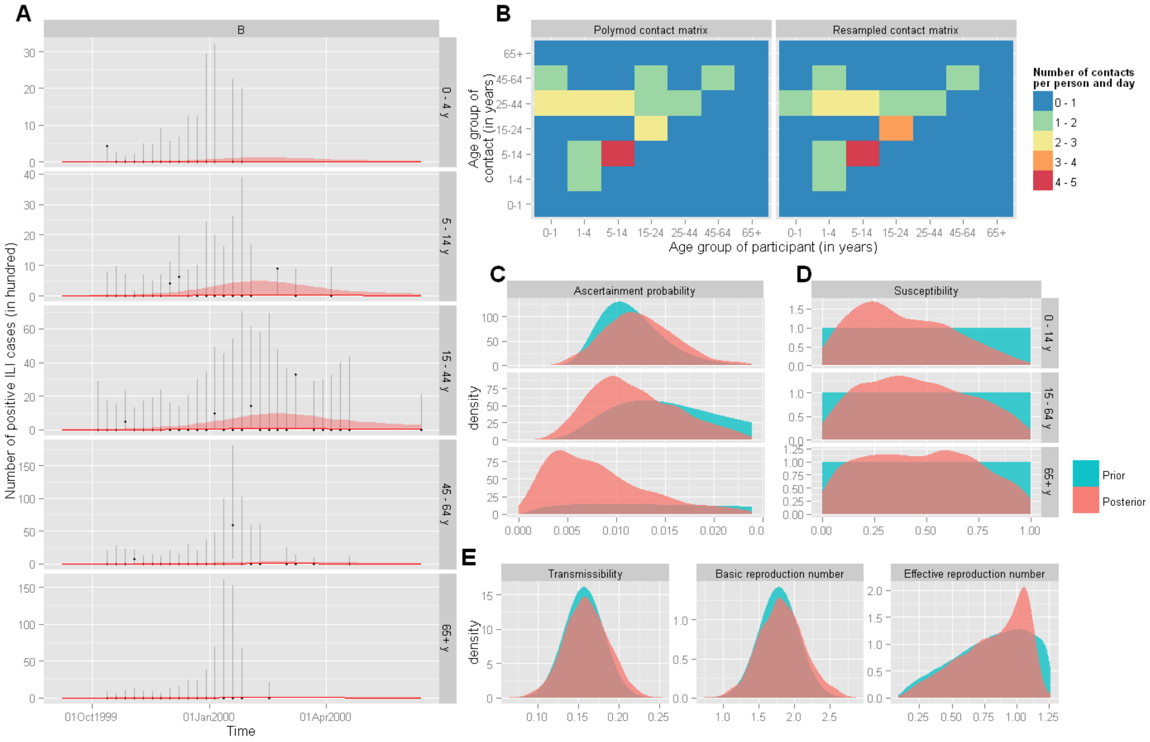

Figure S42: Inference results for B during the 1999-00 season. See the legend of the Figure S10 for more details.

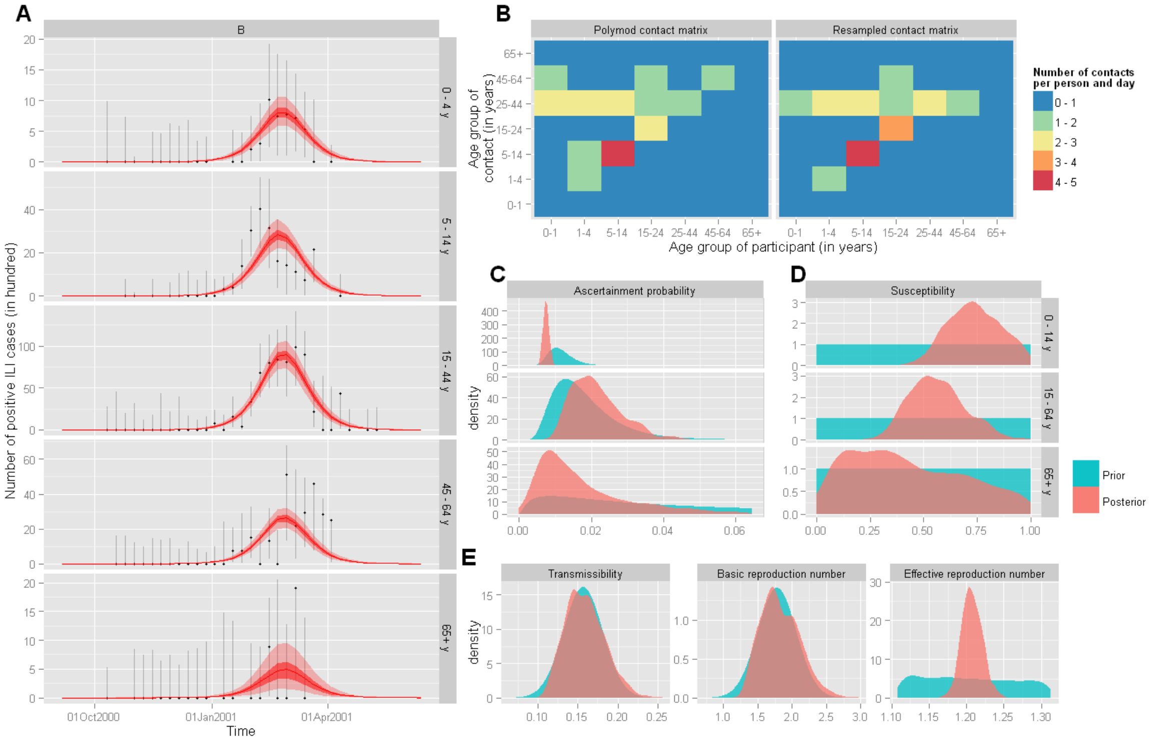

Figure S43: Inference results for B during the 2000-01 season. See the legend of the Figure S10 for more details.

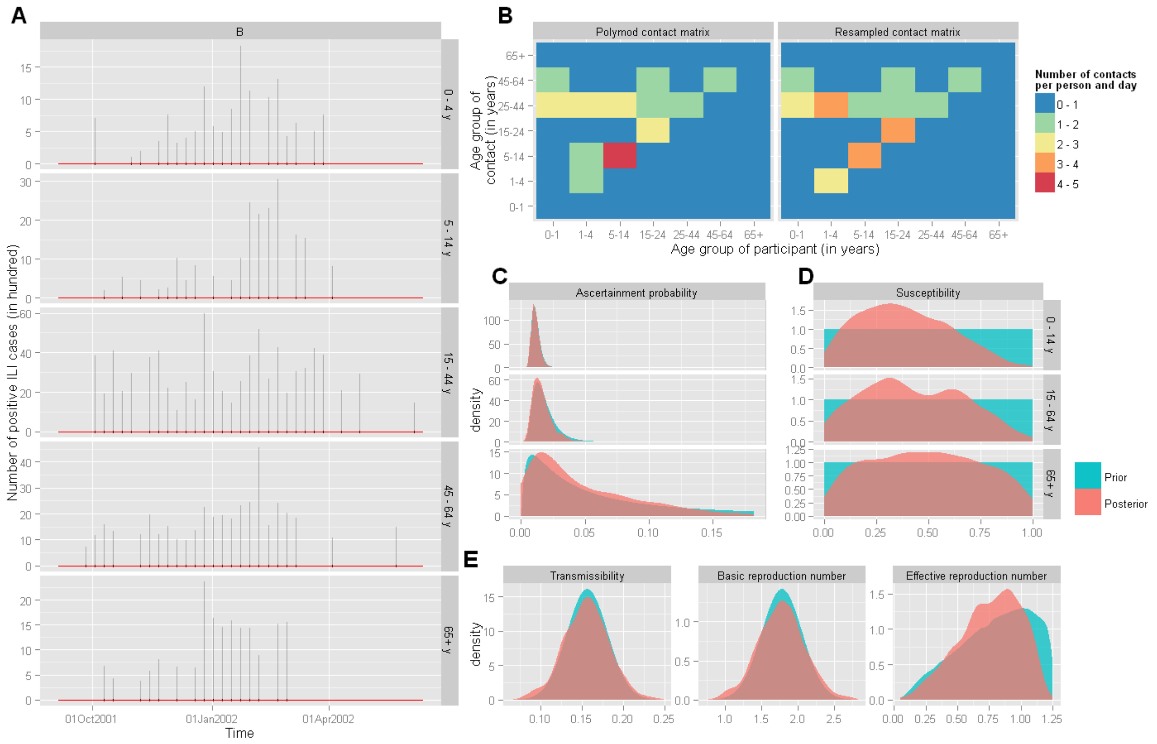

Figure S44: Inference results for B during the 2001-02 season. See the legend of the Figure S10 for more details.

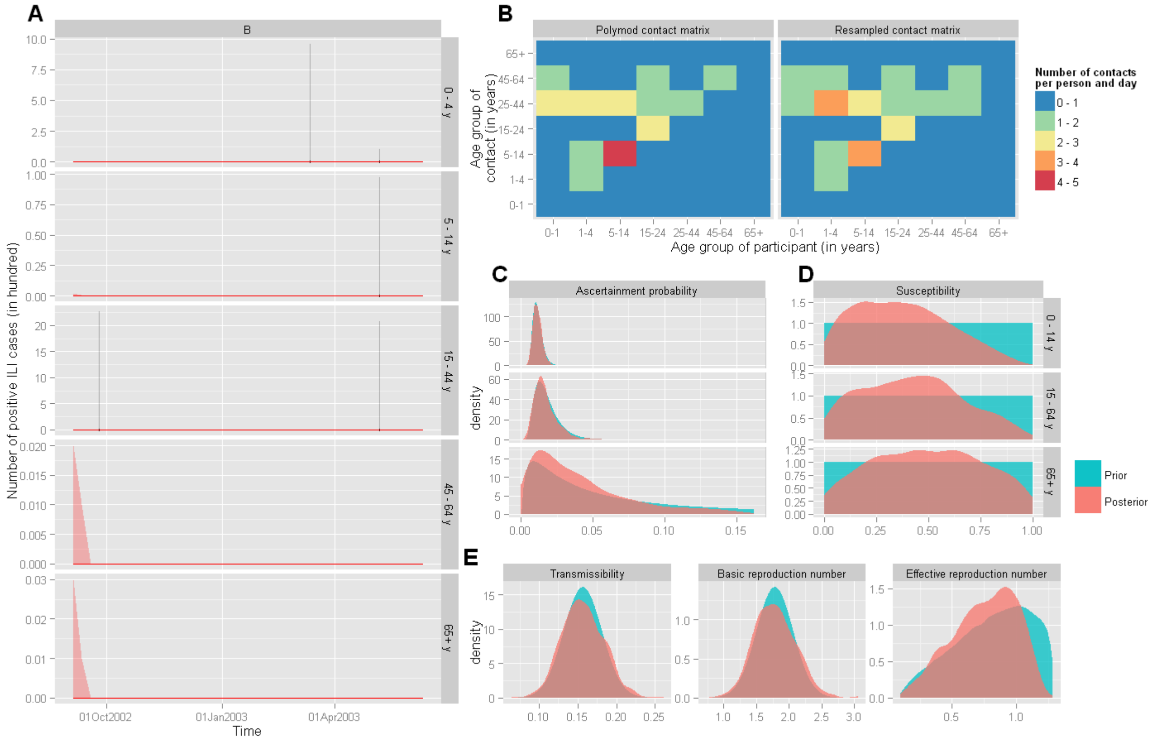

Figure S45: Inference results for B during the 2002-03 season. See the legend of the Figure S10 for more details.

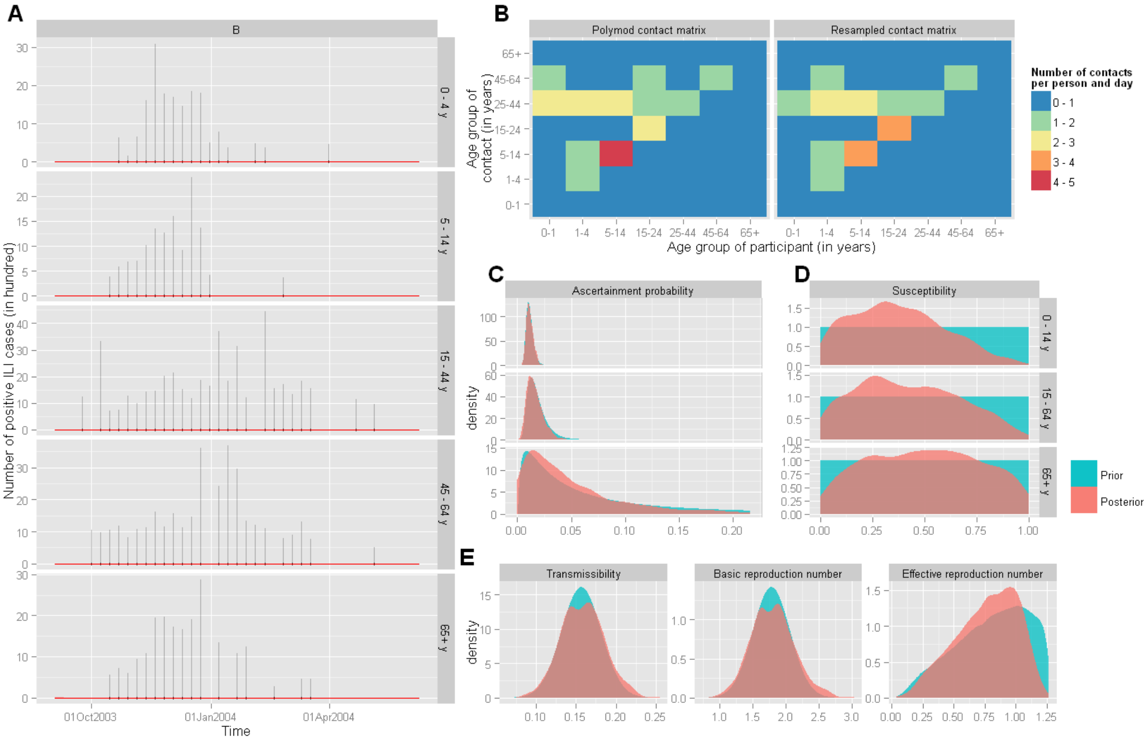

Figure S46: Inference results for B during the 2003-04 season. See the legend of the Figure S10 for more details.

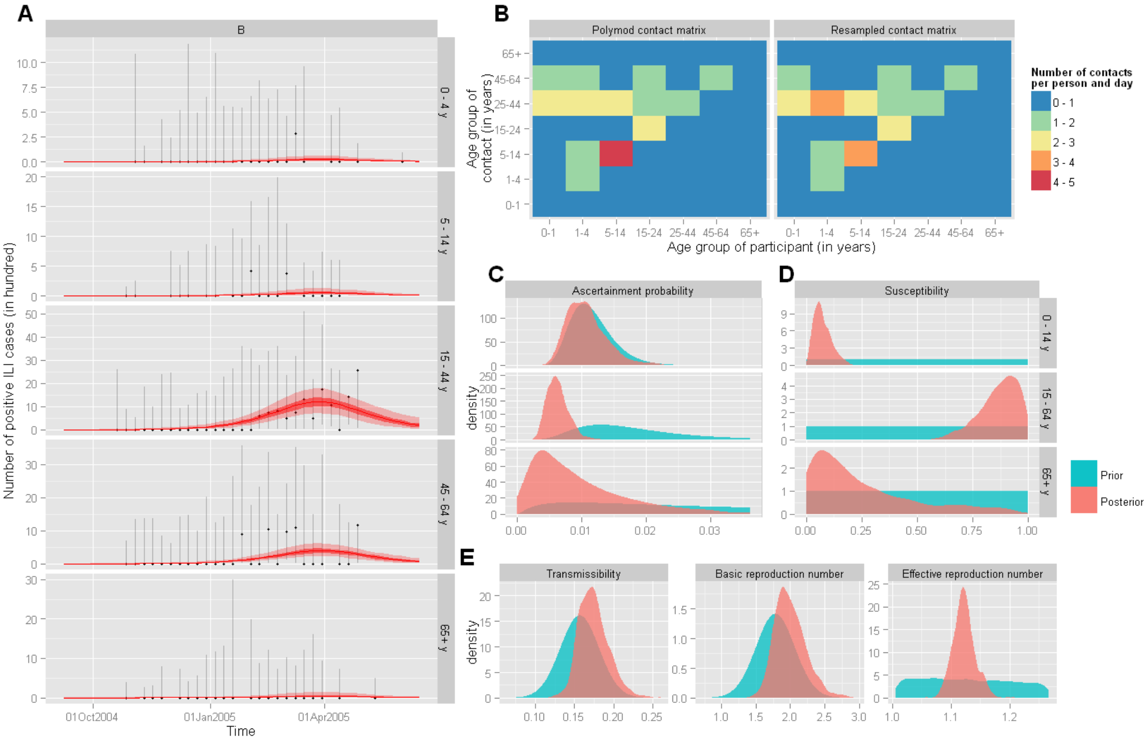

Figure S47: Inference results for B during the 2004-05 season. See the legend of the Figure S10 for more details.

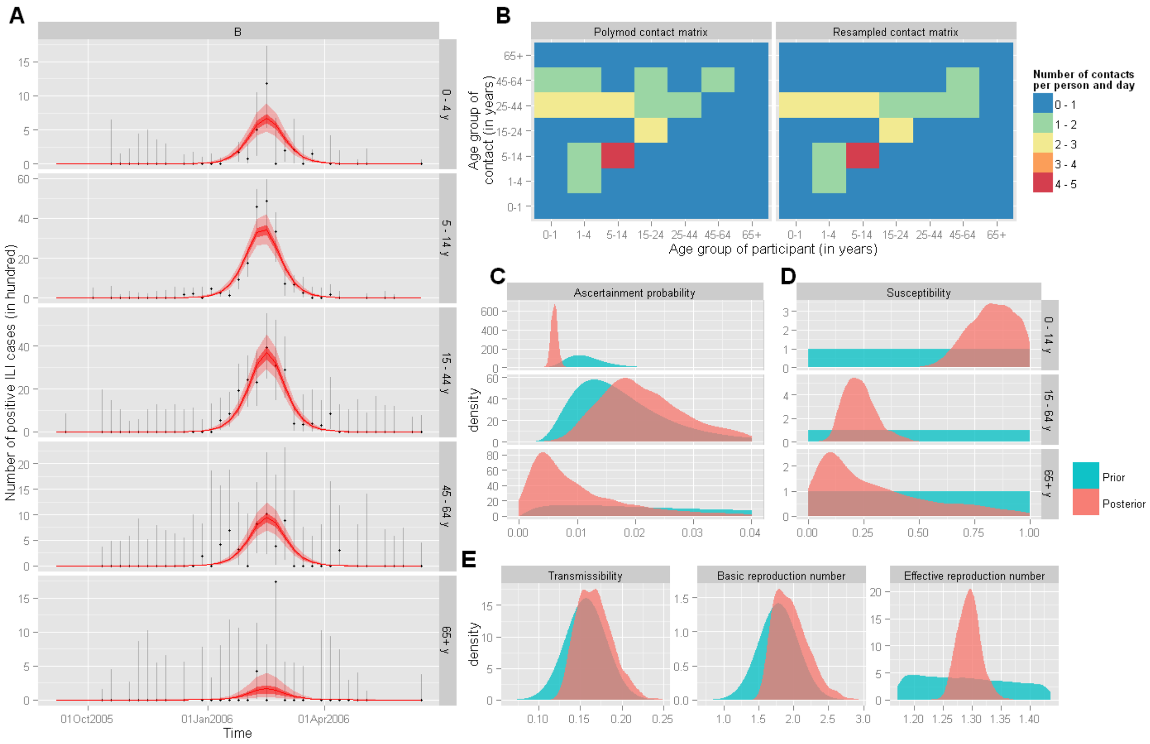

Figure S48: Inference results for B during the 2005-06 season. See the legend of the Figure S10 for more details.

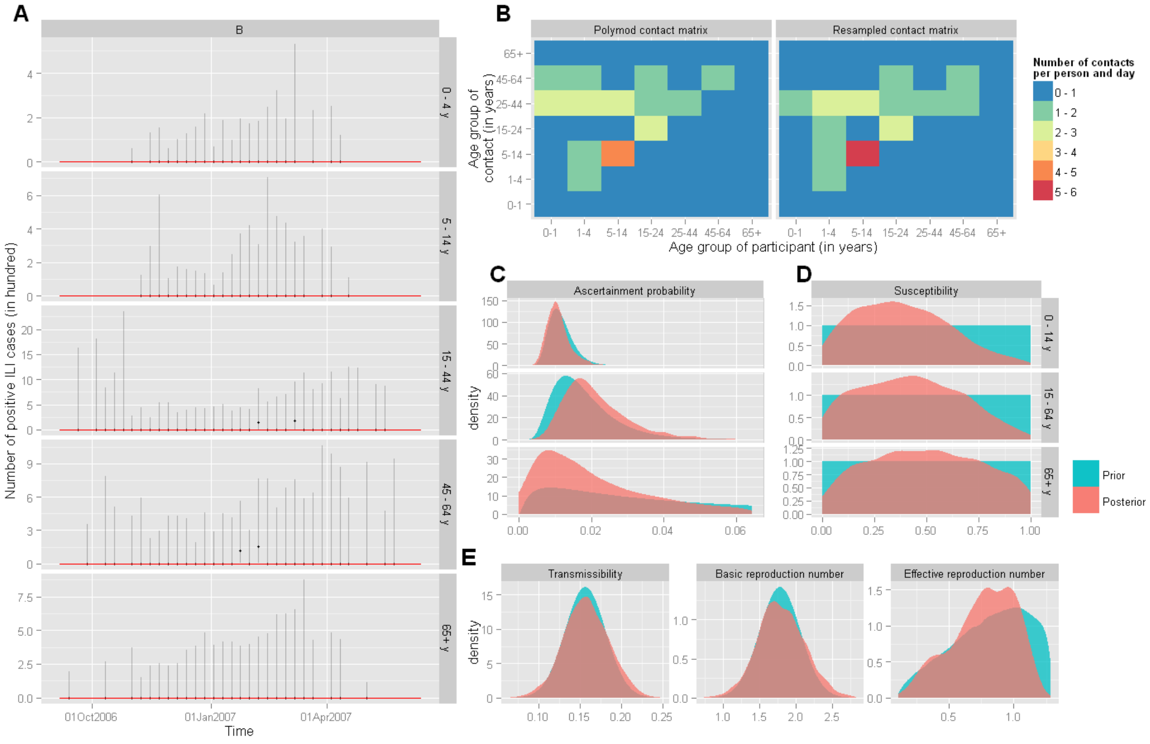

Figure S49: Inference results for B during the 2006-07 season. See the legend of the Figure S10 for more details.

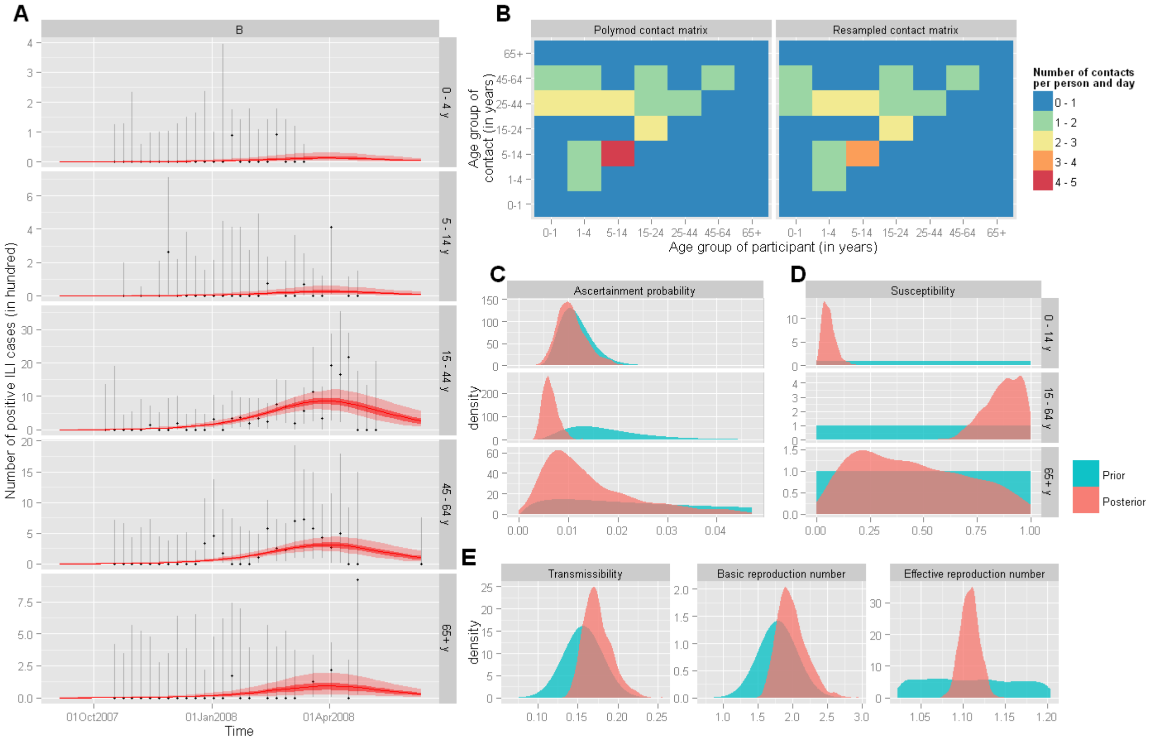

Figure S50: Inference results for B during the 2007-08 season. See the legend of the Figure S10 for more details.

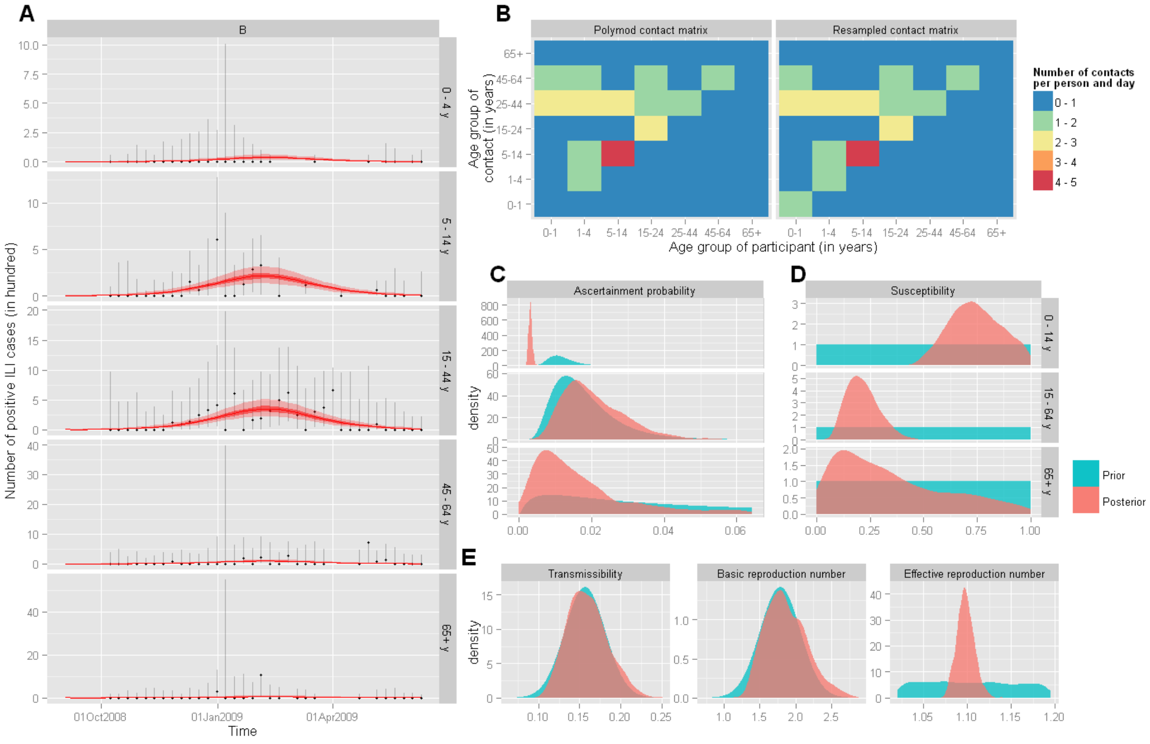

Figure S51: Inference results for B during the 2008-09 season. See the legend of the Figure S10 for more details.

3.2 Detailed time series by subtype and age group

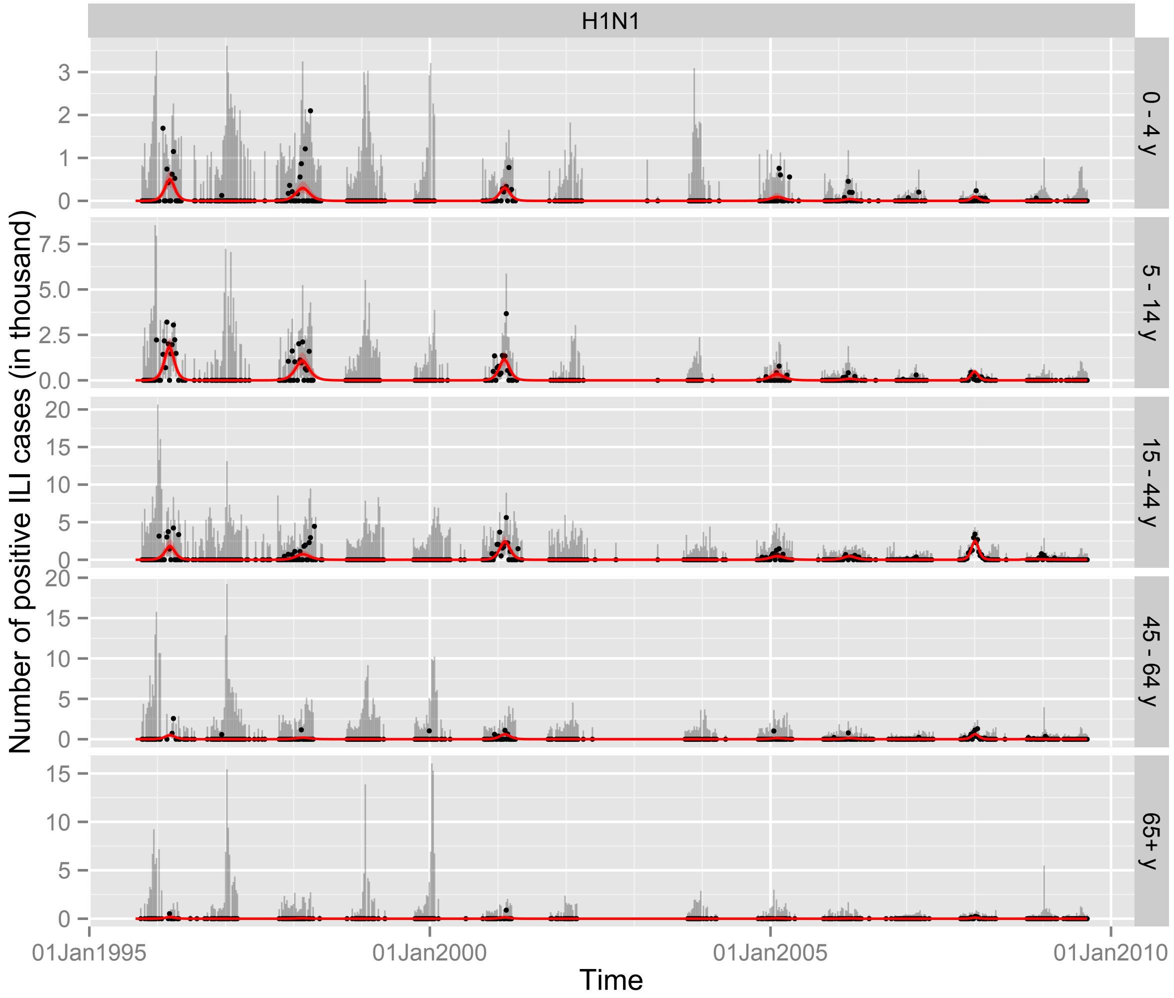

Figure S52: Inference results for H1N1 for the whole 14 years.

3.3 Posteriors of the contact matrices

The distribution of the posterior matrices during the epidemic year are very close from the POLYMOD prior distribution. This indicates the consistency of POLYMOD data with the observed epidemics but also illustrates the uncertainty present in the data. Little is learnt in terms of contact structure beyond the information contained in the POLYMOD survey. To illustrate the similarity between the posterior distributions of the inferred contact matrices and the POLYMOD prior, we have plotted the posterior of the dominant eigenvalues of these matrices for the seasons where a particular strain was circulating (Figure S55). The posterior of the contact values overlap almost entirely with the prior POLYMOD matrix.

3.4 Assortativity and reproduction numbers

Figure S56 shows the values of the specific reproductive numbers (R_{CC} and R_{AA}) and the assortativity (d_R) computed from the posterior distributions of the model for the 23 strain-specific seasonal outbreaks observed during the 14 seasons of the study. Figure S56 shows the values of the specific effective reproductive numbers ($R_{e_{CC}}$ and $R_{e_{AA}}$).

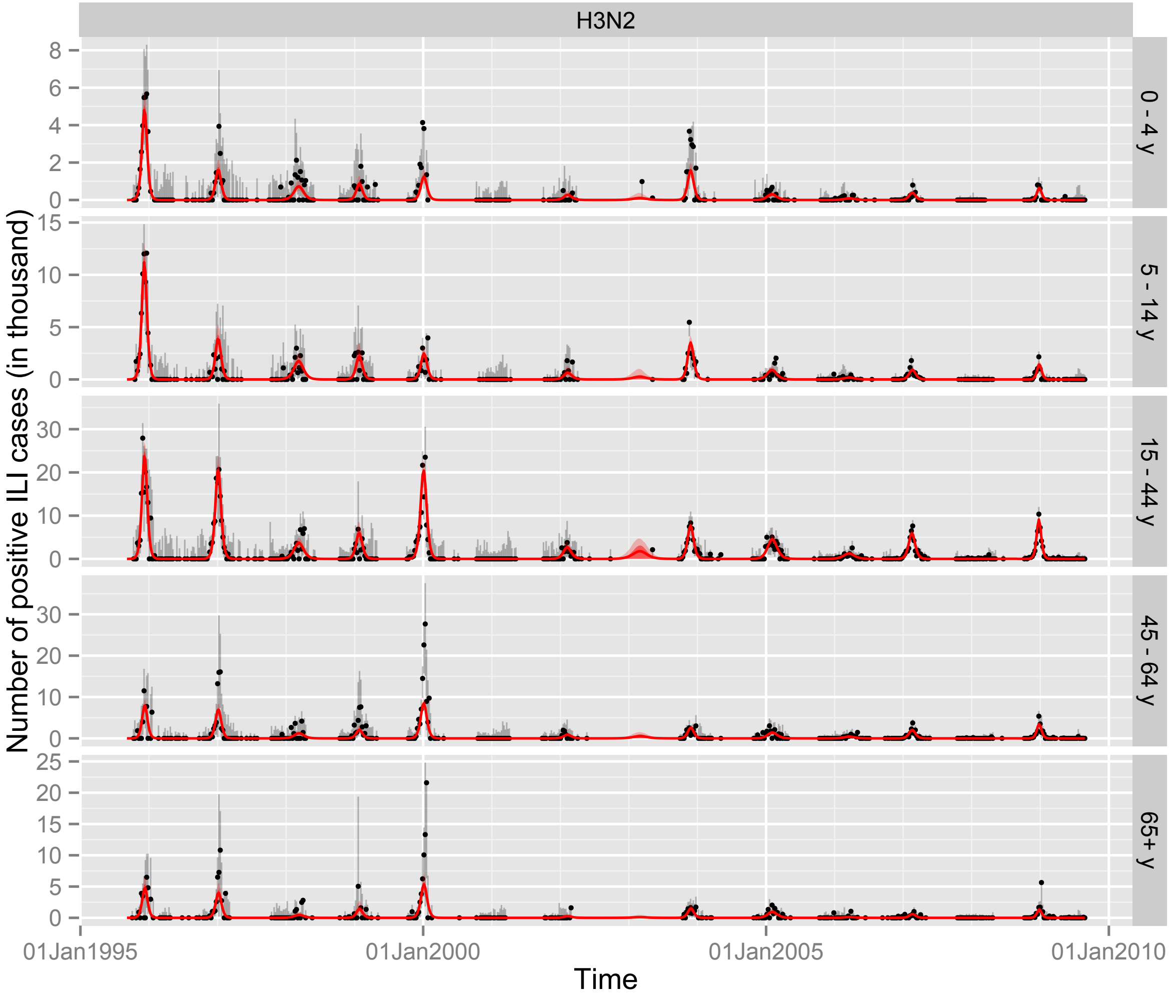

Figure S53: Inference results for H3N2 for the whole 14 years.

3.5 Pairwise significance of differences between strategies

Due to the structure of correlations, the posterior distributions of some of the outcomes measured to compare the different vaccination strategies might present overlapping credibility intervals. This is particularly true for the deaths due to the important uncertainties surrounding some of the CFRs. In order to assess the significance of the differences between strategies when analysed jointly (rather than comparing marginals), we computed in Tables S58 and S59 the p-values of the differences in term of cases and deaths averted per dose between each pairs of strategies.

3.6 Sensitivity analysis

To assess the robustness of our study, we tested the stability of the results to two variations from the original assumptions of our model.

3.6.1 Prior on transmissibility

We tested the sensitivity of the results to the choice of variance for the transmissibility prior (chosen to be 1.5 times the variance of the posterior of transmissibility for the year for which we had serology). For this we have chose to scale our prior by 1.2 and 1.8 instead of the 1.5 value. It can be seen in Figure S60 that the results are not affected by the change of variance of the transmissibility prior.

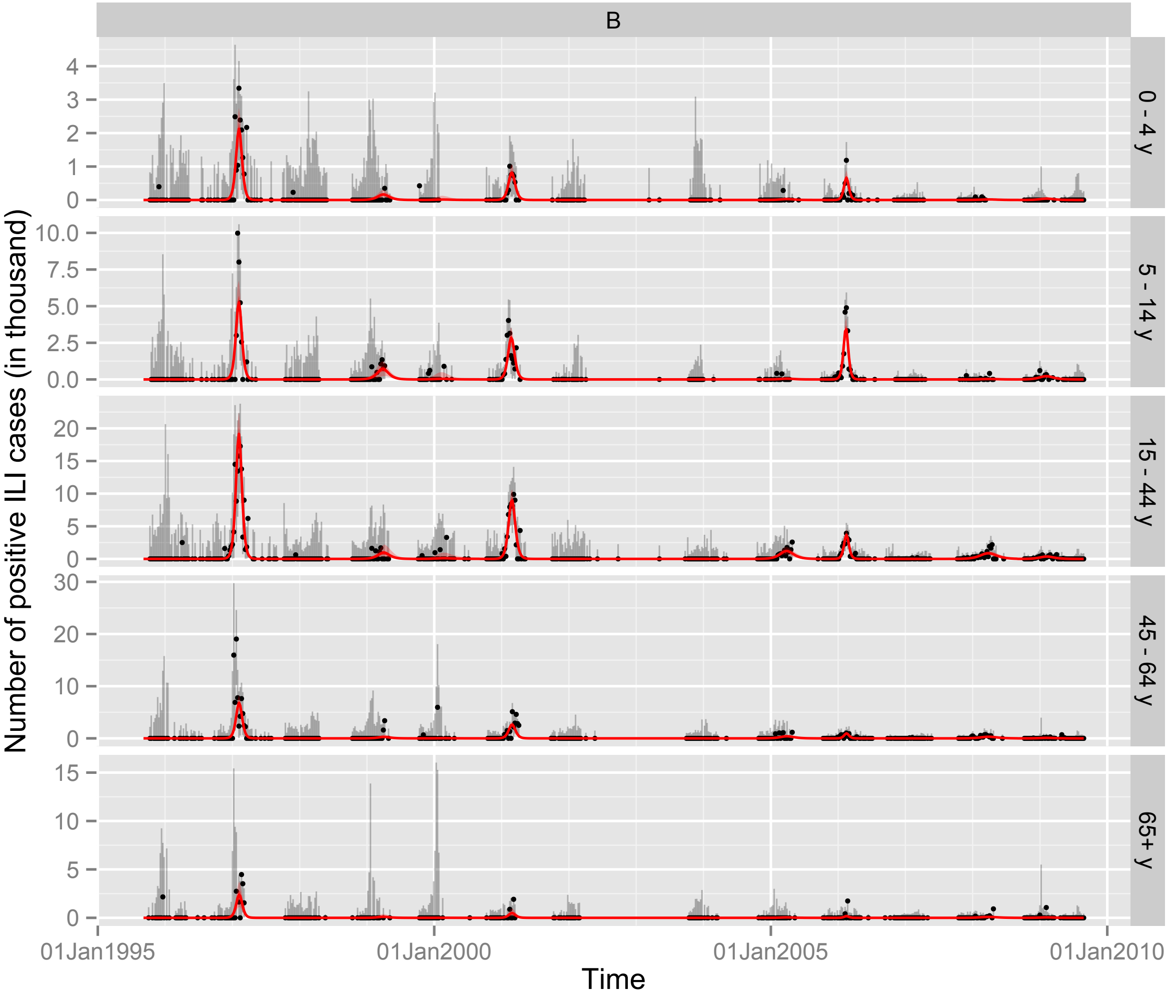

Figure S54: Inference results for B for the whole 14 years.

3.6.2 Improved vaccine efficacy in elderly

As a base case for the study, we assume based on Fleming at al. [28] that the vaccine efficacy is lower for the 65+ age group. Here we measure the impact of a possible improvement in vaccine efficacy for older age groups. We thus have tested the robustness of our conclusions to the assumption than the vaccine efficacy in 65+ year old is the same than for other age group. Results are shown in Figure S61. As expected, strategies involving vaccination of the elderly becomes more efficient, nevertheless the conclusions than vaccination targeting children is the most efficient strategy (in term of reducing infections and deaths) remains true.

References

[1] Hardelid P, Pebody R, Andrews N (2012) Mortality caused by influenza and respiratory syncytial virus by age group in England and Wales 1999-2010. *Influenza and other respiratory viruses* pp 13–16.

[2] Mauskopf J, Klesse M, Lee S, Herrera-Taracena G (2012) The burden of influenza complications in different high-risk groups: a targeted literature review. *Journal of medical economics*.

[3] World Health Organization (2005) Influenza vaccines - WHO position paper.

[4] World Health Organisation (2012) Weekly epidemiological record Relevé épidémiologique hebdomadaire. *Weekly epidemiological record* pp 461–476.

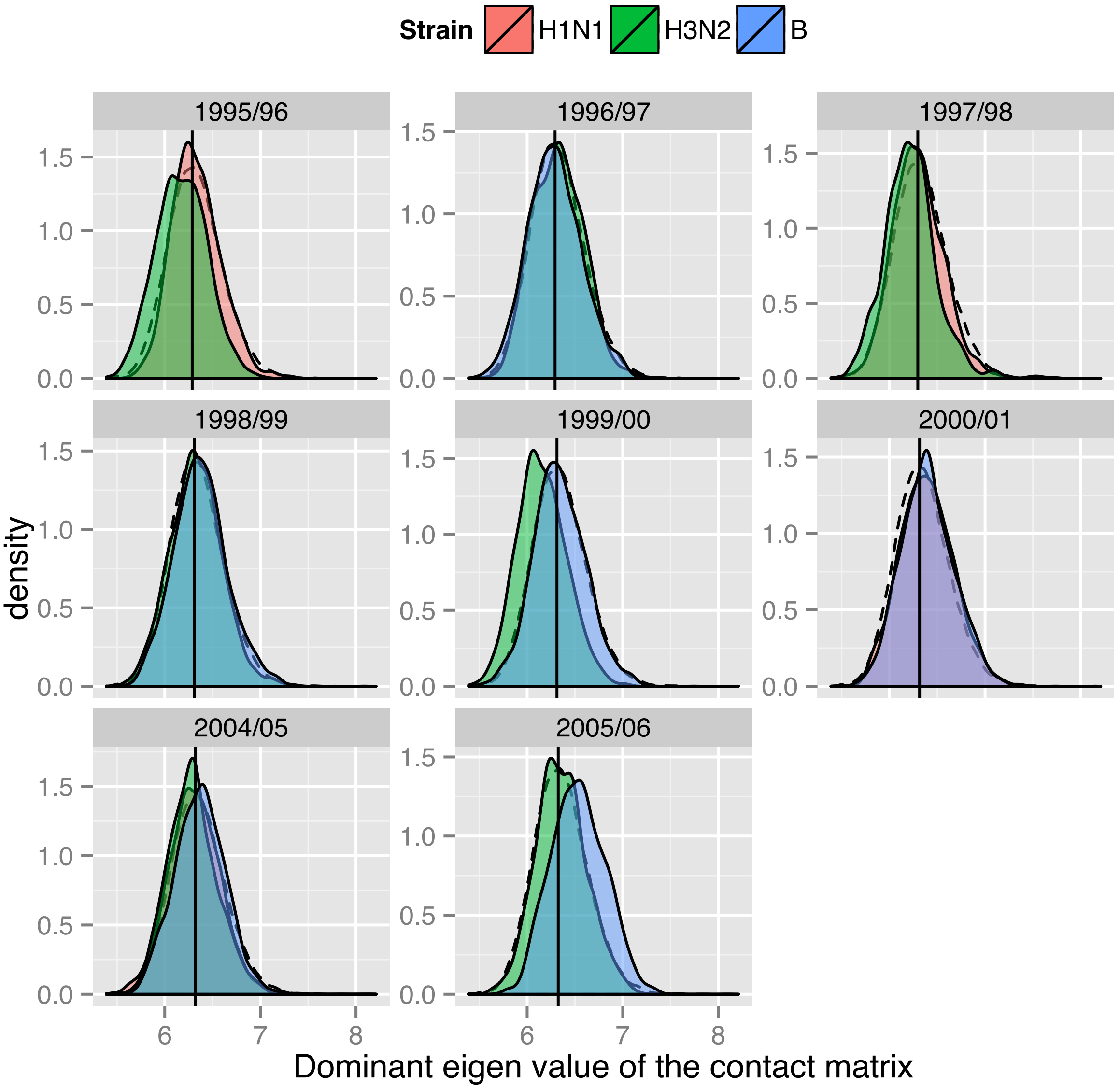

Figure S55: Values (posterior distributions) of the posterior of the dominant eigen value of the contact matrices. Coulours indicate the strain circulating (red: H1N1, green: H3N2 and blue: B) and dashed lines show the prior POLYMOD derived distribution.

[5] Viboud C, et al. (2004) Risk factors of influenza transmission in households. *The British journal of general practice : the journal of the Royal College of General Practitioners* 54:684–9.

[6] Cauchemez S, Valleron AJ, Boëlle PY, Flahault A, Ferguson NM (2008) Estimating the impact of school closure on influenza transmission from Sentinel data. *Nature* 452:750–4.

[7] Longini IM, Halloran ME (2005) Strategy for distribution of influenza vaccine to high-risk groups and children. *American journal of epidemiology* 161:303–6.

[8] Vynnycky E, Pitman R, Siddiqui R, Gay N, Edmunds WJ (2008) Estimating the impact of childhood influenza vaccination programmes in England and Wales. *Vaccine* 26:5321–30.

[9] Basta NE, Chao DL, Halloran ME, Matrajt L, Longini IM (2009) Strategies for pandemic and seasonal influenza vaccination of schoolchildren in the United States. *American journal of epidemiology* 170:679–86.

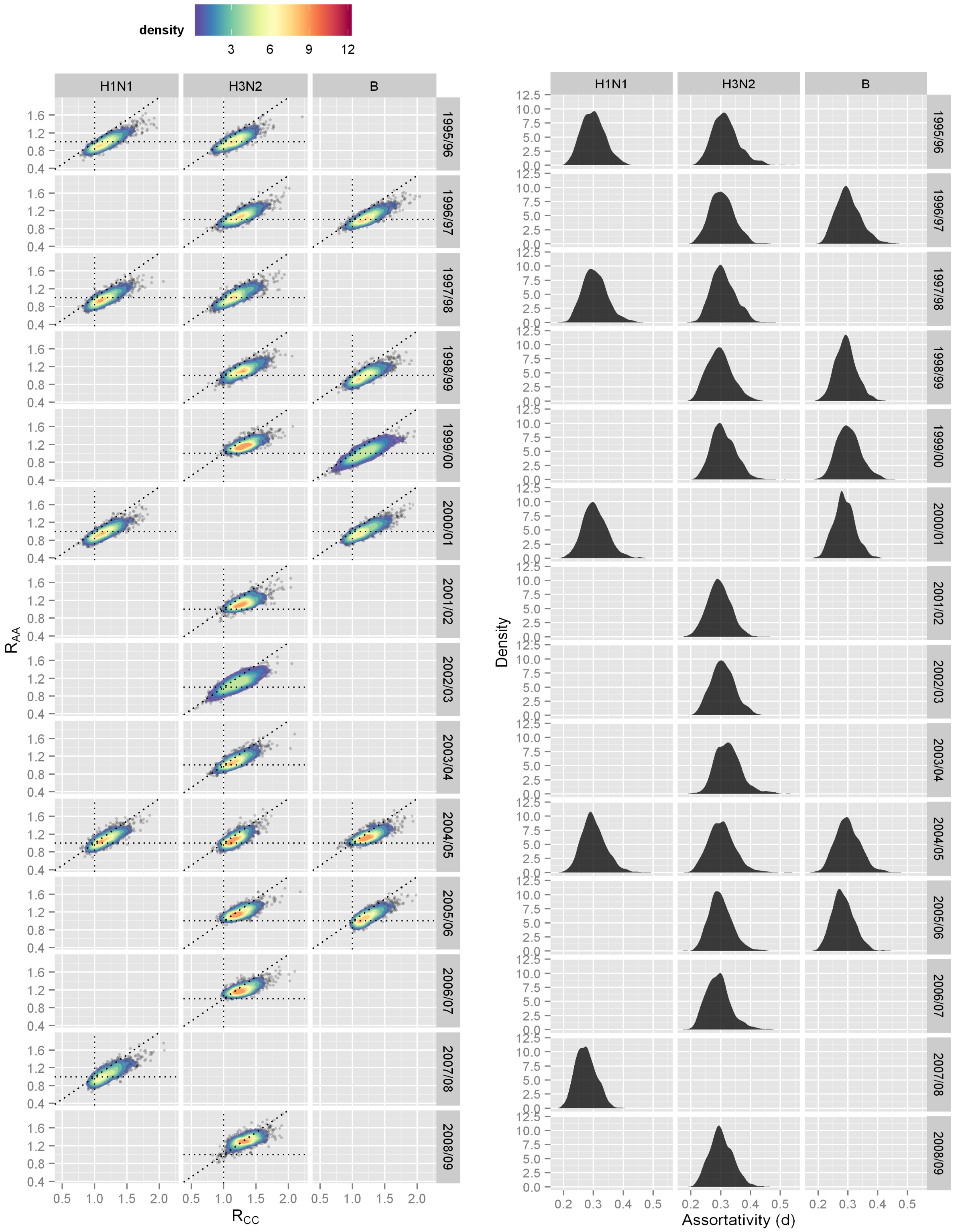

Figure S56: (left panel) Values (posterior distributions) of the specific reproductive numbers for children and adults R_{CC} and R_{AA} during the study period for all strains present at an epidemic level during the season. (right panel) Values (posterior distributions) of the assortativity during the study period for all strains present at an epidemic level during the season.

	Baseline name	Doses (in million/year)	Incidence (in million)	Mortality (in thousand)	Infections averted (per dose)	Deaths averted (per 1000 doses)
1	no vaccination	0.0	15.3 [8.4 - 30.4]	29.8 [7.1 - 123.8]	0 [0 - 0]	0 [0 - 0]
2	65+ at 29.3% (as pre-2000)	4.8	13.1 [6.6 - 27.6]	20.6 [4.9 - 89.6]	0.47 [0.16 - 1.31]	1.76 [0.35 - 8.21]
3	historical	7.1	13 [6.5 - 27.6]	18.9 [4.4 - 88.1]	0.32 [0.11 - 0.93]	1.34 [0.28 - 6.25]
4	65+ at 70% (as post-2000)	8.0	12.9 [6.5 - 27.1]	18 [4.3 - 76]	0.31 [0.1 - 0.82]	1.39 [0.28 - 6.64]
5	all HR at 75%	10.0	10.3 [4.7 - 22.4]	15 [3.2 - 69.6]	0.51 [0.22 - 0.89]	1.4 [0.33 - 5.93]

Table S7: Effectiveness of baseline vaccination strategies. Numbers inside brackets indicate 95% credibility intervals.

[10] Pitman RJ, White LJ, Sculpher M (2012) Estimating the clinical impact of introducing paediatric influenza vaccination in England and Wales. *Vaccine* 30:1208–24.

[11] Presanis AM, et al. (2009) The severity of pandemic H1N1 influenza in the United States, from April to July 2009: a Bayesian analysis. *PLoS medicine* 6:e1000207.

[12] Baguelin M, et al. (2011) Age-specific incidence of A/H1N1 2009 influenza infection in England from sequential antibody prevalence data using likelihood-based estimation. *PloS one* 6:e17074.

[13] Lee VJ, et al. (2011) Comparability of different methods for estimating influenza infection rates over a single epidemic wave. *American journal of epidemiology* 174:468–78.

[14] Hens N, et al. (2009) Estimating the impact of school closure on social mixing behaviour and the transmission of close contact infections in eight European countries. *BMC infectious diseases* 9:187.

[15] Mossong J, et al. (2008) Social contacts and mixing patterns relevant to the spread of infectious diseases. *PLoS medicine* 5:e74.

[16] Fleming DM, Miles J (2010) The representativeness of sentinel practice networks. *Journal of public health (Oxford, England)* 32:90–6.

[17] Joseph C, Goddard N, Gelb D (2005) Influenza vaccine uptake and distribution in England and Wales using data from the General Practice Research Database, 1989/90-2003/04. *Journal of public health (Oxford, England)* 27:371–7.

[18] Health Protection Agency (2012) Influenza vaccination uptake monitoring on behalf of the Department of Health.

[19] Johnson BF, et al. (2009) Fatal cases of influenza a in childhood. *PloS one* 4:e7671.

[20] Bernardo JM, Smith AFM (2000) *Bayesian Theory (Wiley Series in Probability and Statistics)* (Wiley), p 608.

[21] Roberts GO, Rosenthal JS (2001) Optimal scaling for various Metropolis-Hastings algorithms. *Statistical Science* 16:351–367.

[22] Lunn DJ, Thomas A, Best N, Spiegelhalter D (2000) WinBUGS - A Bayesian modelling framework: Concepts, structure, and extensibility. *Statistics and Computing* pp 325–337.

[23] Wallinga J, Teunis P, Kretzschmar M (2006) Using data on social contacts to estimate age-specific transmission parameters for respiratory-spread infectious agents. *American journal of epidemiology* 164:936–44.

[24] Kanaan MN, Farrington CP (2005) Matrix models for childhood infections: a Bayesian approach with applications to rubella and mumps. *Epidemiology and infection* 133:1009–21.

[25] Baguelin M, et al. (2010) Vaccination against pandemic influenza A/H1N1v in England: a real-time economic evaluation. *Vaccine* 28:2370–84.

[26] Miller E, et al. (2010) Incidence of 2009 pandemic influenza A H1N1 infection in England: a cross-sectional serological study. *Lancet* 375:1100–8.

[27] Jefferson T, et al. (2010) Vaccines for preventing influenza in healthy adults. *Cochrane database of systematic reviews (Online)* p CD001269.

[28] Fleming DM, et al. (2010) Estimating influenza vaccine effectiveness using routinely collected laboratory data. *Journal of epidemiology and community health* 64:1062–7.

[29] Department of Health (2008) Vaccination uptake among the 65 years and over and under 65 years at risk in England 2007-08.

[30] Ferguson NM, et al. (2005) Strategies for containing an emerging influenza pandemic in Southeast Asia. *Nature* 437:209–14.

[31] Carrat F, et al. (2008) Time lines of infection and disease in human influenza: a review of volunteer challenge studies. *American journal of epidemiology* 167:775–85.

[32] Russell CA, et al. (2008) The Global Circulation of Seasonal Influenza A (H3N2) Viruses. *Science* 320:340–346.

[33] Flasche S, et al. (2011) Different transmission patterns in the early stages of the influenza A(H1N1)v pandemic: A comparative analysis of 12 European countries. *Epidemics* 3:125–33.

[34] Anderson RM, May RM (1992) *Infectious Diseases of Humans: Dynamics and Control.*

[35] R Development Core Team (2012) *R: A Language and Environment for Statistical Computing* (R Foundation for Statistical Computing, Vienna, Austria) ISBN 3-900051-07-0.

[36] Tanner MA, Wong WH (1987) The Calculation of Posterior Distributions by Data Augmentation. *Journal of the American Statistical Association* 82:528–540.

[37] Loeb M, et al. (2010) Effect of influenza vaccination of children on infection rates in Hutterite communities: a randomized trial. *JAMA : the journal of the American Medical Association* 303:943–50.

[38] CDC (2012) Flu Vaccination Coverage, United States, 2011-12 Influenza Season.

[39] Brooks-Pollock E, Tilston N, Edmunds WJ, Eames KTD (2011) Using an online survey of healthcare-seeking behaviour to estimate the magnitude and severity of the 2009 H1N1v influenza epidemic in England. *BMC infectious diseases* 11:68.

[40] Dunning AJ (2006) A model for immunological correlates of protection. *Statistics in medicine* 25:1485–97.

[41] Coudeville L, et al. (2010) Relationship between haemagglutination-inhibiting antibody titres and clinical protection against influenza: development and application of a bayesian random-effects model. *BMC medical research methodology* 10:18.

[42] Black S, et al. (2011) Hemagglutination inhibition antibody titers as a correlate of protection for inactivated influenza vaccines in children. *The Pediatric infectious disease journal* 30:1081–5.

[43] Roberts GO, Rosenthal JS (2009) Examples of Adaptive MCMC. *Journal of Computational and Graphical Statistics* 18:349–367.

[44] Sherlock C, Fearnhead P, Roberts GO (2010) The Random Walk Metropolis: Linking Theory and Practice Through a Case Study. *Statistical Science* 25:172–190.

[45] Birrell PJ, et al. (2011) Bayesian modeling to unmask and predict influenza A/H1N1pdm dynamics in London. *Proceedings of the National Academy of Sciences of the United States of America* 108:18238–43.

[46] Dorigatti I, Cauchemez S, Pugliese A, Ferguson NM (2012) A new approach to characterising infectious disease transmission dynamics from sentinel surveillance: Application to the Italian 2009-2010 A/H1N1 influenza pandemic. *Epidemics* 4:9–21.

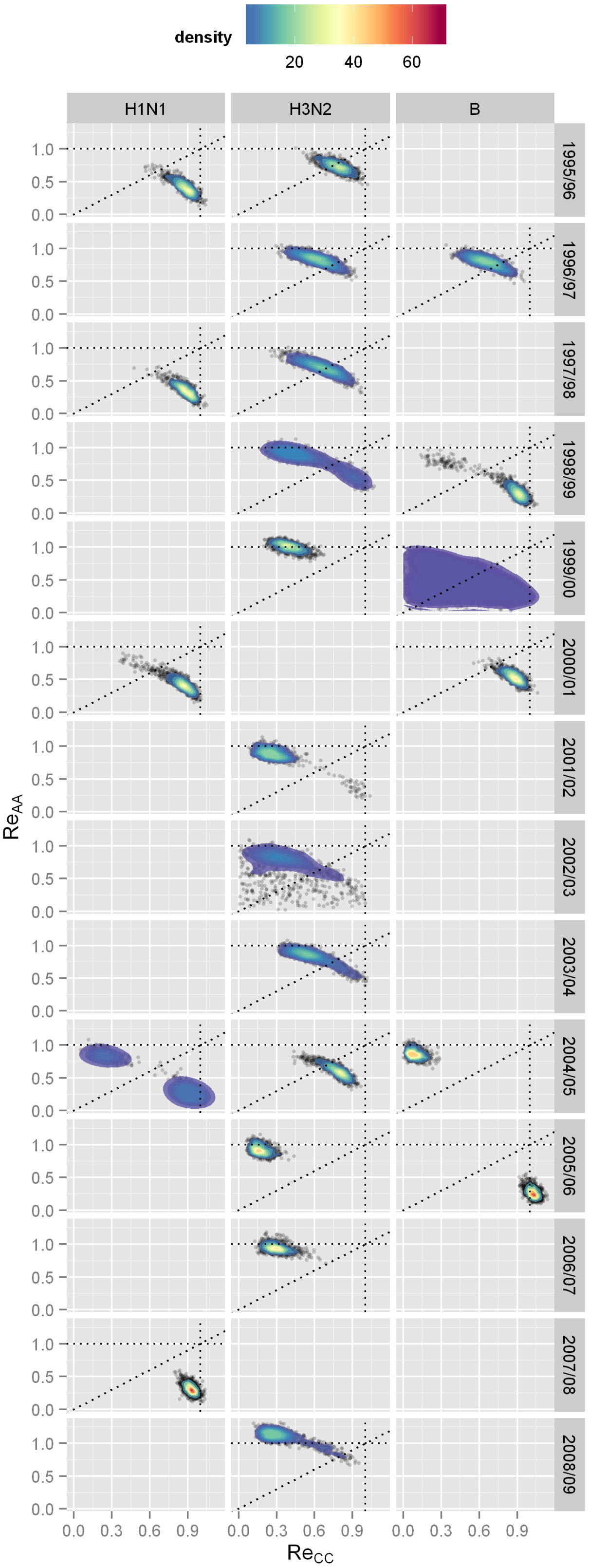

Figure S57: Values (posterior distributions) of the specific effective reproductive numbers for children and adults $R_{e_{CC}}$ and $R_{e_{AA}}$ during the study period for all strains present at an epidemic level during the season.

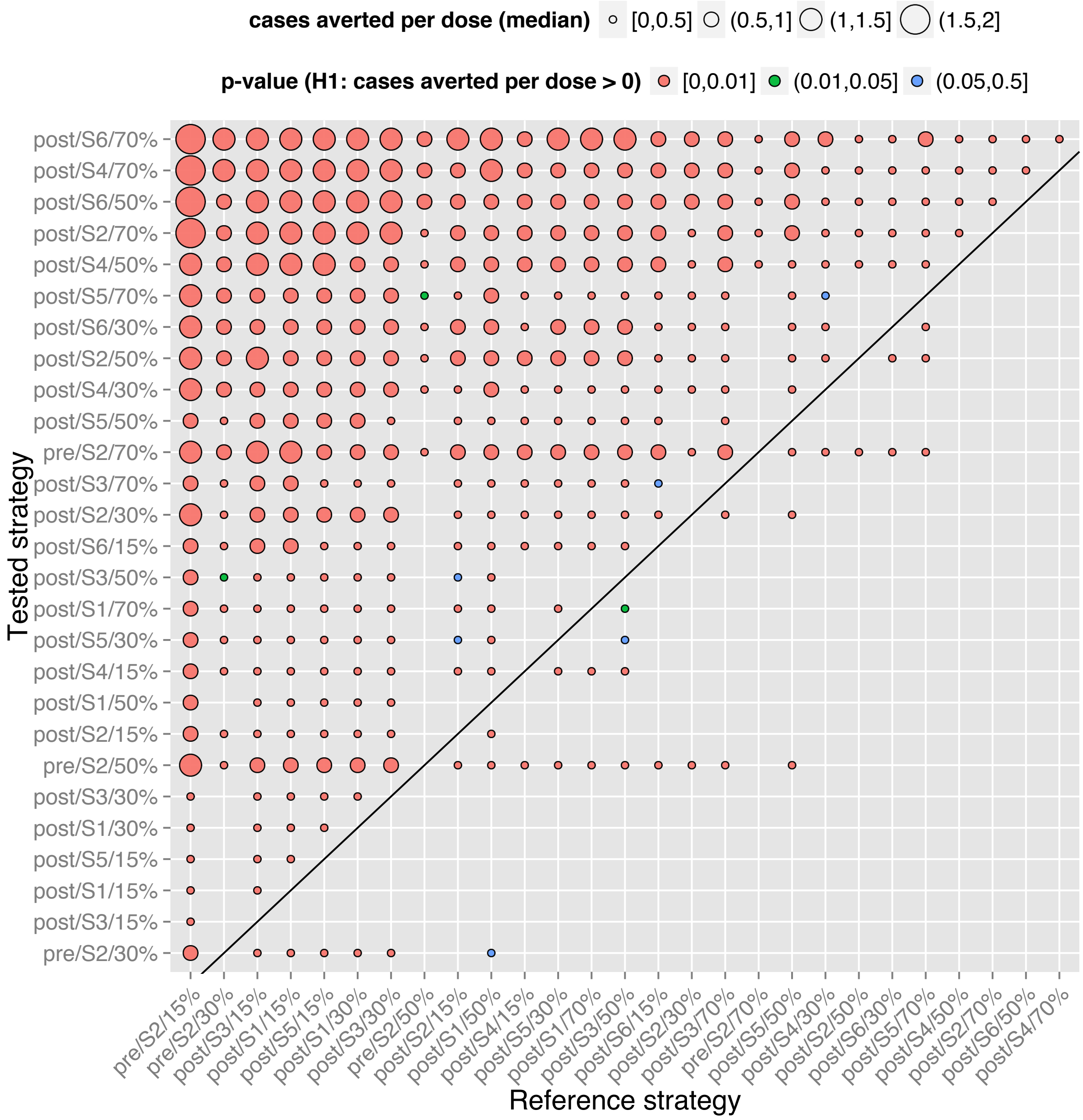

Figure S58: Computation of the pairwise significance of the differences observed between strategies in term of cases averted. Strategies are ordered by increasing number of doses given. A circle indicates that the tested strategy averts cases compared with the reference strategy. The size of circle gives the range of the difference, the colour indicates the significance of the difference between the two posterior distributions of the strategies. Red disks show high significance with p-values < 0.01, green disks show significance with p-values < 0.05, and blue disks show non significant differences with p-values between 0.05 and 0.5. The black diagonal line represents the bisector, tested strategies appearing below the bisector are very effective: they involve less doses than the reference with which they are compared but results in a greater number of cases averted per dose.

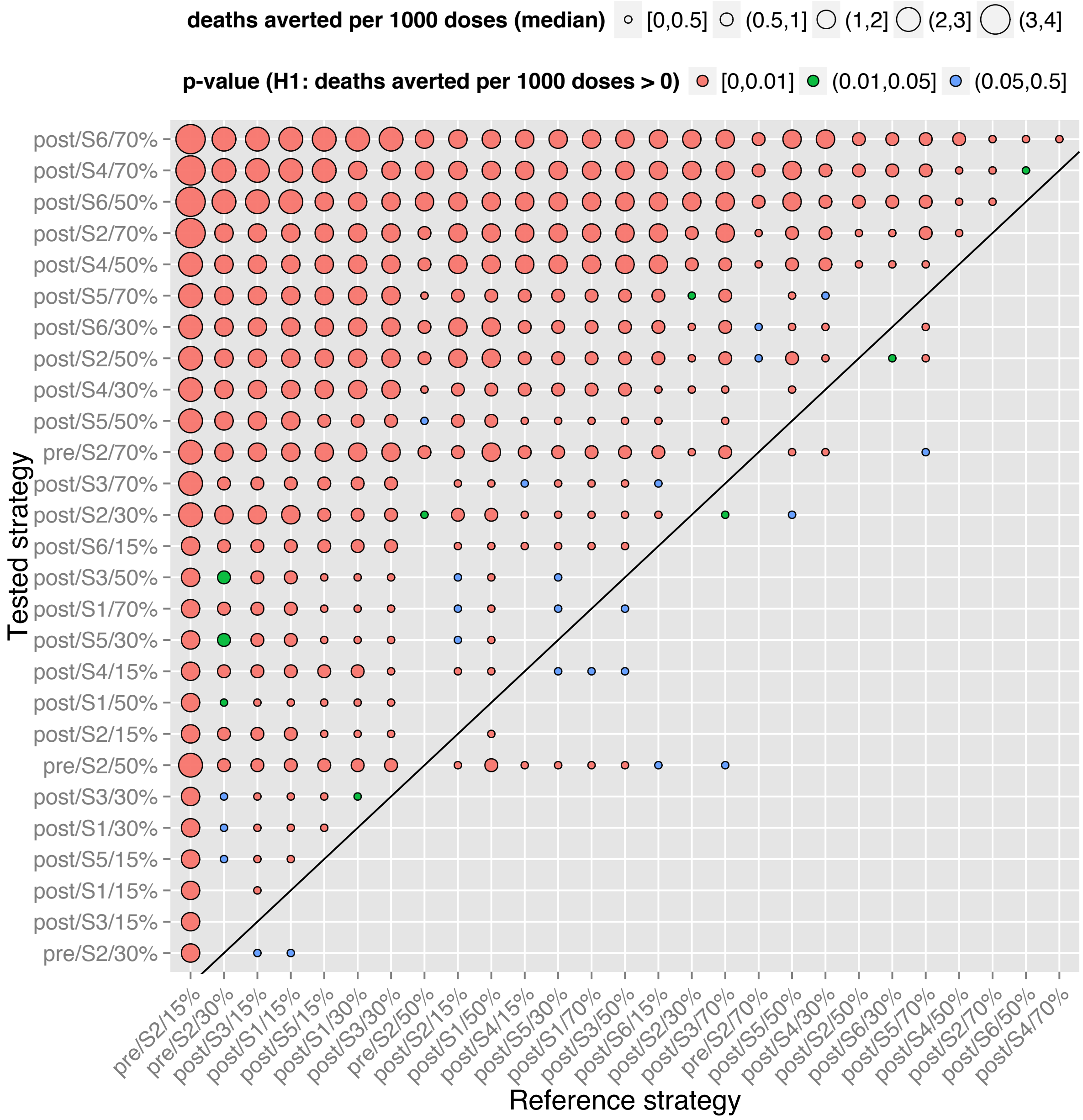

Figure S59: Computation of the pairwise significance of the differences observed between strategies in term of deaths averted. Strategies are ordered by increasing number of doses given. A circle indicates that the tested strategy averts deaths compared with the reference strategy. The size of circle gives the range of the difference, the colour indicates the significance of the difference between the two posterior distributions of the strategies. Red disks show high significance with p-values < 0.01, green disks show significance with p-values < 0.05, and blue disks show non significant differences with p-values between 0.05 and 0.5. The black diagonal line represents the bisector, tested strategies appearing below the bisector are very effective: they involve less doses than the reference with which they are compared but results in a greater number of deaths averted per dose.

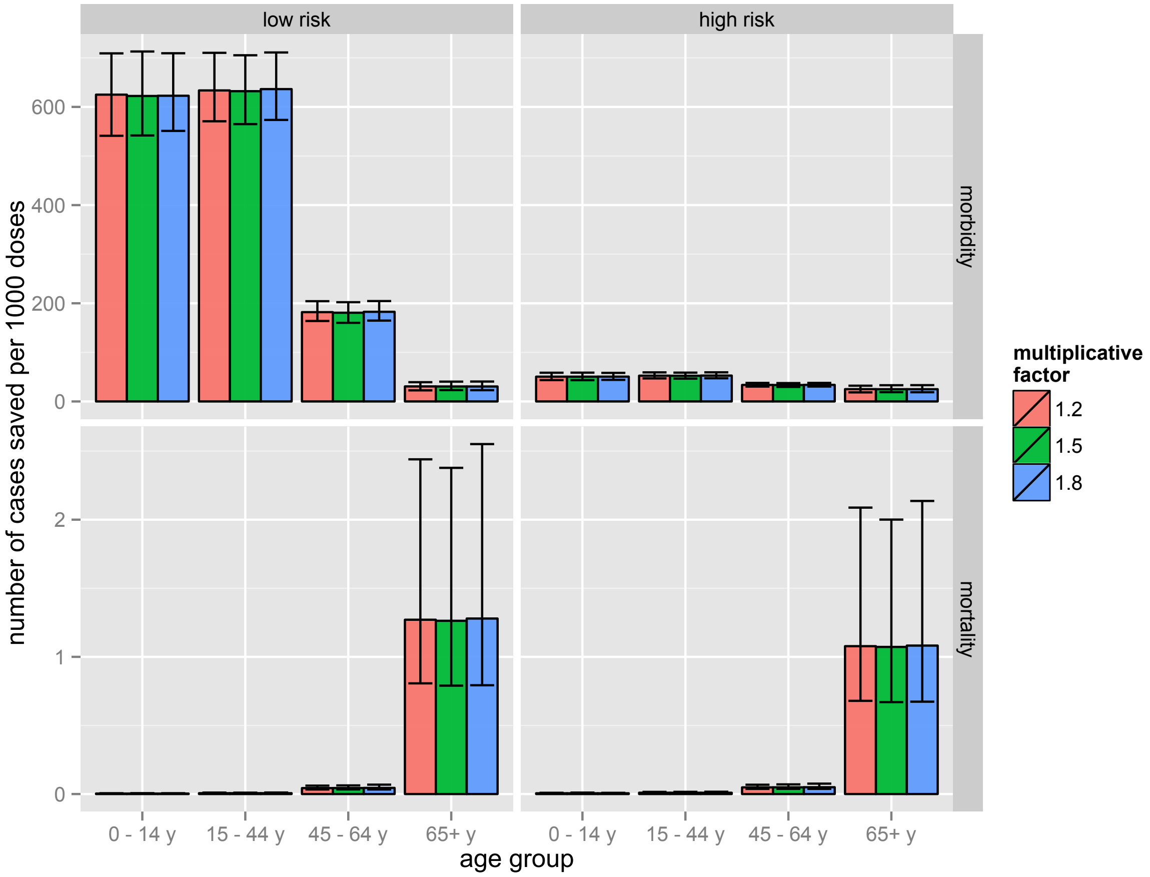

Figure S60: Computation of the number of infections and deaths averted by vaccination is not sensitive to the change of variance in the transmissibility prior.

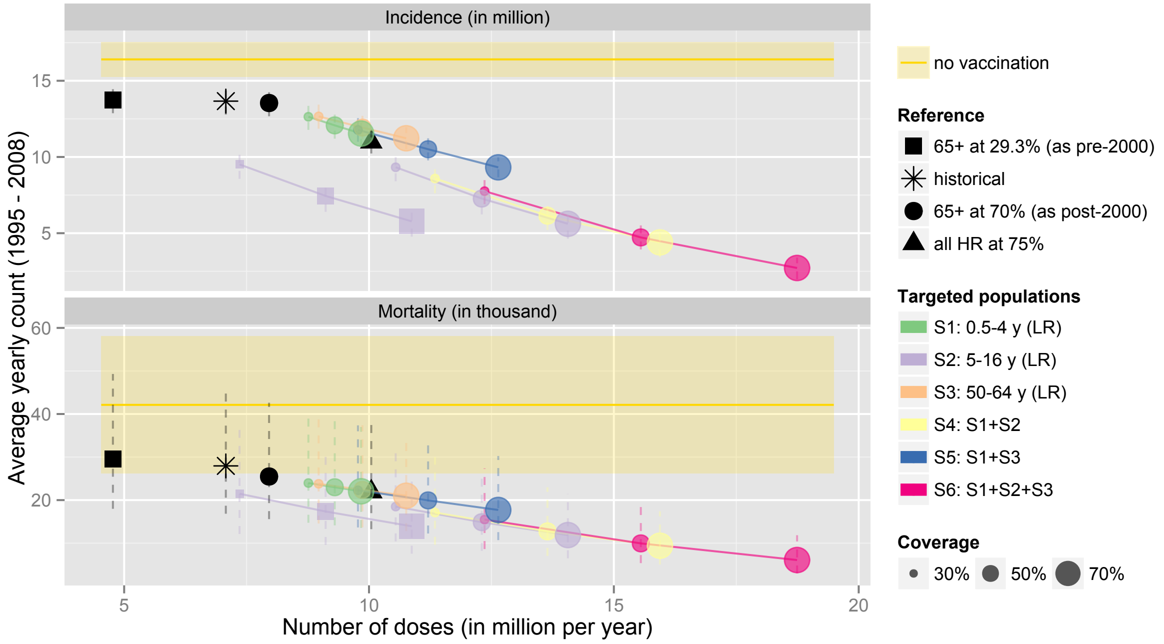

Figure S61: Effectiveness of the alternative programmes when assuming that the vaccine efficacy of the 65+ is the same than the one of the other age groups.

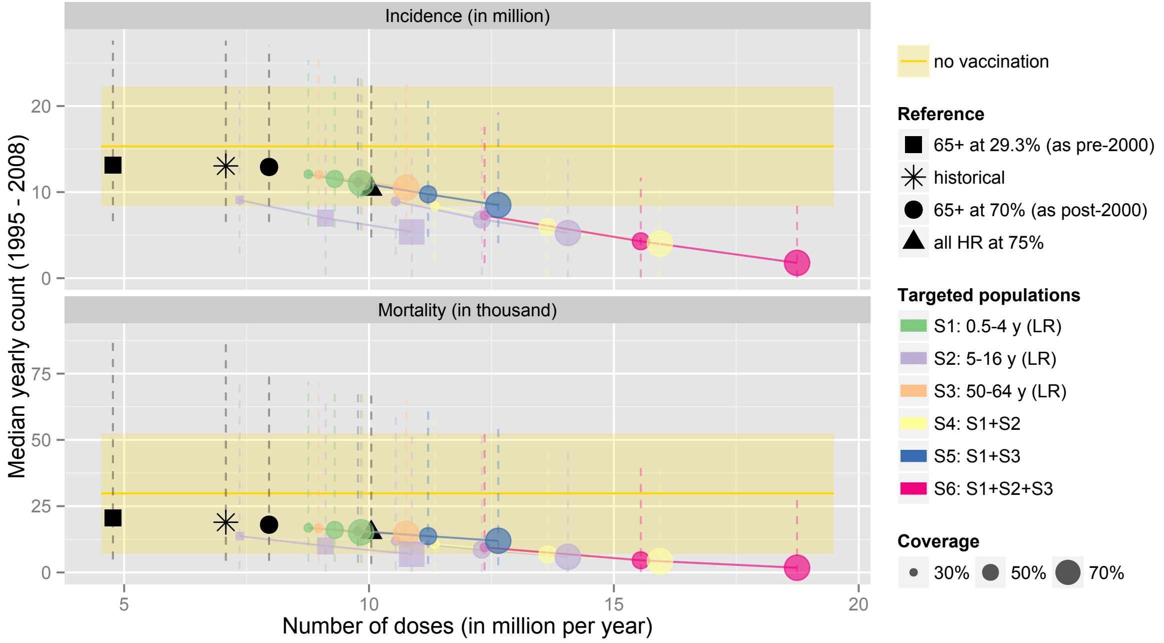

Figure S62: Effectiveness of the alternative programmes when taking into account the epidemiological uncertainty for each year.
